# A robust physics-constrained neural operator framework for efficient geothermal resource development

**DOI:** 10.1038/s41467-026-73183-0

**Published:** 2026-05-15

**Authors:** Zhenqian Xue, Jianfei Bi, Haoming Ma, Zhe Sun, Zhangxing Chen

**Affiliations:** 1 Department of Chemical & Petroleum Engineering, University of Calgary, Calgary, AB Canada; 2 Research Institute of Petroleum Exploration and Development (RIPED), PetroChina, Beijing, China; 3 School of Energy Resource, University of Wyoming, Laramie, WY USA; 4 Ningbo Key Laboratory of Low-Carbon Hydrogen Energy, Eastern Institute of Technology, Ningbo, China; 5 National Key Laboratory of Petroleum Resources and Engineering, China University of Petroleum (Beijing), Beijing, China

**Keywords:** Energy modelling, Geothermal energy, Energy infrastructure

## Abstract

Efficient evaluation of geothermal systems is critical for reliable and scalable development. However, conventional workflows are often constrained by the high computational cost of numerical simulations and the limited fidelity and generalizability of surrogate models. Here we present a physics-constrained neural operator framework that enables rapid, high-resolution, and physically consistent system-level evaluation of geothermal performance. By learning the solution operator of governing partial differential equations under physical constraints, the method captures both subsurface dynamics and surface energy production across diverse geological heterogeneity, reservoir conditions, and operational strategies. It achieves an average relative error of 1.76% over more than 7×10⁸ reservoir prediction points and 1.70% for over 6×10^4^ target variables, while delivering approximately 1,400-fold acceleration compared to conventional numerical methods. By further integrating modules for power output estimation and economic evaluation, the framework enables consistent techno-economic assessment across multiple geothermal applications. This capability supports rapid ensemble-based analyses, including resource assessment, uncertainty quantification, and multi-objective optimization, providing a scalable pathway for geothermal development.

## Abstract (web summary)

The work presents a physics-constrained neural operator framework that rapidly predicts geothermal reservoir and surface performance, enabling efficient technical and economic assessment across diverse geological and operational scenarios.

## Introduction

Geothermal energy is a sustainable and environmentally friendly source of electricity and heat. Global geothermal resources, including both conventional hydrothermal systems and enhanced or advanced geothermal systems, are estimated to be 100 to 1000 times greater than those of fossil fuels^1^. Unlike wind and solar energy, which depend on weather and environmental conditions, geothermal energy offers a stable and continuous supply^2^. This vast resource base and inherent reliability position geothermal energy as a promising candidate for next-generation energy systems^3^. However, geothermal heat and electricity production in 2020 was equivalent to only a few days of global oil production, and as of 2023, the global installed geothermal power capacity remained at 19 GW, compared with the 507 GW capacity combined from wind and solar^4–6^. For hydrothermal systems, one major barrier is the uncertainty and inefficiency in evaluating reservoir performance, operational feasibility, and economic viability^7^. For engineered systems, drilling and stimulation technologies are key challenges, but efficient technical and economic assessment is still essential to reduce project risk and guide design^8^. These factors underline an urgent need for an evaluation tool that can provide fast and reliable prediction of system-level, multi-objective performance to support project feasibility assessment and accelerate global geothermal development^9^.

Conventionally, a complete techno-economic assessment workflow for geothermal systems relies on multiple software platforms and collaboration among different experts. Geologists estimate reservoir thermal potential, engineers predict heat and power production, and economists then assess project feasibility. This multi-step process substantially increases the time, complexity, and cost of development planning, especially when numerical simulations are used to predict production performance. Although such simulators are reliable, their high computational costs often make them impractical for timely decision-making^10^, particularly for complex geothermal systems that involve coupled fluid flow, convective and conductive heat transfer, thermal losses along wellbores, and evolving fluid properties^11^. In addition, traditional simulations are typically case-specific, with each model representing a single development configuration^12^. For many geothermal sites that are still under exploration and require evaluation of many development options, conventional workflows, therefore, demand the manual construction and analysis of hundreds or thousands of models. These constraints also propagate into the economic assessment, which usually depends on outputs from technical simulations and thus becomes both labour-intensive and time-consuming^13^. Recent work has shown that unified platforms such as the Delft Advanced Research Terra Simulator (DARTS) can enable faster techno-economic uncertainty analysis for geothermal systems^14^. However, these frameworks are still commonly restricted to a limited set of uncertain operational parameters and simplified heat use representations, which restricts their ability to systematically evaluate the potential of a given reservoir under different development pathways, such as heat supply or electricity generation.

Efficient and reliable surrogate models that can reproduce the evolution of complex geothermal systems and predict production performance are, therefore, critical for improving the capability and efficiency of evaluation tools. Artificial intelligence (AI) has emerged as a promising alternative to numerical simulators for these tasks. Trained on numerical simulation data, task-specific machine learning models have been developed to estimate heat recovery, electricity generation, and economic metrics with high accuracy and speed^15–20^. However, these models face challenges in limited interpretability and generalizability, making it difficult for engineers to interpret predicted results without explicit insights into reservoir pressure and temperature dynamics.

Fourier Neural Operators (FNOs) have recently shown strong potential for subsurface flow problems by offering high generalization and interpretability^21^. Unlike traditional neural networks, the FNO learns a continuous mapping between functional inputs and a solution space, providing better convergence than other physics-based models such as Physics-Informed Neural Networks^21^. FNO-based models have achieved high accuracy in modeling CO₂-water and CO₂-oil flows, as well as CO₂ geological storage^22–25^. In geothermal applications, FNO models have been used mainly to predict subsurface fields such as pressure or temperature^26–29^. However, these models typically consider a narrow set of sensitive parameters. For example, reservoir conditions are often restricted to permeability or temperature fields, without explicitly including other key properties that affect heat extraction, for example, initial pressure distributions and rock thermal properties. On the operational side, some models account for well controls such as an injection rate, injection temperature and production pressure, but they usually assume fixed well number, well depth, well location and constant control schedules. Under these assumptions, models are applicable only to a limited set of reservoirs and development strategies. In addition, most current geothermal surrogates focus only on subsurface pressure and temperature and cannot directly provide surface-level outputs. While reservoir performance has been widely used for resource assessment and production forecasting, unavoidable heat losses in wellbores mean that surface-level predictions are essential for more reliable assessments. Furthermore, many models still operate on 2D reservoir domains and neglect vertical fluid and heat transport, which reduces their ability to represent realistic heat extraction processes and can degrade prediction accuracy. These limitations highlight the need for efficient and robust geothermal surrogate models with strong generalization at the field scale.

To address these gaps and provide a unified, efficient, and reliable tool for geothermal system evaluation, we propose a physics-constrained neural operator (PCNO) framework that targets five key limitations of current approaches: (1) the time intensity of conventional numerical simulators; (2) the limited generalization capacity of existing geothermal surrogate models and evaluation tools; (3) the difficulty of consistently capturing key physical mechanisms across coupled geothermal processes; (4) the lack of simultaneous prediction of subsurface and surface outputs and indicators for diverse geothermal applications; and (5) the challenge of resolving production phase evolution in a full 4D domain. Within this framework, we develop a coupled architecture that integrates an artificial neural network (ANN) with U-Net-embedded Fourier neural operators (U-FNOs) and enforces physics-based constraints derived from the governing partial differential equations. This design enables rapid, high-resolution, and physically consistent modeling of geothermal system behaviour, jointly predicting transient subsurface fields and surface performance indicators. Trained on a dataset that spans a wide range of reservoir, rock, and operational conditions, the PCNO provides robust predictions across heterogeneous conditions and operating strategies within the considered parameter ranges. It is further integrated with power estimation and economic evaluation modules, enabling fast execution of analyses that are traditionally computationally intensive, including multi-end-use energy forecasting, recoverable energy potential assessment, techno-economic screening, and multi-objective development optimization. Overall, this framework provides a scalable and versatile approach for accurate, physics-consistent, and efficient geothermal system evaluation, supporting rapid and informed decision-making for geothermal development.

## Results

### Dataset overview

We investigate a 20-year circulated water injection strategy for geothermal energy extraction in 3D reservoirs. All data samples are generated using the advanced thermal simulator CMG-STARS. The resulting dataset provides a comprehensive mapping between input parameters and system responses, capturing both subsurface and surface heat extraction performance across a wide range of reservoir conditions and operational strategies.

The input variables are grouped into reservoir conditions and operating schemes. Reservoir conditions include six reservoir properties (i.e., reservoir depth, pressure gradient, temperature gradient, permeability, porosity, and target reservoir thickness) and two rock properties (i.e., heat capacity and thermal conductivity). Operating schemes include nine factors (i.e., number of injectors and producers, well locations, well depths, well spacing, injection rate, injection temperature, and production pressure). For the injection rate, both constant and time-varying schedules are considered. A Monte Carlo sampling strategy is used to generate realizations, with each parameter independently sampled from a uniform distribution over its prescribed range. The description of parameter ranges is provided in Supplementary Method 1.

In PCNO, four parameters (i.e., reservoir depth, target reservoir thickness, rock thermal conductivity, and rock heat capacity) are treated as scalar variables. Pressure and temperature gradients are combined with depth and thickness to construct initial pressure and temperature fields. These fields, together with the remaining engineering variables, are treated as field variables to capture geological and operational heterogeneity. To reduce data dimensionality and enhance model efficiency, the number and location of wells, well depths, and well spacing are encoded as a single categorical “well map”. In this map, injectors are assigned a value of 1 and producers a value of −1. The horizontal distances between injector and producer cells represent well spacing, while the vertical distribution of 1 and −1 values indicates completion depths. In addition, six external variables are used to describe spatial and temporal structure, including two distance maps to injectors and producers, three spatial encoding maps and one temporal encoding map. The outputs include two reservoir state variables (i.e., temperature and pressure) and three task variables, including two production metrics (surface production temperature, thermal energy output) and injector bottom hole pressure (BHP), which is critical for operational monitoring. All outputs are stored at yearly intervals over the 20-year operating period. A detailed description of the dataset is provided in Table 1.

**Table 1:** Characteristics of samples in our datasets

Parameter	Number	Variable type	Distribution	Unit
a. Input variables				
Reservoir conditions				
Reservoir depth	1	Scalar	[1500, 3500]	m
Pressure gradient	/	/	[7, 15]	MPa km^–1^
Temperature gradient	/	/	[25, 75]	°C km^–1^
Initial pressure	1	Field	[12, 48]	MPa
Initial temperature	1	Field	[90, 250]	°C
Permeability field	1	Field	Heterogeneous	md
Porosity field	1	Field	Heterogeneous	–
Target reservoir thickness	1	Scalar	[50, 500]	m
Rock heat capacity	1	Scalar	[1.8 × 10^6^, 3.3 × 10^6^]	J m^−3^ °C^−1^
Rock thermal conductivity	1	Scalar	[8.5 × 10^4^, 3.4 × 10^5^]	J m^−1^ day^−1^ °C^-1^

Operational parameters					
Well map	Number of injectors	1	Field	[1, 4]	–
	Number of producers			[1, 4]	–
	Well location			Randomly placed	–
	Injector depth			[depth, depth - thickness]	m
	Producer depth			[depth, depth - thickness]	m
	Well spacing			[200, 600]	m
Average injection rate		1	Field	[10, 100]	Kg m^–3^
Injection temperature		1	Field	[20, 70]	°C
Production pressure		1	Field	[5, 47]	MPa
**External variables**					
Distance maps to wells		2	Field	[0, 1]	m
Spatial encoding maps		3	Field	[0, 1]	–
Temporal encoding maps		1	Field	[0, 1]	–
b. Output variables					
**Reservoir states**					
Reservoir pressure		1	Field	Predicted	MPa
Reservoir temperature		1	Field	Predicted	°C
**Task variables**					
Surface production temperature		1	Scalar	Predicted	°C
Surface thermal output		1	Scalar	Predicted	MWh_th_
Injector bottom hole pressure		1	Scalar	Predicted	MPa

As a result, each data sample contains 18 inputs and 5 outputs. In total, 9450 data samples are generated, of which 7350 are used for training, 1050 for validation, and 1050 for testing. This dataset enables the trained PCNO model to serve as a surrogate simulator alternative that can predict key reservoir dynamics, production behavior, and monitoring indicators across diverse geothermal scenarios.

### PCNO model architecture

The PCNO architecture integrates two machine learning models, ${f}^{P}$ and ${f}^{T}$, with a set of governing PDEs to predict the geothermal production performance. Each model employs a hybrid architecture (A-U-FNO) that combines the Artificial Neural Network (ANN) with U-Net embedded Fourier Neural Operators (U-FNO). The architecture of the PCNO is shown in Supplementary Fig. 3. These models are designed to learn spatial-temporal dynamics of reservoir pressure and temperature, while being constrained by physical laws through embedded governing equations, including mass and energy conservation, thermophysical fluid property relations, radial inflow equations, and wellbore dynamics.

The computational domain is defined as a 4D space-time domain with a 3D spatial reservoir domain and a 20-year temporal period. Within this architecture, scalar variables are first processed by a Global Parametric Fusion (GPF) module, which utilizes a two-layer ANN. These embeddings are concatenated with field variables to form a unified input tensor, which is subsequently fed into a sequence of U-FNO layers. Detailed model specifications are provided in Supplementary Method 3 and Supplementary Table 1.

To ensure physical consistency and improve prediction quality, the governing PDEs are integrated as physics-informed loss terms for the outputs from ${f}^{P}$ and ${f}^{T}$. Mass and energy conservation equations are applied to enforce fundamental physical constraints, while fluid property models capture the temperature- and pressure-dependent behavior of density, viscosity, and enthalpy. Radial inflow and wellbore dynamic equations are used to characterize interactions between the well and reservoir and the associated wellbore dynamics, and to compute task-specific variables.

**Fig. 1:**
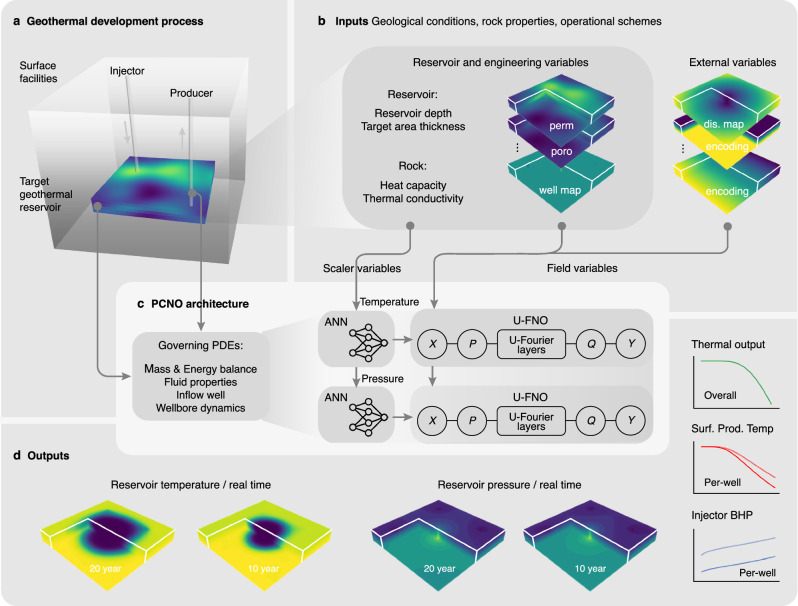
Introduction to the PCNO model. **a** Schematic of the geothermal energy development process. **b** Model inputs comprising geological, rock, and operating parameters, categorized into two types: scalar and field variables. **c** PCNO architecture. Scalar variables are first processed through an ANN and then combined with field variables as inputs to the U-FNO. Governing physical laws are embedded to constrain model predictions. **d** Model outputs include spatiotemporal distributions of reservoir pressure and temperature, along with three real-time task variables (both overall and per-well performance) critical for geothermal system evaluation. Parameters: perm - permeability; poro - porosity; dis. map - the distance maps between cells and wells; Surf. Prod. Temp - surface production temperature.

During training, a composite loss function consisting of data-driven terms and physics-based residuals is used to iteratively update the model parameters through backpropagation. This joint optimization allows the model to converge toward solutions that are not only numerically accurate but also physically plausible, thereby ensuring strong generalization across diverse geothermal scenarios. The workflow of the PCNO model is illustrated in Fig. 1.

### Model prediction performance

Geothermal energy development involves complex interactions between reservoir, fluid, and wellbore systems. The injection of cold fluids causes dynamic changes in reservoir pressure and temperature fields, which affect fluid and heat transport and in turn modify key fluid properties such as density, viscosity, heat capacity and enthalpy^30^. In addition, thermal exchange between the produced hot fluids and surrounding formations during upward transport causes heat loss in the wellbore, resulting in differences between downhole and surface conditions^31^. These coupled processes are sensitive to both reservoir properties and operating strategies.

**Fig. 2:**
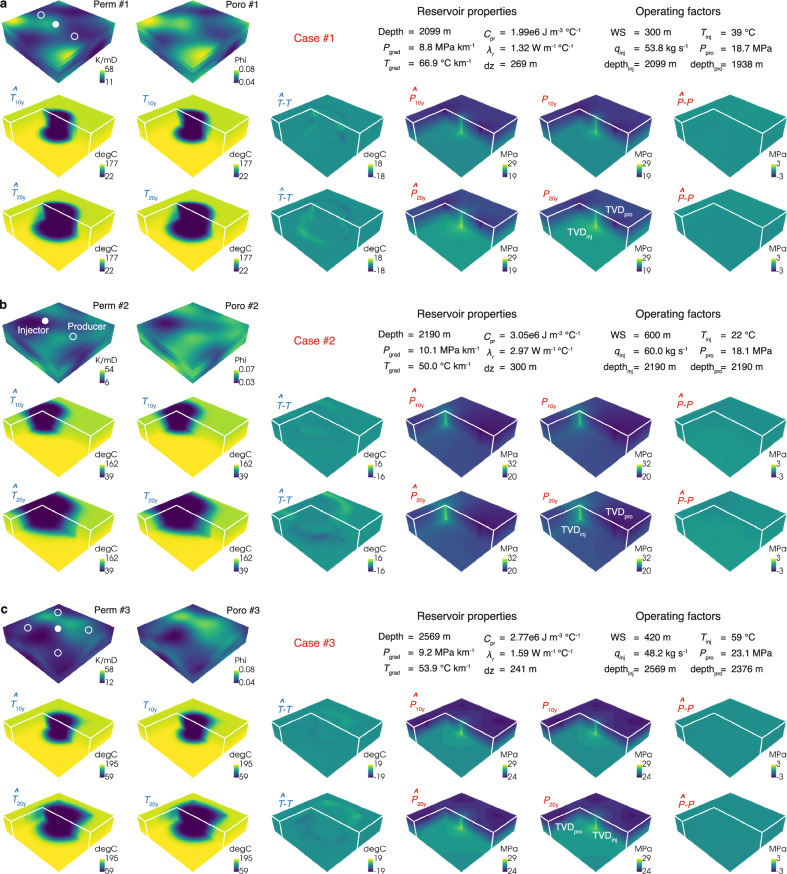
Comparison between PCNO predictions and ground truth for reservoir temperature and pressure at 10^th^ and 20^th^ years under three representative scenarios. **a** Case #1. **b** Case #2. **c** Case #3. Predicted temperature and pressure fields ($\hat{T}$ and $\hat{P}$) are compared with reference simulations ($T$ and $P$), with corresponding error maps ($T-\hat{T},$ $P-\hat{P}$). Parameters: perm - permeability; poro - porosity; Depth - reservoir depth; ${P}_{{{{\rm{grad}}}}}$ - pressure gradient; ${T}_{{{{\rm{grad}}}}}$ - temperature gradient; ${C}_{{{{\rm{pr}}}}}$ - rock heat capacity; ${\lambda }_{r}$ - rock thermal conductivity; dz - target zone thickness; WS - well spacing; ${q}_{{{{\rm{inj}}}}}$ - injection rate; ${T}_{{{{\rm{inj}}}}}$ - injection temperature; ${P}_{{{{\rm{pro}}}}}$ - production pressure; ${{{{\rm{depth}}}}}_{{{{\rm{inj}}}}}$ and ${{{{\rm{depth}}}}}_{{{{\rm{pro}}}}}$ - injector and producer depths; ${{{{\rm{TVD}}}}}_{{{{\rm{inj}}}}}$ and ${{{{\rm{TVD}}}}}_{{{{\rm{pro}}}}}$ - true vertical depths of injector and producer. Note: Permeability and porosity are shown for the full reservoir, while temperature and pressure are displayed in a clipped volume to highlight vertical variations.

By incorporating governing PDEs that describe reservoir, fluid, and wellbore behavior, the PCNO effectively captures these interactions and provides accurate real-time predictions of reservoir performance. To demonstrate its predictive capability, we present three representative scenarios showing the spatial-temporal evolution of reservoir pressure and temperature (Fig. 2). The plots are clipped within a selected volume so that vertical variations can be seen clearly. Across diverse geological and operational conditions, the PCNO reproduces the spread of pressure in both horizontal and vertical directions, consistent with expected flow and heat transport during production. It identifies preferential flow paths in high-permeability regions and matches pressure changes near wells caused by contrasts in formation conductivity (Fig. 2a, c). The model also captures the effect of injection rate, with higher injection scenarios exhibiting larger pressure increases (Fig. 2a, b) and lower rates producing more moderate changes (Fig. 2c). Detailed comparisons in the horizontal (XY) and vertical (XZ) planes are provided in Supplementary Fig. 4 and 5. The model prediction performance on the changeable injection schedule is provided in Supplementary Fig. 6.

**Fig. 3:**
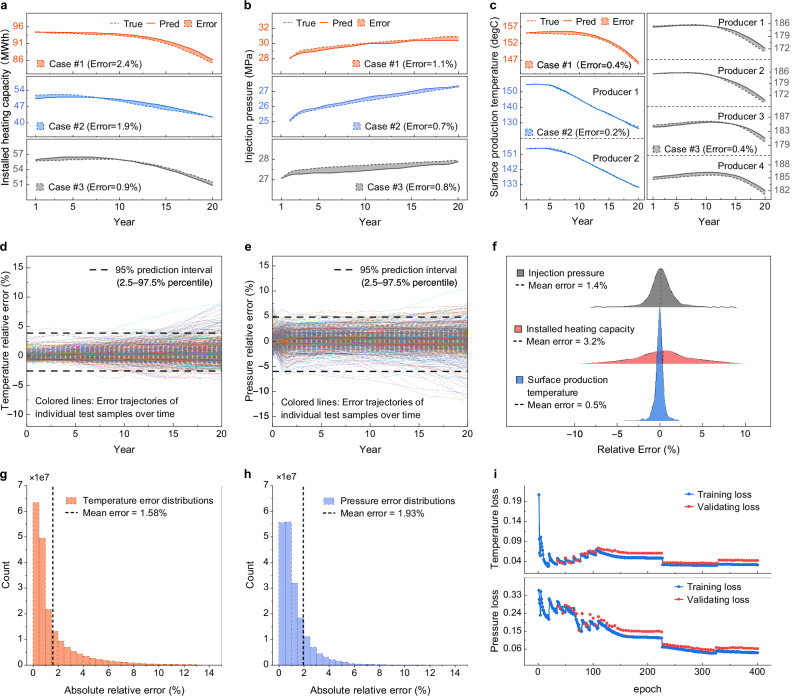
Performance evaluation of the PCNO model. **a** Installed heating capacity for three representative cases shown in Fig. 2. **b** Bottom-hole pressure at each injection well for the three cases. **c** Surface production temperature at each production well for the three cases. **d, e** Relative errors in reservoir temperature and pressure over time across test samples. **f** Kernel density estimates of relative errors and absolute mean relative error values for the three task variables across test samples. **g** Distribution of relative errors in reservoir pressure across all prediction points in the test samples. **h** Distribution of relative errors in reservoir temperature across all grid points in the test samples. **i** Training and validating losses of the pressure and temperature modules over training epochs. Parameters: True - simulation references; Pred - PCNO predictions; Error - mean variance between simulation references and PCNO predictions.

In addition to reservoir state predictions, the PCNO also exhibits high fidelity for task-specific metrics. With wellbore equations embedded, it captures the time evolution of surface production temperature and installed heating capacity (Fig. 3a, c) and provides reliable estimates of subsurface well metrics such as injector BHP (Fig. 3b). Importantly, the model predicts both field-scale outputs and individual well performance. For example, the predicted surface production temperature of each well closely matches the reference values. Across all three representative cases, the relative errors for task-specific variables remain within acceptable limits.

Overall model accuracy is summarized in Fig. 3d–i. At each time step, relative errors in the predicted reservoir pressure and temperature remain consistently low (Fig. 3d, e). Over more than 7 × 10^8^ prediction points, the PCNO achieves grid-averaged mean relative errors of 1.58% for temperature and 1.93% for pressure (Fig. 3g, h). For task-specific outputs, average relative errors are 1.4% for injector BHP, 0.5% for surface production temperature, and 3.2% for installed heating capacity across all test cases (Fig. 3f). Due to the staged training procedure, validation losses are monitored from the 30^th^ epoch, and the results indicate minimal overfitting and stable generalization performance (Fig. 3i).

In summary, the PCNO provides accurate, time-resolved predictions of subsurface states and surface production metrics within the range of considered reservoir and operating conditions. This capability makes the PCNO a computationally scalable tool to support geothermal resource and system potential assessment, production forecasting, techno-economic evaluation, and the evaluation and preliminary design of monitoring and control strategies in geothermal projects.

### Recoverable geothermal energy potential assessments

The fast predictive capabilities of the PCNO enable efficient large-scale ensemble modeling and probabilistic assessment. Evaluating geothermal energy potential requires complex analysis of thermal flux, geological structure and hydrogeological properties. However, most existing approaches primarily focus on subsurface thermal potential and often overlook or simplify operational uncertainties in real-world developments, leading to limited insight into recoverable thermal energy. In addition, although electricity generation is a major use of geothermal resources, current methods rarely provide quantitative estimates of both heat and power output in a consistent framework. These quantities are often inferred from approximate thermal assessments for individual cases using different tools. Consequently, operators may face uncertainty when deciding whether a given reservoir is better suited for heat supply or electricity generation.

**Fig. 4:**
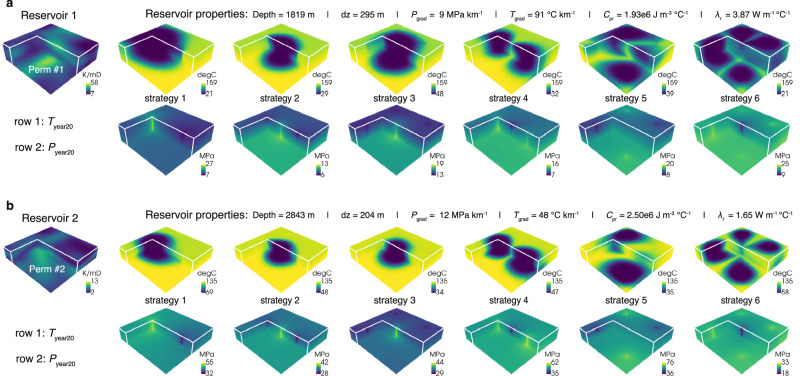
Reservoir temperature and pressure predictions under six operational configurations for two geothermal reservoirs. **a** Reservoir 1. **b** Reservoir 2. Parameters: perm–permeability; Depth–reservoir depth; dz– target zone thickness; ${P}_{{{{\rm{grad}}}}}$ - pressure gradient; ${T}_{{{{\rm{grad}}}}}$ - temperature gradient; ${C}_{{{{\rm{pr}}}}}$ - rock heat capacity; ${\lambda }_{r}$ - rock thermal conductivity; ${T}_{{{{\rm{year}}}}20}$ - predicted temperature field at year 20; ${P}_{{{{\rm{year}}}}20}$ - predicted pressure field at year 20. Note: Details of the operating strategy settings for each reservoir are provided in Supplementary Table 4.

To address these limitations, we integrate a power estimation module into the PCNO framework, enabling simultaneous predictions of both thermal and electrical energy output. As a demonstration, we conduct probabilistic assessments of the heat and power potential for two geothermal reservoirs. For each reservoir, 1000 development cases are created by Monte Carlo sampling over key operating parameters, including well configuration and controls, within the ranges listed in Supplementary Table 4. Reservoir conditions and corresponding PCNO-predicted reservoir responses for six representative scenarios in each reservoir are shown in Fig. 4. By capturing the combined nonlinear effects of reservoir properties and operating schemes on energy extraction, the PCNO allows rapid screening of development plans and provides a basis for estimating recoverable heat and power. On an NVIDIA A100-PCIe GPU, predictions for all 2000 cases are completed in approximately 650 s.

**Fig. 5:**
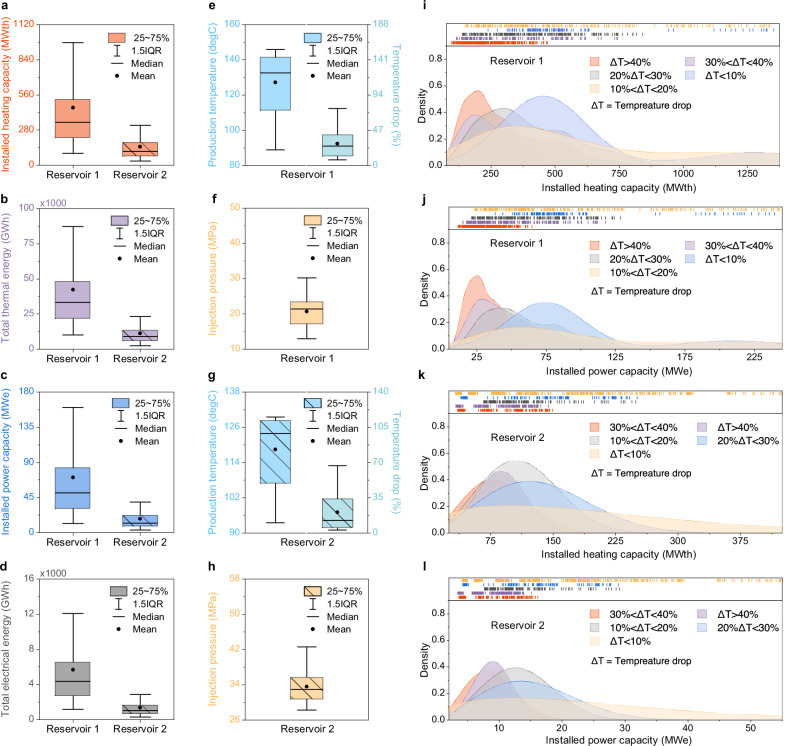
Assessment of energy extraction potential for Reservoir 1 and Reservoir 2. **a** Distribution of installed heating capacity for two reservoirs. **b** Distribution of total thermal energy production for two reservoirs. **c** Distribution of installed power capacity for two reservoirs. **d** Distribution of total electrical energy production for two reservoirs**. e**, **g** Distribution of average production temperature and temperature loss for two reservoirs. **f**, **h** Distribution of bottom hole pressures at injection wells for two reservoirs. **i**, **k** Distributions and density plots of installed heating capacity across different temperature drop ranges for two reservoirs. **j**, **l** Distributions and density plots of installed power capacity across different temperature drop ranges for two reservoirs.

Figure 5 shows the probabilistic results for both reservoirs, including the distributions of installed heating and power capacities and total thermal and electrical energy. By covering a wide range of operating strategies, these distributions reflect the performance range achievable under different development plans. Summary statistics (e.g., mean, median, interquartile range) assist to characterize production potential (Fig. 5a–d). The PCNO also provides probabilistic distributions of surface production temperature and temperature drop (Fig. 5e, g), and injector BHPs (Fig. 5f, h), which support assessments of reservoir sustainability and operating feasibility.

These outputs support more informed decision-making. For example, production temperature distributions help identify suitable applications (e.g., heating or electricity generation) and guide the selection of surface facilities such as power plant type. Estimates of temperature decline offer insights into expected project lifetimes. The PCNO also allows development scenarios to be filtered according to project-specific limits, such as maximum acceptable temperature drop or bounds on BHP. As an illustration, Fig. 5i–l shows installed capacities under different thresholds for temperature decline. These results not only quantify production potential but also offer practical insights. For instance, in these two reservoirs, the results suggest that operators should avoid focusing solely on minimizing temperature drop to extend project life, since cases with very small temperature drop (<10%) may have insufficient heat extraction rates and limited energy output.

Overall, integrating the power estimation module into the PCNO framework enables efficient and constraint-aware probabilistic assessments of geothermal potential. The approach provides a combined view of thermal and electrical outputs together with key development metrics, offers valuable insights into reservoir dynamics, operational efficiency, and infrastructure suitability, and supports data-driven decisions on project evaluation, strategy selection and deployment of geothermal systems.

### Economic evaluation and development plan optimization

The relationships between heat extraction performance and operational parameters in geothermal systems are highly nonlinear, making their interactions difficult to characterize and efficient field-level optimization challenging. In addition, given the technical complexity and financial risks of geothermal projects, optimization must be treated as a multi-objective problem that balances technical performance and economic viability.

To enable efficient multi-objective optimization under constraints such as operational sustainability, we integrate an economic evaluation module into the PCNO to calculate the levelized costs of heat (LCOH) and electricity (LCOE). A composite score function is defined based on both technical and economic performance, with higher scores assigned to configurations that bring greater energy output and lower LCOH or LCOE. The detailed formulation is provided in Supplementary Method 5.

**Fig. 6:**
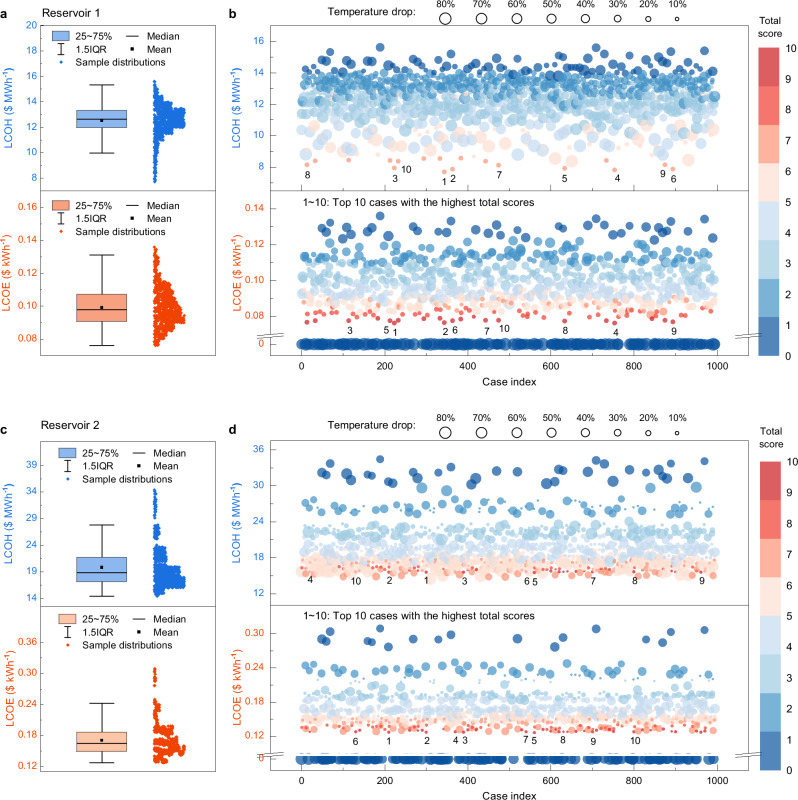
Probabilistic assessment of economic performance and development plan optimization. **a** Distributions of LCOH and LCOE with sample realizations for 1000 operational scenarios in Reservoir 1. **b** LCOH and LCOE across all cases, colored by total score and sized by temperature drop, with top-performing cases highlighted in Reservoir 1. **c** Distributions of LCOH and LCOE with sample realizations for 1000 operational scenarios in Reservoir 2. **d** LCOH and LCOE across all cases, colored by total score and sized by temperature drop, with top-performing cases highlighted in Reservoir 2. Note: The definition of total score is provided in Supplementary Method 5.

As shown in Fig. 6, we conduct field-scale optimization for the two geothermal reservoirs included in Fig. 4. The framework presents both objectives and constraints across the entire set of operational strategies, allowing operators to rapidly identify optimal operating schemes under custom criteria such as limits on reservoir temperature decline. For example, by setting a temperature decline constraint (e.g., <10%), the top 10 highest-scoring strategies that satisfy this condition can be selected directly. Because optimization outcomes may depend on the intended end use, the framework is also able to perform separate optimizations for heat use and power generation. The resulting use-specific recommendations, summarized in Table 2, directly support development planning for projects with different objectives.

**Table 2:** Optimal operational schemes for heating and electricity generation

a. Reservoir #1												
Heating							Electricity generation					
Rank	Well map	Inj. R.	Inj. T.	Pro. P.	LCOH	Ins. H.	Well map	Inj. R.	Inj. T.	Pro. P.	LCOE	Ins. P.
1	(1, 4)/420	61.2	45	12.8	7.70	1357.4	(2, 2)/576	35.2	32	12.5	0.0765	189.3
2	(1, 4)/432	35.2	32	12.5	7.82	1329.9	(4, 1)/456	55.8	22	11.8	0.0769	188.1
3	(4, 1)/492	71.8	52	12.6	7.89	1348.0	(1, 4)/420	61.2	45	12.8	0.0763	185.3
4	(2, 2)/600	39.0	62	12.4	7.89	1337.5	(1, 4)/408	26.0	20	11.5	0.0776	187.0
5	(1, 4)/408	60.6	59	12.3	7.92	1327.2	(4, 1)/492	71.8	52	12.6	0.0775	185.5
6	(4, 1)/456	55.8	22	11.8	7.96	1281.9	(1, 4)/408	60.6	59	12.3	0.0779	186.3
7	(1, 4)/408	41.0	70	12.2	8.13	1330.6	(1, 2)/324	52.0	25	10.4	0.0778	184.8
8	(2, 2)/576	28.2	64	12.1	8.15	1320.9	(2, 2)/600	38.7	40	11.1	0.0764	178.6
9	(1, 4)/408	26.0	20	11.5	8.16	1256.1	(1, 4)/408	39.0	62	12.4	0.0767	175.1
10	(2, 1)/360	31.0	59	11.6	8.37	1280.8	(4, 1)/420	50.4	26	10.3	0.0780	179.0

b. Reservoir #2												
Heating							Electricity generation					
Rank	Well map	Inj. R.	Inj. T.	Pro. P.	LCOH	Ins. H.	Well map	Inj. R.	Inj. T.	Pro. P.	LCOE	Ins. P.
1	(2, 2)/576	34.2	68	30.8	15.60	358.3	(2, 2)/576	34.2	68	30.8	0.1270	44.8
2	(1, 4)/420	35.2	29	30.2	15.53	350.6	(1, 4)/456	44.0	79	30.3	0.1276	44.4
3	(1, 4)/456	44.0	79	30.3	15.67	354.4	(1, 4)/420	35.2	29	30.2	0.1266	43.8
4	(2, 2)/600	51.8	78	30.1	15.68	352.0	(2, 2)/600	51.8	78	30.1	0.1277	44.1
5	(4, 1)/420	30.4	61	29.8	15.63	347.4	(4, 1)/420	30.4	61	29.8	0.1275	43.4
6	(4, 1)/408	27.8	77	29.7	15.70	347.8	(4, 1)/408	27.8	77	29.7	0.1280	43.5
7	(4, 1)/492	58.4	54	29.5	15.62	344.3	(4, 1)/492	58.4	54	29.5	0.1275	43.0
8	(2, 2)/552	27.6	42	29.4	15.59	342.8	(1, 4)/384	79.8	79	29.0	0.1285	41.9
9	(1, 4)/420	27.2	33	29.2	15.59	340.9	(1, 4)/360	34.2	32	28.7	0.1276	41.9
10	(2, 1)/600	51.6	35	29.2	15.60	340.8	(1, 2)/324	29.8	30	28.7	0.1276	41.7

a Determined optimal cases for Reservoir #1.

b Determined optimal cases for Reservoir #2.

Well map includes the information of well numbers and well spacing. For example, (1, 4) represents one injector and four producers, and 420 means well spacing is 420 m. Inj. R. (kg s^−1^) is the injection rate. Inj. T. (°C) is the injection temperature. Pro. P. (MPa) is the production pressure. LCOH ($ MWh^−1^) is the levelized cost of heat. Ins. H. (MWh_th_) is the installed heating capacity. LCOE ($ kWh^−1^) is the levelized cost of electricity. Ins. P. (MWh_e_) is the installed power capacity.

By combining the economic module with the PCNO, the framework also provides both guidance on optimal operating strategies and a quantitative view of economic potential. For instance, for Reservoir 1, LCOH values across 1000 simulated operating scenarios span a 25th−75th percentile range of about 11.9-13.3 $ MWh^−1^, with a median value of 12.7 $ MWh^−1^ and a mean of 12.5 $ MWh^−1^. When thermal output is sufficient for electricity generation, the corresponding LCOE ranges between 0.082 and 0.112 $ kWh^−1^.

In summary, the PCNO framework provides an efficient approach for evaluating the economic potential of geothermal reservoirs and for screening high-performing operating strategies across different development goals. By jointly considering technical performance, economic cost, and key operational constraints, it supports more supportive decision-making and helps inform the planning of geothermal projects.

### Computational speed-up

Computationally efficient evaluation tools are essential for accelerating geothermal development. Although numerical simulations offer reliable predictions, their high computational cost and the need for specialized expertise in model construction can significantly slow project progress. The PCNO addresses these limitations by serving as a fast surrogate model with multiple integrated functions. Once trained and validated, it can handle diverse input conditions for geothermal evaluation within the considered ranges and eliminates the need for labor-intensive model setup required in conventional workflows.

This computational advantage is particularly valuable for large-scale tasks such as probabilistic assessments and uncertainty assessment, which often require hundreds or thousands of scenarios to be evaluated. To quantify this benefit, we compare the prediction time of the PCNO with that of an advanced full-physics thermal simulator, CMG-STARS. PCNO testing is performed on an NVIDIA A100-PCLe GPU, which completes 1050 cases in about 310 s, including dataset loading. For the same 1050 cases, CMG STARS requires around 7380 min on an AMD Ryzen 9 5950X CPU, including simulator initialisation between runs. These time costs exclude data preprocessing, post-processing, and the effort to construct the model. In addition, the PCNO directly interprets inputs and produces outputs without the need for specialized expertise in model construction, substantially reducing the effort required to set up and run large ensembles. A further advantage is that PCNO is naturally suited to modern GPU hardware, whereas most widely used reservoir simulators are primarily designed for CPU-based computation and have limited ability to leverage GPU acceleration.

Importantly, the PCNO can simultaneously predict both technical and economic performance for different geothermal applications within a unified framework, with predictions generated in seconds to minutes. In contrast, running the 1,050 test cases in CMG-STARS provides only technical outputs, and several extra hours are required for subsequent power estimation and economic evaluation. Moreover, the computational cost of conventional simulations increases with model complexity, for example, when grid resolution is increased, reservoir heterogeneity is stronger, or coupled wellbore dynamics are included, whereas the prediction time of the PCNO is effectively independent of case complexity (Supplementary Fig. 9). Consequently, these features make the PCNO framework well-suited for efficient geothermal project screening and planning.

## Discussion

A robust evaluation tool that supports rapid prediction, comprehensive performance characterization, and decision-oriented analysis is essential for technically and economically viable geothermal development. The proposed PCNO framework addresses this need by integrating multiple functional modules within a 4D surrogate model, providing a unified platform for efficient assessment of technical performance and economic outcomes across diverse tasks.

Within an end-to-end geothermal evaluation workflow, production-phase prediction is particularly critical but remains computationally expensive when performed with high-fidelity numerical simulators. AI-based surrogates offer a promising alternative, but most existing geothermal surrogates are often restricted to a small set of outputs, narrow ranges of reservoir and operational uncertainty, fixed well configurations, and 2D domains. Given the importance of coupled horizontal and vertical heat and fluid transport, the strong variability of geothermal resources even within the same conceptual type, and the practical need to screen hundreds to thousands of operational strategies, these restrictions substantially limit the generalizability and practical utility of current approaches.

These limitations are closely linked to model architecture, data structure, and training procedure. Although FNOs have shown stronger capability than many other methods for subsurface flow problems^21^, their predictive capability remains primarily data-driven and is not configured to jointly predict reservoir states and surface-level performance within a single model. For complex geothermal systems where both reservoir behaviour and surface production performance are critical, data alone is often insufficient within a practical training budget. When a surrogate must handle many multi-dimensional inputs, particularly variable well configurations, and predict production behaviour over a full 4D domain, the required dataset size and training time can become prohibitive, and convergence to a satisfactory solution is not assured. The training workflow and loss function are also critical. For systems with many input variables, a conventional uniform training schedule and a simple loss that aggregates a few indicators may guide the model towards solutions that match aggregate metrics but fail to accurately represent detailed spatio-temporal dynamics.

The PCNO addresses these limitations through a coordinated design of architecture, data structure, and training strategy. Our experiments (Supplementary Table 3) show that augmenting the FNO with a U-Net module systematically reduces prediction errors by allowing the model to capture both global patterns and fine-scale behaviour. Embedding governing physical equations further constrains the solution space to key mechanisms, which supports accurate prediction, reduces long-term drift relative to purely data-driven baselines, and especially, extends the predictive ability of the model from reservoir-level fields to joint prediction of subsurface states and surface performance metrics. The GPF module improves the efficiency of data preprocessing and training in this high-dimensional setting without degrading accuracy. On the data side, the ‘well map’ together with a set of encodings, including distances to wells and spatial and temporal encodings, provides a unified description across different well numbers, locations, and control schemes, enhancing adaptability to changing well configurations and associated reservoir evolution. During training, the staged strategy introduces data-driven and physics-based loss terms sequentially, guiding a stable and efficient optimization path. The model first learns the broad structure of the solution, then refines predictions in critical near-well regions and improves spatial and temporal gradients, after which physical constraints are enforced as an additional loss term to confirm physical consistency. These design choices reduce training instability, improve convergence, and limit systematic biases, enabling the surrogate to handle a broader set of input variables and to reconstruct both state fields and derived quantities with higher fidelity.

These advances lead to the PCNO with improved interpretability and generalizability compared with existing geothermal surrogates. Rather than being limited to a small set of inputs and outputs in reduced dimensions, the PCNO provides time-resolved predictions of reservoir fields together with key subsurface and surface task-specific indicators for a full 4D geothermal system. It maintains stable performance across variations in the main sensitive reservoir and operating factors, especially for unfixed well configurations. Within the tested ranges, the model also generalizes to variable injection schedules, non-uniform reservoir structures, and simplified enhanced geothermal system configurations (Supplementary Figs. 6–8), rather than reproducing only specific training realizations.

Building on this surrogate capability, the PCNO further offers broader applicability compared with many existing evaluation tools that are either time-consuming or lack the generality or flexibility needed to support a full workflow in a single framework. By integrating power estimation and economic assessment modules, it can efficiently handle large-scale tasks such as probabilistic assessment, uncertainty analysis, and screening of high-performing operating strategies. By predicting system behaviour at multiple levels, from reservoir fields to task-specific metrics, it provides a multi-dimensional view of geothermal performance (e.g., production volume, system sustainability, operational feasibility, cost indicators, and suitability for different end-use applications), and thereby supports more informed decision-making rather than focusing only on heat production or a single economic metric. This capability also enables efficient multi-objective optimization under practical constraints and provides guidance on operating strategies that balance performance and risk for different end uses. Although the optimal operating ranges obtained from these analyses may not transfer directly to field practice, they offer a valuable starting point for understanding trade-offs and narrowing the search space of viable strategies. In addition, the economic and optimization modules are implemented as external and configurable components. This allows operators to modify key inputs such as capacity factor and conversion efficiency to reflect project-specific conditions and financing structures, to specify thresholds such as acceptable temperature drops or BHP limits, and to adjust the relative weights of technical and economic objectives in the scoring function.

Limitations and opportunities: This work still has several limitations. First, although the current representation of geological properties allows the PCNO to be applied to systems with different reservoir conditions, the considered ranges mean that its practical applicability is confined to single-phase, liquid-dominated systems, and its predictions for strongly two-phase systems have not been validated and are expected to be less reliable. Second, heterogeneous permeability is used to approximate spatial variations in reservoir conductivity and can represent fracture networks at a coarse scale. However, the PCNO still has difficulty reproducing more complex geologies, such as explicit faults and non-uniform fine-scale engineered fractures. Third, although the PCNO allows changing well configurations, the representation of more complex well architectures, such as a larger number of wells, deviated or horizontal wells, and more realistic control strategies, remains limited. Fourth, while the PCNO considers two-phase wellbore flow, it assumes fixed wellbore properties. These properties may vary between projects and influence the selection of surface facilities and well controls. Allowing variable wellbore properties would further strengthen robustness and generalizability. Finally, although the integration of physical PDEs allows the application across a range of reservoir sizes and the tested grid resolution provides sufficient spatial detail for most geothermal settings, this configuration may be inadequate for extremely large reservoirs, for example, on the order of 10,000 × 10,000 × 1000 m³, where limited spatial resolution can reduce the representativeness of the predictions.

These limitations mainly reflect practical constraints on data feasibility, computational cost, and the need to keep the surrogate model tractable. A unified surrogate that covers both single- and two-phase geothermal systems and a broad range of realistic well configurations would require much larger training ensembles and more tightly coupled physics. There are also a few reliable, high-fidelity datasets that can systematically represent realistic well controls, faults, and engineered fractures, which makes it difficult to construct such surrogates in a systematic way. Despite these constraints, the PCNO, particularly its integration of multiple assessment modules and the design of architecture, treatment of variable well configuration, loss function, data structure, and training procedure, provides a transferable template for surrogate modeling and surrogate-based evaluation of geothermal and other subsurface energy systems. Future work can build on this foundation in several directions. First, dedicated surrogates for two-phase geothermal systems can be developed with parameter ranges tailored to those conditions, which would reduce the required training domain while still capturing key physics, and then combined with the present PCNO to cover a broader set of resource types. Second, additional field-scale datasets and more general encodings for complex geological structures and realistic well controls are needed to further strengthen the generalization of trained models. Third, multi-resolution representations and time-series prediction capability can be incorporated to better support large reservoirs and long-term operations. Finally, additional physics such as geomechanical effects can be integrated in later extensions to extend the scope of the framework.

## Methods

### Datasets development and preprocessing

We collect data from 3D reservoir models using the well-established full-physics thermal simulator CMG-STARS. The models follow the settings of our previously validated simulations to ensure dataset reliability^30,32,33^. To capture both geological and operational uncertainties, we consider eight reservoir parameters and nine operating parameters (Table 1). These variables serve as simulation inputs to represent different geothermal reservoir conditions and development scenarios.

To accelerate the prediction process, we extract target 3D subregions from the reservoir models to enable focused and efficient estimation of reservoir performance. Two-phase wellbore flow dynamics are simulated using the built-in wellbore model in CMG-STARS. These considerations ensure that critical physical mechanisms relevant to geothermal energy development are accurately captured. Each simulation represents 20 years of geothermal production under water circulation. Five annual output variables within 20 years period are computed from each case, including two reservoir performance metrics and three key task variables (Table 1). In total, 9,450 different geothermal reservoir cases are generated.

After collecting the raw simulation data, the inputs and outputs are reformatted to align with the requirements of the PCNO model. Specifically, initial reservoir pressure and temperature distributions are used in place of geothermal and pressure gradients and reservoir depth. In addition, the numbers of injectors and producers, their depths, locations and spacing are combined into a single input referred to as the “well map.” The input variables are further categorized into two groups based on their spatial and temporal characteristics: field variables and scalar variables (Table 1).

To further assist the PCNO model in capturing spatial and temporal dependencies, we introduce six external variables, including two distance maps to injectors and producers, three spatial encoding maps, and one temporal encoding map. As a result, each data sample consists of 18 input variables and 5 output variables. All inputs and outputs, except for the external encodings, are standardized before model training. The details of the numerical model configuration, variable definition, and governing equations are provided in Supplementary Methods 1 and 2.

### Evaluation metrics for model training

Given the complex and coupled physical processes involved in geothermal energy development, we construct a composite loss function to enable the PCNO model to effectively learn the underlying mechanisms. The loss comprises three primary components: (1) the mean squared error of model predictions, ${{{{\rm{MSE}}}}}_{{{{\rm{Model}}}}}$; (2) the mean squared error of physics residuals, ${{{{\rm{MSE}}}}}_{{{{\rm{Physics}}}}}$; and (3) relative-error terms for key task variables, ${{{{\rm{RE}}}}}_{{{{\rm{task}}}}}$, including surface production temperature, produced thermal energy, and injector BHPs.

The first component, ${{{{\rm{MSE}}}}}_{{{{\rm{Model}}}}}$, includes ${{{{\rm{MSE}}}}}_{{{{\rm{P}}}}}$ and ${{{{\rm{MSE}}}}}_{{{{\rm{T}}}}}$ for the pressure and temperature models to quantify the discrepancy between predicted and reference fields. To enhance model sensitivity to both large-scale trends and local dynamics, ${{{{\rm{MSE}}}}}_{{{{\rm{Model}}}}}$ term is further decomposed into three subcomponents: standard MSEs (${{{{\rm{MSE}}}}}_{{{{\rm{P}}}}}^{{{{\rm{s}}}}}$ and ${{{{\rm{MSE}}}}}_{{{{\rm{T}}}}}^{{{{\rm{s}}}}}$) to assess overall accuracy; weighted MSEs (${{{{\rm{MSE}}}}}_{{{{\rm{P}}}}}^{{{{\rm{w}}}}}$ and ${{{{\rm{MSE}}}}}_{{{{\rm{T}}}}}^{{{{\rm{w}}}}}$) to emphasize near-well regions; and mean relative gradient losses (${{{{\rm{MRE}}}}}_{{{{\rm{P}}}}}^{{{{\rm{g}}}}}$ and ${{{\rm{M}}}}{{{{\rm{RE}}}}}_{{{{\rm{T}}}}}^{{{{\rm{g}}}}}$) to capture spatial and temporal variations in pressure and temperature fields. These subcomponents are expressed as:

1 $${{\mbox{MSE}}}^{{{\rm{s}}}}=\,\frac{1}{n}{\sum}_{i=1}^{n}{\left({Y}_{i}-{\hat{Y}}_{i}\right)}^{2}$$

2 $${{{{\rm{MSE}}}}}^{{{{\rm{w}}}}}=\,{w}_{{{{\rm{weighted}}}}}\times \frac{1}{n}{\sum}_{i=1}^{n}{\left({Y}_{i}-{\hat{Y}}_{i}\right)}^{2}$$

3 $${{{{\rm{MRE}}}}}^{{{{\rm{g}}}}}=\,\frac{1}{n}{\sum}_{i\in n}{\sum}_{j\in \left\{x,y,z,t\right\}}\frac{{\left({\partial }_{j}{Y}_{i}-{\partial }_{j}{\hat{Y}}_{i}\right)}^{2}}{{{\partial }_{j}{\hat{Y}}_{i}}^{2}}$$

Where ${Y}_{i}$ and ${\hat{Y}}_{i}$ denote the ground truth and predicted values of reservoir pressure or temperature. The 4D spatial and temporal gradients ${\partial }_{j}$ with $j\in \left\{x,y,z,t\right\}$ are computed using finite differences between adjacent grid points.

Since variations in reservoir pressure and temperature originate at well locations and exhibit the strongest gradients in near-well regions, we introduce a distance- and loss-dependent weighting function (${w}_{{{{\rm{weighted}}}}}$) to improve local accuracy in the weighted MSE terms. A Gaussian kernel is first applied to construct a smooth and physically reasonable weight map that considers both the distance to wells and the prediction errors. The resulting modified Gaussian-based^34^ weight map is given by:

4 $${w}_{{{{\rm{Gaussian}}}}}=\left(a+b\cdot {e}^{\left(-{D}^{2}/\left(2\cdot {\sigma }^{2}\right)\right)}\right)\cdot \left(1+c\cdot {\varepsilon }_{{{{\rm{re}}}}}\right)$$

Where $a$ = 0.2 ensures that all spatial locations, including far-field regions, contribute to training. $b$ = 0.8 controls the strength of near-well emphasis. $D$ denotes the normalized distance to the nearest relevant well, and specifically, the nearest injector for the temperature prediction and either the nearest injector or producer for the pressure prediction. These distances are computed from two of the model inputs, normalized distance maps (${{{{\rm{map}}}}}_{{{{\rm{inj}}}}}$ and ${{{{\rm{map}}}}}_{{{{\rm{pro}}}}}$) where a value of 0 indicates the well location and 1 to the farthest point. The Gaussian width parameter $\sigma$ = 3 controls the smoothness of the distance weighting. The considered prediction errors are defined as ${\varepsilon }_{{{{\rm{re}}}}}=\log (1+e/\bar{e})$, where $e$ is the element-wise SmoothL1 residual and $\bar{e}$ is normalized simple-wise mean. $c$ is a scaling coefficient that controls the influence of this error term.

Although the Gaussian-based map improves sensitivity in near-well areas, it remains insufficient for capturing sharp gradients in those regions. Moreover, we observe that increasing the parameter $b$ can further strengthen near-well features, but this often leads to the neglect of far-field areas and degrades overall model performance. To mitigate this trade-off, we introduce an additional near-well emphasis map ${w}_{{{{\rm{near}}}}-{{{\rm{well}}}}}$, defined as:

5 $${w}_{{{{\rm{near}}}}-{{{\rm{well}}}}}=1+d\cdot {{{{\rm{map}}}}}_{{{{\rm{near}}}}-{{{\rm{well}}}}}$$

Where $d$ = 1.5 is used for reflecting the observed greater difficulty in learning near-well pressure dynamics. The binary mask ${{{{\rm{map}}}}}_{{{{\rm{near}}}}-{{{\rm{well}}}}}$ is set to 1 if the normalized distance is less than 0.5, and 0 otherwise. For the temperature model, this mask is determined using ${{{{\rm{map}}}}}_{{{{\rm{inj}}}}}$, while for the pressure predictions, it is based on both ${{{{\rm{map}}}}}_{{{{\rm{inj}}}}}$ and ${{{{\rm{map}}}}}_{{{{\rm{pro}}}}}$. The final combined weight is then defined as:

6 $${w}_{{{{\rm{weighted}}}}}={w}_{{{{\rm{Gaussian}}}}}\cdot {w}_{{{{\rm{near}}}}-{{{\rm{well}}}}}$$

The total terms for MSEs between predicted and simulated reservoir pressure and temperature fields are then formulated as:

7 $${{{{\rm{MSE}}}}}_{{{{\rm{model}}}},{{{\rm{i}}}}}={w}_{1}\cdot {{{{\rm{MSE}}}}}_{{{{\rm{i}}}}}^{{{{\rm{s}}}}}+{w}_{2}\cdot {{{{\rm{MSE}}}}}_{{{{\rm{i}}}}}^{{{{\rm{w}}}}}\,+{w}_{3}\cdot {{{{\rm{MRE}}}}}_{{{{\rm{i}}}}}^{{{{\rm{g}}}}}$$

Where the subscript $i\in \left\{P,{T}\right\}$ denotes the pressure and temperature models, respectively. The superscripts $s$, $w$, and $g$ refer to the standard MSE, weighted MSE, and gradient based error components. The coefficients ${w}_{1}$, ${w}_{2}$, and ${w}_{3}$ are the corresponding loss weights.

The second component, ${{{{\rm{MSE}}}}}_{{{{\rm{Physics}}}}}$, comprises ${{{{\rm{MSE}}}}}_{{{{\rm{Mass}}}}}$ and ${{{{\rm{MSE}}}}}_{{{{\rm{Energy}}}}}$, which quantify the residuals of mass and energy conservation derived from the predicted states, thereby enforcing consistency with the governing conservation laws. Specifically, the predicted reservoir temperature and pressure fields are propagated through fluid property models, a radial inflow well model, and conservation formulations to evaluate deviations from mass and energy balance. The corresponding residuals are defined as:

8 $${{{{\rm{MSE}}}}}_{{{{\rm{Mass}}}}}=\frac{1}{n}{\sum}_{i=1}^{n}{\left({M}_{{{{\rm{acc}}}}}-{M}_{{{{\rm{tran}}}}}-{M}_{s}\right)}^{2}$$

9 $${{{{\rm{MSE}}}}}_{{{{\rm{E}}}}{{{\rm{nergy}}}}}=\frac{1}{n}{\sum}_{i=1}^{n}{\left({E}_{{{{\rm{acc}}}}}-{E}_{{{{\rm{conv}}}}}-{E}_{{{{\rm{cond}}}}}-{E}_{s}\right)}^{2}$$

Where ${M}_{{{{\rm{acc}}}}}$, ${M}_{{{{\rm{tran}}}}}$, and ${M}_{s}$ represent the accumulation, transport, and source/sink terms in mass conservation equations. Similarly, ${E}_{{{{\rm{acc}}}}}$, ${E}_{{{{\rm{conv}}}}}$, ${E}_{{{{\rm{cond}}}}}$, and ${E}_{s}$ are the accumulation, convective heat-transport, conductive heat-transport, and source/sink terms in energy conservation equations, respectively. The detailed formulations of these physical models are provided in Supplementary Method 2.

The third component, ${{{{\rm{RE}}}}}_{{{{\rm{task}}}}}$, measures the prediction accuracy for key task variables that are critical for assessing heat extraction performance and supporting practical geothermal operations. These variables are derived from the predicted pressure and temperature fields, where surface production temperature and thermal output are computed using a two-phase wellbore flow model (Supplementary Method 2), while injector BHP is estimated using a radial inflow model. Due to the distinct physical dimensions of these variables, a relative error formulation is adopted to normalize and evaluate their prediction accuracy. The relative error is defined as:

10 $${{{{\rm{RE}}}}}_{{{{\rm{task}}}}}={\sum}_{i}\frac{\left|{Y}_{{{{\rm{task}}}}}^{i}-{\hat{Y}}^{i}_{{{{\rm{task}}}}}\right|}{{Y}_{{{{\rm{task}}}}}^{i}}$$

Where ${Y}_{{{{\rm{task}}}}}$ and ${\hat{Y}}_{{{{\rm{task}}}}}$ are the ground truth and predicted values of task variables, and $i$ refers to three task variables, including surface production temperature, surface thermal energy output, and injector BHP.

The contributions of each component are controlled by weighting coefficients, and the complete loss function is defined as:

11 $${{{\rm{Loss}}}}={{{{\rm{MSE}}}}}_{{{{\rm{Model}}}}}+{w}_{4}\cdot {{{{\rm{MSE}}}}}_{{{{\rm{Physics}}}}}+{w}_{5}\cdot {{{{\rm{RE}}}}}_{{{{\rm{task}}}}}$$

By integrating direct prediction errors with physics-informed constraints and task-specific targets, the proposed loss function establishes a comprehensive training objective. It enables the PCNO model to learn not only from direct field values but also from the underlying physical laws and specific outputs, thereby improving generalization, predictive accuracy, and interpretability across a wide range of geothermal scenarios.

### PCNO model training procedure

Following construction of the PCNO and definition of the loss function, training is conducted using prepared input-output pairs. Two modules are built for pressure and temperature prediction. In each module, the inputs are first processed by the A-U-FNO to generate pressure or temperature fields, which are then propagated through the governing PDEs to evaluate physical consistency and compute task variables. To ensure that PCNO effectively learns all components of this workflow and follows a numerically stable optimization trajectory, training of both modules is organized into three sequential phases: (1) model-term pretraining, (2) physics-informed joint training, and (3) fine-tuning.

In Phase 1, the loss includes only ${{{{\rm{MSE}}}}}_{{{{\rm{Model}}}}}$, with a gradually decreased weight on the standard MSE (${{{{\rm{MSE}}}}}^{{{{\rm{s}}}}}$) and the gradually increase weights on the weighted MSE (${{{{\rm{MSE}}}}}^{{{{\rm{w}}}}}$) and gradient mean relative error (${{{{\rm{MRE}}}}}^{{{{\rm{g}}}}}$). Since ${{{{\rm{MSE}}}}}^{{{{\rm{w}}}}}$ and ${{{{\rm{MRE}}}}}^{{{{\rm{g}}}}}$ typically exhibit larger magnitudes at the beginning of the training, this schedule prevents them from dominating the loss and promotes stable optimization. It allows the PCNO to first learn the overall spatial distributions and temporal evolution of pressure and temperature. Once these global patterns are captured, the growing contributions of ${{{{\rm{MSE}}}}}^{{{{\rm{w}}}}}$ and$\,{{{{\rm{MRE}}}}}^{{{{\rm{g}}}}}$ guide training toward improved resolution of near-well variations and local spatio-temporal dynamics.

In Phase 2, physics-informed loss terms are incorporated, including conservation-residual losses for mass and energy and relative-error terms for key task variables. These components are introduced only after Phase 1 to avoid physical constraints dominating the objective and misguiding optimization before the model achieves sufficient accuracy in the predicted reservoir states. In preliminary tests, early inclusion of physics and task terms was found to induce unstable optimization and degraded performance, because their effectiveness depends strongly on the quality of the predicted reservoir states.

In Phase 3, a fine-tuning stage is applied. The weights of all loss terms are no longer scheduled but are fixed at constant values that are selected empirically based on the model behaviour in Phases 1 and 2. Freezing the relative contributions of each term enables more targeted refinement, further improving global accuracy and enhancing the resolution of near-well pressure and temperature variations.

Overall, Phases 1 and 2 use time-dependent weighting strategies in the composite loss, whereas Phase 3 uses fixed weights. All three phases are trained under a five-stage learning-rate schedule. The detailed training configurations, learning-rate settings, and weight schedules are summarized in Supplementary Method 4 and Supplementary Table 2.

### Embedded power estimation module for geothermal applications

To enable the estimation of geothermal power output, a power calculation module is embedded within the trained PCNO model. This module transforms the predicted thermodynamic fields into practical estimates of both thermal and electrical energy production.

The amount of electrical energy that can be generated from geothermal heat is primarily influenced by both heat extraction performance and the efficiency of surface facilities. Different power-plant technologies often exhibit different requirements on the produced fluid in order to generate electricity effectively. Based on a worldwide review of representative geothermal power plants^35^, we apply an enthalpy-dependent efficiency model to estimate electricity generation from the produced geothermal fluid. The thermal-to-electrical conversion efficiency is defined as^35^:

12 $$\eta=7.8795\cdot {{{\mathrm{ln}}}}\left(h\right)-45.651$$

Where $\eta$ is the conversion efficiency. $h$ is the average production enthalpy. In this work, $\eta$ is set to zero when temperature below 75 °C, indicating that electricity generation is not feasible with current technologies under such conditions. In addition, any negative efficiency values calculated from the production enthalpy are also set to zero to reflect the inability to generate electricity. The installed power capacity and total generated electricity amount are computed using the following expressions:

13 $${W}_{p}=\eta \cdot Q\cdot \triangle H$$

14 $${E}_{p}={\sum}_{t}^{T}{W}_{p}\cdot {H}_{a}\cdot \omega$$

Where ${W}_{p}$ (MW_e_) is the installed power capacity. $Q$ (kg s^−1^) is the total fluid production rate. $\triangle H$ is the enthalpy difference between the produced and injected fluid. ${W}_{h}=Q\cdot \triangle H$ (MW_th_) is the installed thermal capacity. ${E}_{p}$ is the total electrical energy produced. ${H}_{a}$ is the total number of hours in a year. $\omega$ is the capacity factor, reflecting the operational availability of the geothermal power plant, which is assumed to be 0.85. *T* is the total number of time steps in the operational period. Note that the temperature threshold of 75 °C and the capacity factor of 0.85 may vary between projects and regions. A sensitivity analysis of these two parameters is presented in Supplementary Fig. 10.

### Integrated economic module for financial assessment

Economic evaluation is essential for geothermal project development because it enables operators to assess the financial viability and long-term sustainability of a project. To this end, building on previous reports and existing tools, we develop an economic evaluation module integrated into the trained PCNO model to quantify the economic performance of geothermal systems across different end uses, including heating and electricity generation. The levelized cost of heat (LCOH) and the levelized cost of electricity (LCOE) are employed as the primary indicators.

This module considers capital expenditures and annual operation and maintenance (O&M) costs specific to each application and computes LCOH or LCOE based on predicted system performance from the PCNO model, using the following expression^36^:

15 $${{{\rm{LCOH}}}}\; {{{\rm{or}}}}\; {{{\rm{LCOE}}}}=\frac{{C}_{{{{\rm{cap}}}}}+{\sum}_{t=1}^{n}\frac{{C}_{{{{\rm{O\&M}}}}}}{{\left(1+r\right)}^{t}}}{{\sum}_{i=1}^{n}\frac{{E}_{t}}{{\left(1+r\right)}^{t}}}$$

Where ${C}_{{{{\rm{cap}}}}}$ is the total capital investment. ${C}_{{{{\rm{O\&M}}}}}$ is the annual O&M cost in year $t$. $r$ is the discount rate, which is assumed to be 10 %. $n$ is the project lifetime. ${E}_{t}$ is the annual energy output, either heat or electricity.

The total capital investment include expenditures for drilling and completion (${C}_{{{{\rm{well}}}}}^{{{{\rm{cap}}}}}$), resource exploration (${C}_{\exp }^{{{{\rm{cap}}}}}$), surface plant construction (${C}_{{{{\rm{pp}}}}}^{{{{\rm{cap}}}}}$), fluid distribution (${C}_{{{{\rm{fd}}}}}^{{{{\rm{cap}}}}}$), and reservoir creation (${C}_{{{{\rm{rc}}}}}^{{{{\rm{cap}}}}}$), which is expressed as:

16 $${C}_{{{{\rm{cap}}}}}={C}_{{{{\rm{well}}}}}^{{{{\rm{cap}}}}}+{C}_{\exp }^{{{{\rm{cap}}}}}+{C}_{{{{\rm{pp}}}}}^{{{{\rm{cap}}}}}+{C}_{{{{\rm{fd}}}}}^{{{{\rm{cap}}}}}+{C}_{{{{\rm{rc}}}}}^{{{{\rm{cap}}}}}$$

Drilling and completion costs are estimated based on measured well depth using empirical correlations derived from previous reports^37^. Reservoir exploration costs are calculated following the approach adopted in GETEM^38^, which expresses exploration expenses as a function of well capital costs. Investments in surface facilities are highly dependent on the intended end use. For heating systems, an empirical formula is employed to estimate capital costs^39^, while for electricity generation, a capacity-based function is used to determine the capital costs of the power plant^40^. Fluid distribution costs, associated with the surface fluid gathering system, are computed based on the installed thermal or electrical capacity^41^. In cases where reservoirs require enhanced permeability, technologies such as stimulation and existing fracture cleaning are necessary to create high-conductivity flow paths. The associated reservoir creation costs are assumed to be 30% of the drilling costs^42^. A conversion index ($F$) is used to convert the costs to 2025 USD based on the U.S. Bureau of Labor Statistics (BLS) Consumer Price Index Inflation Calculator, which is assumed to be 1.36 in this work. These cost components are expressed as:

17 $$\begin{array}{c}{C}_{{{{\rm{well}}}}}^{{{{\rm{cap}}}}}=N\cdot \left(1.72\times {10}^{-7}\cdot {{{{\rm{MD}}}}}^{2}+2.3\times {10}^{-3}\cdot {{{\rm{MD}}}}-0.62\right)\cdot F\\ {C}_{\exp }^{{{{\rm{cap}}}}}=1.12\cdot (1+0.6\cdot {C}_{{{{\rm{well}}}}}^{{{{\rm{cap}}}}})\cdot F\end{array}$$

18 $${C}_{{{{\rm{pp}}}}}^{{{{\rm{cap}}}}}=\left\{\begin{array}{cc}150\cdot {W}_{H}\cdot F; \hfill & {{{\rm{Heating}}}}\\ {W}_{P}\cdot 2000\cdot {e}^{-0.0045\cdot \left({W}_{p}-5\right)}\cdot F; & {{{\rm{Electricity}}}}\end{array}\right.\,$$

19 $${C}_{{{{\rm{fd}}}}}^{{{{\rm{cap}}}}}=50\cdot W\cdot F$$

20 $${C}_{{{{\rm{rc}}}}}^{{{{\rm{cap}}}}}=0.3\cdot {C}_{{{{\rm{well}}}}}^{{{{\rm{cap}}}}}$$

Where $N$ is the number of wells drilled. ${{{\rm{MD}}}}$ is the measured well depth. $W$ is the installed capacity.

Annual O&M costs include the operation of wellfield (${C}_{{{{\rm{well}}}}}^{{{{\rm{O\&M}}}}}$), power plant (${C}_{{{{\rm{pp}}}}}^{{{{\rm{O\&M}}}}}$), and water make-up system (${C}_{{{{\rm{wm}}}}}^{{{{\rm{O\&M}}}}}$), which are defined as:

21 $${C}_{{{{\rm{O\&M}}}}}={C}_{{{{\rm{well}}}}}^{{{{\rm{O\&M}}}}}+{C}_{{{{\rm{pp}}}}}^{{{{\rm{O\&M}}}}}+{C}_{{{{\rm{wm}}}}}^{{{{\rm{O\&M}}}}}$$

The O&M costs of the power plant are assumed to consist of 75 % labor and 1.5 % of the capital cost of the power plant^36^, with labor costs estimated using correlations from GETEM^38^. The wellfield O&M costs include the remaining 25 % of the labor cost and 1 % of the capital costs of the wells^36^. Annual expenses for make-up water are calculated based on a water rate of 0.66 $ ton^-1^^43^, while the annual injection amount ($$Q$$) representing the required for make-up volume, where the produced water is assumed to be 100% re-injected and the make-up volume is equal to the difference between the produced water volume and the required injection volume in the next operation year. These O&M components are expressed as:

22 $${C}_{{{{\rm{pp}}}}}^{{{{\rm{O\&M}}}}}=0.75\cdot F\cdot {C}_{{{{\rm{labor}}}}}+0.015\cdot {C}_{{{{\rm{pp}}}}}^{{{{\rm{cap}}}}}$$

23 $${C}_{{{{\rm{well}}}}}^{{{{\rm{O\&M}}}}}=0.25\cdot F\cdot {C}_{{{{\rm{labor}}}}}+0.01\cdot {C}_{{{{\rm{well}}}}}^{{{{\rm{cap}}}}}$$

24 $${C}_{{{{\rm{labor}}}}}^{E}=\left\{\begin{array}{c}236 \hfil{;f}{W}_{P} < 2.5{{{{\rm{MW}}}}}_{{{{\rm{e}}}}}\\ 589\cdot {ln}\left({W}_{P}\right)-304{;if}{W}_{P}\ge 2.5{{{{\rm{MW}}}}}_{{{{\rm{e}}}}}\end{array}\right.$$

25 $${C}_{{{{\rm{labor}}}}}^{H}=\left\{\begin{array}{c}236; \hfill \,{if}\,{W}_{H} < 2.5{{{{\rm{MW}}}}}_{{{{\rm{th}}}}}\\ 589\cdot {ln}\left({W}_{H}/5\right)-304{;if}{W}_{H}\ge 2.5{{{{\rm{MW}}}}}_{{{{\rm{th}}}}}\end{array}\right.$$

26 $${C}_{{{{\rm{wm}}}}}^{{{{\rm{O}}}}\&{{{\rm{M}}}}}=0.66\cdot Q\cdot F$$

By integrating these investment components with the predictive outputs from the trained PCNO model, the proposed economic evaluation module enables robust and comprehensive economic assessment of geothermal project economics. This framework supports improved decision-making by providing detailed insights into the financial performance and viability of reservoir-specific developments. Note that this module is designed as a general-purpose, screening-level techno-economic assessment tool to support comparative evaluation across a wide range of geothermal scenarios, rather than to reproduce the detailed cost structure of any specific project. Accordingly, some cost components are represented using empirical correlations and simplified assumptions adopted from publicly available reports and established tools. In addition, because the module is implemented as an external component within the PCNO framework, it remains flexible and readily customisable. Project- or region-specific parameters, such as discount rate, plant cost curves, and water price, can be modified to reflect specific contractual and regional conditions without retraining the surrogate model. The detailed information of each component in this economic module is provided in Supplementary Method 6.

## Supplementary information

Supplementary Information

Transparent Peer Review file

## Data Availability

All data generated in this study and used for analysis have been deposited in the Figshare database under accession code 10.6084/m9.figshare.31957083.

## Code availability

The full source code of the PCNO model used in this study is publicly available for download at 10.24433/CO.2608907.v1.
