# Supplementary material for: A robust physics-constrained neural operator framework for efficient geothermal resource development
Source: Nat Commun. 2026 May 15;17:6929. doi: 10.1038/s41467-026-73183-0 (PMC13389401; doi:10.1038/s41467-026-73183-0)
Supplement: Supplementary file 1 — Supplementary Information [file 41467_2026_73183_MOESM1_ESM.pdf]

Supplementary Information

A Robust Physics-Constrained Neural Operator Framework for Efficient Geothermal Resource Development

Zhenqian Xue[1], Jianfei Bi[2], Haoming Ma[3], Zhe Sun[1], Zhangxing Chen[1,4,5*]

[1] Department of Chemical & Petroleum Engineering, University of Calgary, Calgary, AB, Canada
[2] Research Institute of Petroleum Exploration and Development (RIPED), PetroChina, Beijing, China
[3] School of Energy Resource, University of Wyoming, Austin, Laramie, WY, USA
[4] Eastern Institute of Technology, Ningbo, Zhejiang, China
[5] National Key Laboratory of Petroleum Resources and Engineering, China University of Petroleum (Beijing), Beijing, China
* Corresponding author: Zhangxing Chen, zxchen@eitech.edu.cn

Supplementary Methods

Supplementary Method 1: Numerical modeling settings.

We investigate geothermal energy extraction through circulated water injection over a 20-year operational period. Numerical simulations are developed using the CMG STARS thermal simulator. A base model is first constructed following the settings of our previously geothermal reservoir models[1–3]. The target geothermal resource area is typically located at a medium to deep depth. To reduce computational cost of data generation while preserving representative physics, we extract a subdomain of 960 × 960 × dz m^3 from the base model as a representative target geothermal subsurface area, where dz represents the target reservoir thickness. The subdomain is discretized with 80 grids in each horizontal direction and 5 grids vertically, resulting in a total of 80 × 80 × 5 cells. This configuration provides sufficient spatial resolution to capture coupled fluid flow and heat transport in both horizontal and vertical directions while maintaining computational efficiency.

The numerical model adopts the following assumptions: (1) Local thermal equilibrium is assumed, that is, no temperature difference exists between the fluid and the rock at the same location. (2) Water is taken as the only flowing phase and is assumed to remain in the liquid state over the investigated temperature and pressure ranges. (3) The dominant mechanisms of fluid flow and heat transport are considered, whereas geomechanical effects and geochemical reactions are neglected in order to balance the predictive capability of the surrogate model with the computational cost and efficiency required for model training. The model incorporates temperature- and pressure-dependent thermophysical properties of water, including density, viscosity, heat capacity, and enthalpy, to accurately capture thermodynamic variations during reservoir development. The inlet Semi-analytical Model in CMG STARS is also employed to simulate pressure drop and thermal loss along the production wellbore. A schematic of the base model and the considered physical mechanisms is provided in Supplementary Figure 1.

For the boundary conditions, fluid flow is restricted at the top and bottom boundaries of the model. Boundary temperatures are prescribed based on the geothermal gradient and reservoir depth. At the top boundary, a heat-loss formulation is applied to represent heat exchange from the ground surface to the atmosphere. For the initial conditions, the initial reservoir pressure is assumed to be hydrostatic and computed from the reservoir depth together with a specified pressure gradient. The initial reservoir temperature is assumed to be in steady state and is calculated using a prescribed geothermal gradient.

These integrated settings allow the numerical model to represent not only the complex coupled processes of fluid flow and heat transfer in the reservoir but also the critical wellbore dynamics during operation, thereby enhancing the accuracy and reliability of the simulation results. For each simulation run, the initial timestep is set to 0.1 day, and the maximum timestep is limited to 10 days to ensure numerical stability and to capture injection and production transients. The simulations utilize an adaptive implicit method that dynamically switches between fully implicit and implicit pressure, explicit saturation schemes based on local conditions. Under this method, certain grid blocks are always treated using the fully implicit method, including those with porosity less than 0.01, those connected to aquifers or heaters, and those containing active wells. This strategy balances computational efficiency with numerical stability across heterogeneous reservoir conditions. The simulation output is recorded annually over the 20-year production period. Key output variables are recorded annually, including reservoir pressure and temperature, surface fluid temperature, thermal energy output, and injector bottom hole pressure.

Based on the numerical framework described above, we select eight key sensitive reservoir properties and randomly assign values for them within their representative ranges to capture the variability observed in current geothermal systems.

- Reservoir depth: The reservoir depth is defined within the range of 1,500 m to 3,500 m to represent typical geothermal fields[4].
- Target reservoir thickness: The reservoir thickness is assumed to vary between 50 m and 500 m, reflecting the typical vertical extent of productive geothermal intervals[5].
- Pressure gradient: The reservoir pressure gradient is varied from 7 to 15 MPa km^{-1} [6] to represent different system conditions. Given the assumed reservoir depth and target reservoir thickness, the corresponding initial reservoir pressure at the top of the target interval ranges from 12 to 48 MPa, which is used as an input to the PCNO model in place of the pressure gradient.
- Geothermal gradient: The geothermal gradient is assumed to range from 25 to 75 °C km^{-1} [4,7], representing typical geothermal systems that have been explored and are feasible for developing. When combined with the reservoir depth range, the resulting ranges of the initial reservoir temperature at the top of the study area is from 90 to 250 °C, which is used as an input to the PCNO model in place of the geothermal gradient.
- Permeability field: To represent subsurface heterogeneity, we generate permeability fields using the Spectral Random Field (SRF) method implemented in the GTOOLS Python library[8]. This Fourier-based technique efficiently simulates stationary Gaussian random fields over structured grids. In total, 5,000 realizations are created with log-normally distributed permeability values, accounting for variations in correlation length, variance, and mean. Permeability in the x and y directions is assumed to be isotropic, while the permeability in the vertical (z) direction is taken as one-tenth of that in the horizontal directions. The correlation length and variance both vary between 0.1 and 0.5, and permeability ranges from 0.1 to 300 mD, representing different depositional environments suitable for effective heat transfer[5]. Notice that these realizations are not intended to represent specific reservoirs but serve to test the robustness of the proposed model under various heterogeneous conditions.
- Porosity field: The porosity field is derived using an empirical relationship between porosity and permeability, reflecting the correlation observed in the previous study[9]. To incorporate spatial variability and account for natural heterogeneity, the calculated porosity values are perturbed by adding Gaussian noise with a mean of zero and a standard deviation of 0.001.
- Rock thermal properties (heat capacity & thermal conductivity): Based on previous measurements from representative geothermal formations, the rock heat capacity is set within the range of 8.5×10^4 to 3.4×10^5 J m^{-3} $°C^{-1}$, while thermal conductivity varies between 1.8×10^6 to 3.3×10^6 J m^{-1} day^{-1} $°C^{-1}$ [10].

In addition to reservoir characteristics, we define nine key operational parameters to represent a range of potential development strategies. Although individual parameter combinations may not correspond to a specific

field, all ranges are selected to satisfy realistic operational constraints and to provide representative coverage of practical geothermal design space.

- Number of wells: As most geothermal reservoirs remain in the early stages of development, wellfields typically include a small number of injectors and producers, and operational decisions are dominated by first-order well interference. This regime is therefore most relevant for early-stage screening, uncertainty quantification, and operational strategy design, which are the primary intended applications of the PCNO. From a surrogate-modelling perspective, increasing the well count rapidly expands the control space (well number, placement, and control schedules) and introduces higher-order well-well interactions that are more difficult to learn reliably. Considering both data-generation and training budgets, we restrict the numbers of injectors and producers to 1-4 each, which already covers common early-stage patterns, captures the key mechanisms governing reservoir pressure support and thermal sweep, and enables robust sampling and stable model training across a broad range of geological and operational uncertainties.
- Well location: Wells are placed near the center of the reservoir subdomain using standard geothermal layouts, including doublet, triplet, four-spot, and five-spot patterns. Their positions are varied by adjusting well spacing. In total, 100 distinct well configurations are generated. Representative diagrams of these patterns are provided in Supplementary Figure 2.
- Well depth: In geothermal developments, injectors and producers may be placed at different depths to reduce the risk of reinjected water overcooling the reservoir. Accordingly, the depths of injectors and producers are randomly sampled within the depth interval of the target reservoir.
- Well spacing: In geothermal development, larger well spacing may lead to insufficient heat extraction, while smaller spacing can increase the risk of early thermal breakthrough. Based on results from our previous sensitivity analysis, the well spacing is assumed to range from 200 m to 600 m[11,12].
- Injection rate: Similar to well spacing, the injection rate significantly influences production performance. High rates can accelerate thermal breakthrough, whereas low rates may lead to insufficient circulation and reduced heat extraction. In this work, we consider both constant-rate and time-varying injection schedules. For constant-rate control, the injection rate remains unchanged over the full 20-year production period. For time-varying control, the operation is divided into three stages: ramp-up, plateau and ramp-down. Each stage is defined by its duration, with all durations sampled between 0 and 20 years and constrained to sum to 20 years. Given an initial injection rate, the ramp-up and ramp-down stages additionally use rate-change factors to specify the magnitude of increase or decrease, where ramp-up factor is in [0, 1], ramp-down factor is in [-1, 0], while the plateau stage maintains a constant rate. As an example, suppose the initial injection rate is 20 kg s^{-1}. If the ramp-up, plateau, and ramp-down durations are set to 2, 15, and 3 years, respectively, with a ramp-up factor of 0.5 and a ramp-down factor of -0.3, the injection rate increases linearly from 20 to 45 kg s^{-1} over the first 2 years (ramp-up), remains at 45 kg s^{-1} for the next 15 years (plateau), and then decreases gradually from 40 to 15.4 kg s^{-1} over the final 3 years (ramp-down). Across all scenarios, the 20-year average injection rate ranges from 10 to 100 kg s^{-1}, representing reasonable injection strategies informed by existing geothermal projects and prior sensitivity analyses[13-15].
- Injection temperature: A water circulation operation strategy is assumed in this study, where the injection fluid temperature is primarily controlled by surface facilities[16,17]. Although current systems rarely cool the fluid to very low temperatures after heat extraction, a lower bound of 20 °C is selected to represent an ideal scenario that may become feasible with future technological advancements. The upper bound is set at 70 °C, reflecting the typical capability of existing surface systems. Higher injection temperatures are avoided, as they can significantly reduce heat recovery efficiency, particularly when the extracted energy is intended for electricity generation.

- Production pressure: The production pressure of geothermal wells is influenced by multiple surface and operational constraints, such as separator pressure, scaling management, and power-plant design. These constraints are highly site specific and typically require integrated design of the well and surface facilities. To provide a general and transferable representation of production control at the reservoir scale, in this work, production pressure is treated as an operational control variable and follows standard reservoir-evaluation practice by maintaining a pressure drawdown relative to the initial reservoir pressure to sustain circulation and enable effective heat extraction. The lower bound of the production pressure is set at 7 MPa below the initial pressure to avoid excessive pressure drawdown, which could accelerate the migration of cold injected fluid and lead to early thermal breakthrough. The upper bound is set at 1 MPa below the initial pressure, representing a stable operational condition for sustained geothermal energy extraction.

Supplementary Method 2: Governing Equations Integrated into the PCNO Framework.

To enable the PCNO model to better capture domain-specific knowledge and enhance its predictive accuracy, we incorporate governing partial differential equations (PDEs) into the model. These include mass and energy conservation equations for the reservoir, radial inflow equations for wells, thermodynamic relationships for fluid properties, and equations accounting for heat loss and temperature drop in wellbores.

Mass conservation equations: Fluid flow in geothermal reservoirs is governed by Darcy's Law. The predicted pressure fields from the PCNO model are constrained by the mass conservation equations[18], which are expressed as:

$$\frac{\partial(\rho_f \phi)}{\partial t} = -\nabla \cdot \left[\rho_f \frac{k}{\mu_f}(\nabla p + \rho_f g \nabla z)\right] + q_f \tag{1}$$

- Mass accumulation term (M_{acc}): $\frac{\partial(\rho_f \phi)}{\partial t}$
- Mass transport term (M_{tran}): $-\nabla \cdot \left[\rho_f \frac{k}{\mu_f}(\nabla p + \rho_f g \nabla z)\right]$
- Source/sink term (M_s): q_f

Where ρ_f (kg m^{-3}) is fluid density. ϕ is porosity. k (m^2) is permeability; μ_f (Pa·s) is fluid viscosity. p (Pa) is pore pressure. To achieve mass balance physics, it should follow: $M_{\text{acc}} - M_{\text{tran}} - M_s = 0$

Energy conservation equations: Thermal equilibrium transport is assumed between the solid and the fluid, meaning the temperatures of both are equal at any point in space and time. In the PCNO framework, the predicted temperature fields are constrained by energy conservation, which are expressed as[18]:

$$\frac{\partial(V_f \rho_f U_f + V_r U_r)}{\partial t} = -\nabla \cdot \left[\rho_f H_f \frac{k}{\mu_f}(\nabla p + \rho_f g \nabla z)\right] + \nabla \cdot (\lambda_{\text{eff}} \nabla T) + Q_f \tag{2}$$

- Energy accumulation term (E_{acc}): $\frac{\partial(V_f \rho_f U_f + V_r U_r)}{\partial t}$
- Heat convection term (E_{conv}): $-\nabla \cdot \left[\rho_f H_f \frac{k}{\mu_f}(\nabla p + \rho_f g \nabla z)\right]$
- Heat conduction term (E_{cond}): $\nabla \cdot (\lambda_{\text{eff}} \nabla T)$
- Source/sink term (E_s): Q_f

Where V_f and V_r (m^3) are fluid phase and rock volumes. U_f (J kg^{-1}) and U_r (J m^{-3}) are the internal energy of fluid and rock. λ_{eff} (W m^{-1} K^{-1})) is the effective thermal conductivity. These three factors are defined as[19,20]:

$$U_f = H_f - \frac{p_f}{\rho_f} \tag{3}$$

$$U_r = C_{\text{pr}}(T - T_r) \tag{4}$$

$$\lambda_{\text{eff}} = \lambda_f \cdot (\lambda_r/\lambda_f)^{0.6354} \tag{5}$$

Where H_f (J kg^{-1}) is fluid enthalpy. p_f is fluid pressure. C_{pr} (J m^{-3} °C^{-1}) is rock volumetric heat capacity. T_r (°C) is the reference temperature. λ_r and λ_f (J m^{-1} day^{-1} °C^{-1}) are thermal conductivities of the solid and fluid. To achieve energy balance physics, it should achieve: $E_{\text{acc}} - E_{\text{conv}} - E_{\text{cond}} - E_s = 0$.

Radial inflow well equations: The radial inflow well model establishes a coupling between the wellbore pressure and the average pressure of the surrounding grid block. It is employed to compute key downhole parameters, including the production flow rate and the bottom-hole pressure (BHP) of injection wells. The grid block is characterized by an effective drainage radius, and the radial flow equation is solved between this effective radius and the wellbore radius. Within the PCNO framework, this equation is integrated with mass and energy conservation laws to help the model learn the physical interactions between the wellbore and reservoir and to constrain the prediction of injector BHPs. The flow rate or BHP is calculated using the general form of the radial inflow equation[21]:

$$q = \frac{2\pi hkf_h f}{ln(r_e/r_w) + S}\frac{k_{\text{rf}}}{\mu_f}(p_{\text{wf}} - p) \tag{6}$$

Where q (kg s^{-1}) is the downhole flow rate of the fluid. h (m) is the reservoir layer thickness. k (m^2) is the absolute permeability near the wellbore. $f_h = 1$ is the layer thickness factor. $f = 1$ is the well fraction. r_e (m) is the effective block radius. r_w (m) is the wellbore radius. $S = 0$ is the skin factor. k_{rf}/μ_f is the relative mobility of the fluid. p_{wf} (Pa) is the flowing wellbore pressure. p (Pa) is the grid block pressure. The effective block radius and relative mobility of the fluid are expressed by[21]:

$$r_e = 0.249 \cdot \sqrt{(\Delta x^2 + \Delta y^2)/f\pi} \tag{7}$$

Where Δx and Δy are the grid sizes in the x and y directions, which are 10 in this work. k_{rf} is the relative permeability of the fluid.

Wellbore dynamic equations: Wellbore pressure drop and thermal loss are critical for determining the recoverable heat at the surface, which are influenced by factors such as reservoir depth and geothermal gradient and is necessary to be evaluated for different reservoir conditions. In this study, we assume consistent well properties across all cases. To capture wellbore thermal behavior during geothermal operation and to enable accurate prediction of surface production performance, we incorporate a steady-state two-phase wellbore formulation into the PCNO framework. The model is based on a drift-flux description of two-phase momentum transport and is coupled with radial heat transfer between the wellbore and the surrounding formation[22].

- Momentum transport

Fluid momentum transport in a geothermal wellbore is described through the total pressure gradient, which is decomposed into hydrostatic, frictional, and acceleration contributions[22]:

$$-\frac{dp}{dz} = g\rho_m \sin\theta + \frac{f v_m^2 \rho_m}{2d} + \rho_m v_g \frac{dv_m}{dz} \tag{8}$$

Where p is pressure. z is measured depth. g is gravitational acceleration. ρ_m is the mixture density. θ is the deviation angle from horizontal. v_m is the mixture velocity. d is the tubing inner diameter. f is the Moody friction factor, which is computed using the explicit rough-pipe correlation[23], with Reynolds number $R_{e_m} = \rho_m v_m d/\mu_m$, mixture viscosity μ_m, and roughness ε:

$$f = [4\log(\frac{\varepsilon/d}{3.7065} - \frac{5.0452}{R_{e_m}}\log[\frac{(\varepsilon/d)^{1.1098}}{2.8257} + \left(\frac{7.149}{R_{e_m}}\right)^{0.8981}]]^{-2} \tag{9}$$

The thermodynamic region in the wellbore is determined by pressure and enthalpy at each depth. The properties of the carrier are computed based on the steam quality (x), which is given by[22]:

$$x = \frac{h - h_L^{\text{sat}}(p)}{h_g^{\text{sat}}(p) - h_L^{\text{sat}}(p)} \tag{10}$$

Where h is the enthalpy. $h_L^{\text{sat}}(p)$ and $h_g^{\text{sat}}(p)$ are the saturated liquid and vapor enthalpy at pressure p. The mixture velocity and viscosity are further given by[22]:

$$v_m = v_{\text{sL}} + v_{\text{sg}} \tag{11}$$

$$\mu_m = x\mu_g + (1-x)\mu_L \tag{12}$$

Where $v_{\text{sL}} = \dot{m}(1-x)/(\rho_L A)$ and $v_{sg} = \dot{m}x/(\rho_g A)$ are the superficial velocity of liquid and gas. $\dot{m}$ is the total mass flow rate, and $A = \pi d^2/4$ is the tubing cross-sectional area. Thermophysical properties of water and steam are obtained from IAPWS-IF97. For mixture density, it is determined using the in-situ gas volume fraction (Eq. 13)[22], and the steam holdup is obtained via the drift-flux relationship (Eq. 14)[24].

$$\rho_m = \rho_g f_g + \rho_L(1-f_g) \tag{13}$$

$$f_g = \frac{v_{\text{sg}}}{C_0 v_m - v_\infty} \tag{14}$$

Where C_0 is the flow parameter, $C_0 v_m$ is the channel center mixture velocity, and v_∞ is the in-situ steam velocity as the sum of bubble-rise velocity. C_0 and v_∞ are obtained after the determination of flow pattern. Five flow regimes are considered: bubbly, slug, churn, dispersed bubbly, and annular. Flow-pattern transitions are identified using established criteria. In specific, the superficial gas velocity for bubbly-to-slug transition (v_{gb}), the minimum mixture velocity needed for dispersed bubbly (v_{ms}), and the churn-to-annular transition velocity (v_{gc}) are given by[22]:

$$v_{\text{gb}} = (0.43v_{\text{sL}} + 0.36v_{\infty b}) \tag{15}$$

$$v_{\text{ms}} = \left[\frac{0.725 + 4.15\sqrt{v_{\text{sg}}/v_m}}{2\left(\frac{f}{2d}\right)^{0.4}\left(\frac{\rho_L}{\sigma}\right)^{0.6}\left(\frac{0.4\sigma}{\rho_L g - \rho_g g}\right)^{0.5}}\right]^{1/1.2} \tag{16}$$

$$v_{\text{gc}} = 3.1[g\sigma(\rho_L - \rho_g)/{\rho_g}^2]^{1/4} \tag{17}$$

The corresponding values of C_0 and v_∞ are assigned according to the identified flow pattern[22]:

$$\begin{cases} v_{\text{sg}} < v_{\text{gb}} \rightarrow \text{bubbly}, & C_0 = 1.2 \; and \; v_\infty = v_{\infty b} \\ v_{\text{gb}} < v_{\text{sg}} < 1.08v_{\text{sL}} \; and \; v_m < v_{\text{ms}} \rightarrow \text{slug}, & C_0 = 1.2 \; and \; v_\infty = \overline{v_\infty} \\ v_{\text{sg}} < 1.08v_{\text{sL}} \; and \; v_m > v_{\text{ms}} \rightarrow \text{Dispersed bubbly}, & C_0 = 1.15 \; and \; v_\infty = v_{\infty b} \\ v_{\text{sg}} > 1.08v_{\text{sL}} \; and \; v_m > v_{\text{ms}} \rightarrow \text{Churn}, & C_0 = 1.15 \; and \; v_\infty = \overline{v_\infty} \\ v_{\text{sg}} > v_{\text{gc}} \rightarrow \text{Annular}, & C_0 = 1.0 \; and \; v_\infty = 0 \end{cases}$$

Where σ is the surface tension that is obtained from IAPWS-IF97. $v_{\infty b}$ and $v_{\infty T}$ are rise velocities of small bubbles (Eq. 18) and Taylor bubbles (Eq. 19), and $\overline{v_\infty}$ is the average rise velocity of steam bubbles (Eq. 20)[22].

$$v_{\infty b} = 1.53[g(\rho_L - \rho_g)\sigma/{\rho_L}^2]^{1/4} \tag{18}$$

$$v_{\infty T} = 0.35[gd(\rho_L - \rho_g)/\rho_L]^{1/2} \tag{19}$$

$$\overline{v_\infty} = v_{\infty b}(1 - e^{-0.1v_{\text{gb}}/(v_{\text{sg}} - v_{\text{gb}})}) + v_{\infty T}(1 - e^{-0.1v_{\text{gb}}/(v_{\text{sg}} - v_{\text{gb}})}) \tag{20}$$

- Energy transport

Energy conservation in the wellbore accounts for gravitational work, kinetic energy change, and radial heat exchange with the surrounding formation[22]:

$$\frac{dh}{dz} - g\sin\theta + v_m\frac{dv_m}{dz} = -\frac{Q(z)}{\dot{m}} \tag{21}$$

Where $Q(z)$ is the radial heat-transfer rate per unit depth between wellbore fluid and formation, which is composed of heat loss from fluid to the wellbore/formation interface (Eq. 22) and heat loss from this interface to the formation (Eq. 23). By combing these two terms, the overall heat-loss expression is computed by Eq. 24[22].

$$Q_1 = 2\pi r_{\text{to}} U_t (T_f - T_{\text{wb}}) \tag{22}$$

$$Q_2 = \frac{2\pi k_e}{T_D}(T_{\text{wb}} - T_{\text{ei}}) \tag{23}$$

$$Q = -L_R c_p \dot{m}(T_f - T_{\text{ei}}) \tag{24}$$

Where T_f is the fluid temperature inside the tubing. T_{wb} is the temperature at the cementing/formation interface. U_t is the overall heat transfer coefficient. T_{wb} is the wellbore/earth interface temperature. T_{ei} is the undisturbed earth or formation temperature at any depth. k_e is the earth thermal conductivity. T_D is the dimensionless temperature (Eq. 25), L_R relaxation length parameter (Eq. 26), and c_p is the mixture heat capacity (Eq. 27)[22].

$$T_D = \ln\left[e^{(-0.2t_D)} + (1.5 - 0.3719e^{-t_D})\right]\sqrt{t_D} \tag{25}$$

$$L_R = \frac{2\pi}{c_p \dot{m}}\left[\frac{r_{\text{to}} U_t k_e}{k_e + (r_{\text{to}} U_t T_D)}\right] \tag{26}$$

$$c_p = x c_{p,g} + (1 - x) c_{p,L} \tag{27}$$

Where $t_D = k_e t / \rho_e c_e {r_{\text{wb}}}^2$ is the dimensionless producing time. $c_{p,g}$ and $c_{p,L}$ are the saturated gas and liquid heat capacity.

Fluid property equations: Water properties vary significantly with pressure and temperature, particularly density, viscosity, and enthalpy. These parameters are essential for accurately estimating heat extraction performance. To capture these variations, we develop temperature- and pressure-dependent models for each property. These models are dynamically coupled with the mass and energy conservation equations, radial inflow well equations, and wellbore thermal dynamics throughout each time step of the model prediction. This integration enables the PCNO model to accurately capture fluid flow and heat transfer behavior within geothermal reservoirs.

- Density model[25]:

$$\rho_f = \rho_f^0 \cdot e^{[a\cdot(p - p_r) - b\cdot(T - T_r)]} \tag{28}$$

Where ρ_f^0 is the reference water density at the pressure (p_r) and temperature (T_r). p and T are current pressure and temperature. a is the fluid compressibility, which is 7×10^{-7}, and b is the thermal expansion coefficient, which is 8.8×10^{-4}.

- Viscosity model[26]:

$$\mu_f = \begin{cases} 1.787 \times 10^{-3} \cdot e^{[T\cdot(-0.033 + 0.0001962\cdot T)]}, & T < 40\ ^\circ\text{C} \\ 10^{-3} \cdot [1 + 0.015512 \cdot (T - 20)]^{-1.572}, & 40 \le T < 100\ ^\circ\text{C} \\ 2.414 \times 10^{-5} \cdot 10^{[247.8/(T + 133.15)]}, & T \ge 100\ ^\circ\text{C} \end{cases} \tag{29}$$

- Enthalpy model[27]:

$$H_f = \frac{1}{\rho_f^m}\left(H_f^0 + A\cdot\bar{T} + B\cdot\frac{\bar{T}^2}{2} + C\cdot\frac{\bar{T}^3}{3} + D\cdot\frac{\bar{T}^4}{4} - \frac{E}{\bar{T}} + F - H\right) \tag{30}$$

Where ρ_f^m is the mole density of the fluid. H_f^0 is the standard water enthalpy at 298.15 K. $\bar{T} = \frac{T}{1000}$ is the normalized temperature. A, B, C, D, E, F, and H are empirical coefficients, where A = -203.6060, B = 1523.29, C = -3196.413, D = 2474.455, E = 3.855326, F = -256.5478, H = -285.8304.

Supplementary Method 3: Physics-Constrained Neural Operator Structure.

The Physics-Constrained Neural Operator (PCNO) is a Fourier-based deep neural operator designed for modeling pressure and temperature fields in geothermal systems over a 4D space-time domain, which captures coupled dynamics that evolve across both space and time. The architecture is organized into two branches for pressure and temperature. Each branch contains a Global Parameter Fusion (GPF) component and four U-Net embedded Fourier Neural Operator layers (U-FNOs). The structure of PCNO is shown in Supplementary Figure 3.

In a forward process, the model receives spatial-temporal input variables together with a set of global control parameters, where the global parameters are first processed by the GPF module and then fused into the input

fields. The resulting inputs are then passed through a sequence of hybrid U-FNO blocks. Within each block, the FNO component performs global convolutions in the frequency domain to capture long-range correlations, whereas the U-Net component refines local features in the spatial domain, resolving small-scale heterogeneity such as stratified layers and preferential flow paths. U-Net outputs are combined with FNO features through a learnable gating mechanism to construct a joint spectral and spatial representation. At the output stage during training, combined data driven and physics informed loss terms are applied so that the predicted fields are consistent with both the training data and the governing equations, while accurately reproducing the task variables. In combination, these components enable the PCNO to provide accurate, physically consistent predictions of reservoir behaviors and task variables throughout the space and time domain. A brief summary of layers and outputs are provided in Supplementary Table 1.

- Global Parametric Fusion

In geothermal simulations, some variables are typically simplified as global parameters since these often exhibit limited spatial and temporal variability. Integrating them effectively is essential for improving model efficiency and accuracy. A conventional approach is to extend them into a full 4D spatial-temporal tensor to match the input shape. However, this significantly increases data volume and computational cost, potentially impacting model training and inference efficiency.

To address this, we define a pointwise mapping mechanism through a GPF module, which incorporates global geological and boundary parameters into the model in a more efficient way. Specifically, for a global parameter vector $g \in \mathbb{R}^{d_g}$, we apply a two-layer Artificial Neural Network (ANN) to generate a latent embedding:

$$\phi(g) = \mathrm{ANN}(g) \in \mathbb{R}^{C}$$

This embedding is then broadcasted across the spatial-temporal grid $[B, C, H, W, Z, T]$, ensuring that global context is uniformly injected into every location in the domain. The broadcasted global feature is concatenated with the lifted input channels and merged through a pointwise 1×1 convolution and normalization layer, yielding a fused representation that serves as the input to the subsequent hybrid spectral-spatial blocks.By fusing global information directly into the learned representation, this approach not only conditions the neural operator on boundary-independent physical settings but also significantly reduces memory usage and speeds up data processing during both training and deployment.

- Spectral Learning via 4D Fourier Neural Operator and 3D U-Net

The core modeling capacity of our architecture is built based on the FNO, which enables learning mappings between infinite-dimensional function spaces by performing global convolutions in the Fourier domain. We adopt the formal structure proposed by Li et al.[28], where a neural operator G_θ with L layers is defined as:

$$G_\theta \coloneqq Q \circ (W_L + K_L) \circ \ldots \circ \sigma(W_1 + K_1) \circ P$$

Where P and Q are pointwise lifting and projection operators. W is a linear transformation. σ is a nonlinear activation function. K is a Fourier integral operator defined as:

$$K(u)(\xi) = \mathcal{F}^{-1}\big(R \cdot \mathcal{F}(u)\big)(\xi)$$

Where R is the Fourier transform of a periodic function, $\mathcal{F}$ and $\mathcal{F}^{-1}$ denote the 3D fast Fourier transform (FTT) and its inverse, respectively.

In the PCNO, two independent U-FNO blocks for temperature and pressure prediction, and each block consists of four FNOs. For each branch, the input is first transformed into the frequency domain using an FFT over the three spatial dimensions and time. The frequency components are truncated to a fixed number of modes along each dimension and filtered using learnable complex-valued weights. The filtered representation is then transformed back into the spatial domain using an inverse FFT. To increase flexibility and local feature adaptation, each spectral layer is augmented with a skip-connected 1×1×1 linear convolution and a GELU activation. This structure allows the network to capture both long range dependencies and local heterogeneity,

which are important for representing pressure propagation and anisotropic heat transport in geothermal reservoirs.

Each U-FNO layer embeds a lightweight 3D U-Net inside the Fourier operator. The fused feature tensor $u^{(\ell)} \in R^{B\times C\times H\times W\times Z\times T}$ is reshaped into a batch of 3D fields by stacking the temporal dimension, processed by the U-Net encoder-decoder to extract multi-scale local features, and then reshaped back to the original spatial-temporal layout. The U-Net output is combined with the spectral and linear outputs through a learnable gating mechanism, so that the final representation at each layer is given by a weighted sum of the FNO output, the pointwise linear path, and the U-Net refinement. After incorporating the 3D U-Net refinement, each U-FNO layer updates the latent field according to:

$$u^{(\ell+1)}(x) = \sigma(\mathrm{GN}(K_\ell(u^{(\ell)})(x) + W_\ell(u^{(\ell)})(x) + \alpha U_\ell(u^{(\ell)})(x)))$$

where K_ℓ is the Fourier integral operator (spectral path), W_ℓ is the pointwise linear operator, U_ℓ denotes the 3D U-Net refinement applied on the spatial volume at each time step, $\alpha \in \mathbb{R}$ is a learnable gating coefficient that controls the contribution of the U-Net branch, $\mathrm{GN}(\cdot)$ is Group Normalization, and $\sigma(\cdot)$ is a nonlinear activation function. This spectral-spatial coupling enables the PCNO to retain global coherence while accurately capturing local flow and thermal patterns in heterogeneous geothermal reservoirs.

This coupled spectral and spatial representation allows the PCNO to maintain global coherence while accurately capturing local flow and thermal patterns in heterogeneous geothermal reservoirs.

- Physics-Informed Loss

To improve the physical consistency of the model beyond the training dataset, physics informed loss terms are added to the loss function after the model has learned the dominant data patterns.

In the physics-informed stage, the predicted pressure and temperature fields are first constrained to satisfy mass and energy conservation, which are fundamental to flow and heat transport in porous media. In specific, the predicted pressure and temperature fields are used to calculate each term in mass and energy conservation equations (Eqs. 1 and 2). The resulting conservation residuals are then used to compute the mass and energy loss terms, $\mathrm{MSE}_{\mathrm{Mass}}$ and $\mathrm{MSE}_{\mathrm{Energy}}$ (Eqs. 8-9 in the manuscript). Fluid properties are updated simultaneously using from the predicted states Eqs. 28-30.

Second, wellbore-related physics are incorporated through specialized formulations and task-level loss terms, enabling the model to link subsurface dynamics to well performance and surface heat extraction. Specifically, the predicted reservoir pressure and temperature values near wells are propagated through the radial inflow equations (Eqs. 6 and 7) and the wellbore dynamic equations (Eqs. 8-27) to compute task residuals for injector BHP, surface production temperature, and thermal output (Eq. 10 in the manuscript).

These physical constraints are enforced by evaluating discretized equation residuals directly on the model outputs. By jointly minimizing both data-driven loss and physics-based residuals, the PCNO is guided toward physically consistent representations, leading to improved extrapolation capability, enhanced stability, and greater reliability in surrogate-driven decision-making workflows.

Supplementary Method 4: Model Training Configurations.

After collecting 9,450 data samples from CMG-STARS, we allocate 7,350 for training, 1,050 for validation, and 1,050 for testing. To further improve model robustness, we apply an eight-fold cross-validation strategy to the combined training and validation set. Specifically, 8,400 samples were combined and divided into eight folds, and in each epoch, seven folds are randomly selected for training, and the remaining fold is used for validation. Validation starts from epoch 30, and during epochs 1-29 only training is performed.

PCNO training uses the AdamW optimizer, which has been widely adopted in FNO-based models for complex physical problems[28-31]. AdamW decouples weight decay from the gradient based parameter update, which typically improves generalization and numerical stability in deep neural networks. Compared to the standard Adam optimizer, AdamW provides more effective regularization by preventing the accumulation of

weight decay within the adaptive moment estimates, which is particularly important for physics-informed and operator-learning models that are prone to overfitting due to high model capacity and limited labeled data[33].

For the learning rate (LR), we adopt a five-stage schedule to balance early stability, mid-training convergence, and late-stage refinement. Over epochs 0-250 (Stages 1-4), the schedule consists of a warm-up and plateau followed by two cosine-annealing decays: (1) a linear warm-up phase that linearly increases the LR from 9×10^{-4} to 1×10^{-3} to reduce instability in early iterations; (2) a plateau phase that holds the LR at 1×10^{-3} to allow the model to form a stable representation; (3) a first cosine annealing phase that smoothly decays the LR to 1×10^{-5}, enabling a transition from coarse to finer optimization; and (4) a second cosine annealing phase that further reduces the LR to 1×10^{-6} for fine-scale parameter refinement. This design supports efficient exploration of the parameter space early in training while promoting accurate convergence at later epochs, which is important for learning spatiotemporal pressure and temperature dynamics.

Staring from epoch 250, a fine-tuning stage (stage 5) is performed using a small constant LR of 1×10^{-4}, which spans 250 to 300 epochs. This stage focus on target residual prediction errors while avoiding unstable parameter updates that can occur when the LR continues to decrease, thereby improving the fidelity of local gradients and well related responses. The loss-term weights used across training stages are summarized in Supplementary Table 2.

Supplementary Method 5: Score function for development plan optimization.

The assessment and optimization of geothermal development plans should account for both technical and economic performance. Building on the capability of the PCNO to rapidly predict technical and economic outputs across diverse development scenarios, we construct a composite scoring function that quantifies overall performance for both heat use and electricity generation by jointly accounting for technical effectiveness and economic viability.

In this score function, cumulative thermal and electrical energy output are used as technical indicators for heat-use and power-generation scenarios, respectively. The corresponding economic indicators are the levelized cost of heat (LCOH) and levelized cost of electricity (LCOE).

After computing the technical and economic indicators for all tested scenarios, each indicator is normalized and mapped to a unified, dimensionless score to enable consistent comparison. The technical indicator is normalized by min-max scaling across all scenarios and mapped to a score between 0 and s_T^{tech}, with larger values indicating better technical performance. The economic indicator is inversely normalized, because lower costs represent better performance. The technical and economic scores for scenario i are defined as:

$$s_i^{\text{tech}} = s_T^{\text{tech}} \cdot \frac{t_i - \min(t_i)}{\max(t_i) - \min(t_i)}$$

$$s_i^{\text{eco}} = s_T^{\text{eco}} \cdot \frac{\max(e_i) - e_i}{\max(e_i) - \min(e_i)}$$

Where s_T^{tech} and s_T^{eco} denote the maximum scores assigned to technical and economic performance, respectively, and satisfy $s_T^{\text{tech}} + s_T^{\text{eco}} = 10$. We set $s_T^{\text{tech}} = s_T^{\text{eco}} = 5$ in this work for illustration. Since the relative importance of technical and economic criteria may vary by region and application, these weights are adjustable. For example, s_T^{tech} can be increased and s_T^{eco} decreased when technical performance is prioritized. t_i is the technical indicator of scenario i (thermal or electrical energy outputs), and e_i is the corresponding economic indicator (LCOH or LCOE). The composite score for scenario i is then given by:

$$S_i^{\text{Total}} = s_i^{\text{tech}} + s_i^{\text{eco}}$$

Field optimization may also need to satisfy project-specific operational constraints, such as minimum production temperature, allowable temperature decline, or injection-pressure limits. The PCNO framework is also able to accommodate such constraints by integrating them into the scoring and screening process. As an illustration, we impose a production-temperature constraint for power-generation scenarios, requiring the

surface production temperature to remain above a specified lower bound. Because this threshold varies with technology and project context, we assume the minimum temperature required for power generation, $T_{\text{threshold}}$, is 75 °C in this work. If the minimum surface production temperature of scenario i over the operation period, $T_{\text{pro}}^{i,min}$, falls below this threshold, the scenario is considered infeasible for power generation, and its scores are set to zero. This condition is expressed as:

$$T_{\text{pro}}^{i,min} \leq T_{\text{threshold}}, \qquad s_i^{\text{tech}} = s_i^{\text{eco}} = S_i^{\text{Total}} = 0$$

Note that this module is implemented as an external, configurable component. The relative weights of technical and economic objectives in the scoring function, as well as optimization constraints such as acceptable production temperatures, temperature drops, or BHP limits, can be adjusted to align the optimization with project goals, technical boundaries, and financial risk profiles. This configurability enhances the practical utility of the framework.

Supplementary Method 6: Description of integrated economic module for financial assessment.

In this work, the economic module is designed as a general-purpose, screening-level techno-economic assessment tool to support comparative evaluation across a wide range of geothermal scenarios, rather than to reproduce the detailed cost structure of any specific project. Accordingly, some cost components are represented using empirical correlations and simplified assumptions adopted from publicly available reports and established tools. The objective is to provide a consistent and transparent economic proxy that can be coupled with PCNO-predicted technical performance.

The economic assessment of a geothermal project includes capital expenditures and annual operation and maintenance (O&M) costs. Capital expenditures consist of drilling and completion, resource exploration, surface plant construction, fluid distribution, and reservoir creation. Below we provide additional details and the rationale for each cost component used in Eqs. 17-26.

Drilling and completion: Geothermal well costs are calculated using an empirical depth-cost correlation from previous study[34,35]. This regression was developed using a geothermal well cost–depth dataset that includes cost records for 146 wells and modelled costs for 29 additional wells from WellCost Lite predictions. Historical costs were adjusted using cost index normalization, and the regression was refined using geothermal-specific data. The resulting relationship expresses total cost as a function of measured depth. Because it is fitted to reported total well costs, it implicitly reflects the major cost elements captured in those records, such as drilling services, casing, and cementing[34,36]. This depth-based formulation is intended to retain key cost sensitivity while remaining suitable for rapid screening studies.

Resource exploration: Exploration costs follow the formulation used in the Geothermal Electricity Technology Evaluation Model (GETEM) developed by the U.S. Department of Energy Geothermal Technologies Office[37]. In GETEM, the cost of an exploration well is represented as a fixed fraction of a standard production or injection well cost, together with an additional fixed cost for non-drilling activities (e.g., geophysical surveys and field campaigns) and an overhead multiplier to account for technical and office support. This approach is intended for early-stage screening, while actual exploration costs can vary with site maturity, exploration success rate, and the number and type of exploration wells required.

Surface plant construction: Surface facility costs depend strongly on the intended end use. For heat supply, we adopt the GEOPHORES formulation (Geothermal Energy for Production of Heat and Electricity "IR" Economically Simulated) developed by the National Renewable Energy Laboratory[35,38], in which surface plant investment is primarily parameterized as a function of installed thermal capacity. For electricity generation, we use a capacity-based function from a previous report[39]. In this formulation, the unit surface capital cost decreases from 2000 \$ kW^{-1} at 5 MW to 1000 \$ kW^{-1} at 150 MW, consistent with literature ranges for economies of scale, and an exponential function is fitted to match these anchor points.

Fluid distribution and reservoir creation: Fluid distribution costs are taken from GEOPHORES[35] and represent piping and surface gathering infrastructure connecting production wells, injection wells, and the plant.

Reservoir creation costs represent investments required for stimulation or cleaning of flow paths. Following a previous study[40], reservoir creation costs are approximated as a fixed fraction of drilling cost. This assumption serves as a screening-level proxy and can be adjusted when project-specific stimulation designs or cost data are available.

Operation and maintenance: Annual O&M costs include wellfield O&M, plant O&M, and make-up water expenses, which are derived from GETEM and GEOPHORES[35,37]. In specific, labor costs are estimated as functions of installed capacity and allocated between the plant and wellfield, and fixed fractions of plant and well capital costs are included to represent maintenance. Make-up water costs are computed from the required make-up volume. A unit water price of 0.66 USD ton^{-1} is adopted from prior studies[35,41] as a default. In practice, this parameter is highly region- and contract-dependent and can be replaced by project-specific supply agreements.

In this economic module, all cost correlations collected from different sources and base years are converted to 2025 USD using an inflation-based conversion factor. The LCOH/LCOE formulation and financial assumptions follow those specified in the main text. The module is intended for early-stage screening, comparative evaluation, and relative ranking of development options, rather than for detailed project cost estimation. Because it is incorporated as an external component within the PCNO framework, it remains flexible and readily customizable. Therefore, some key inputs (e.g., discount rate, plant cost curves, stimulation factor, and water price) can be replaced to reflect project-specific contractual and regional conditions.

Supplementary Figures

Supplementary Figure 1: Critical physical mechanisms during geothermal energy development.

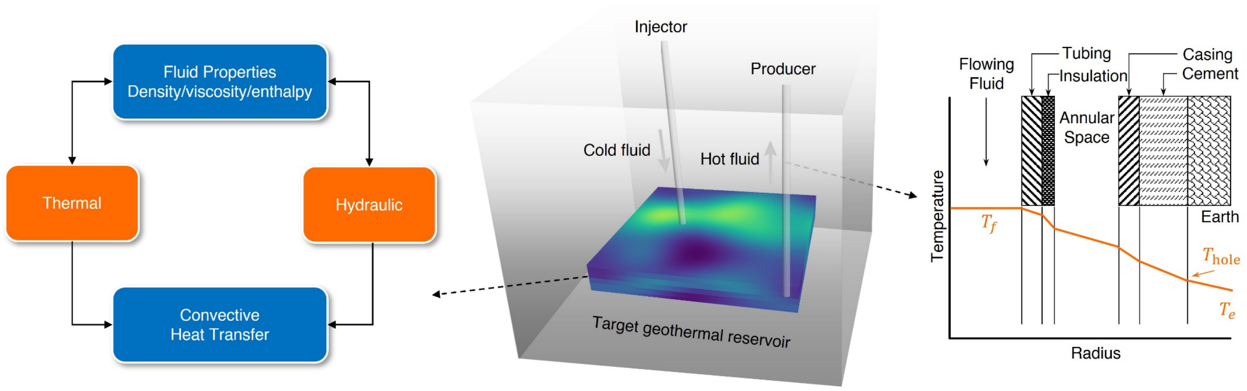

Note: T_f denotes fluid injection temperature. T_{hole} is the temperature of the well hole. T_e represents the temperature of the reservoir that is far away from the wells.

Supplementary Figure 2: Typical well patterns considered in the dataset.

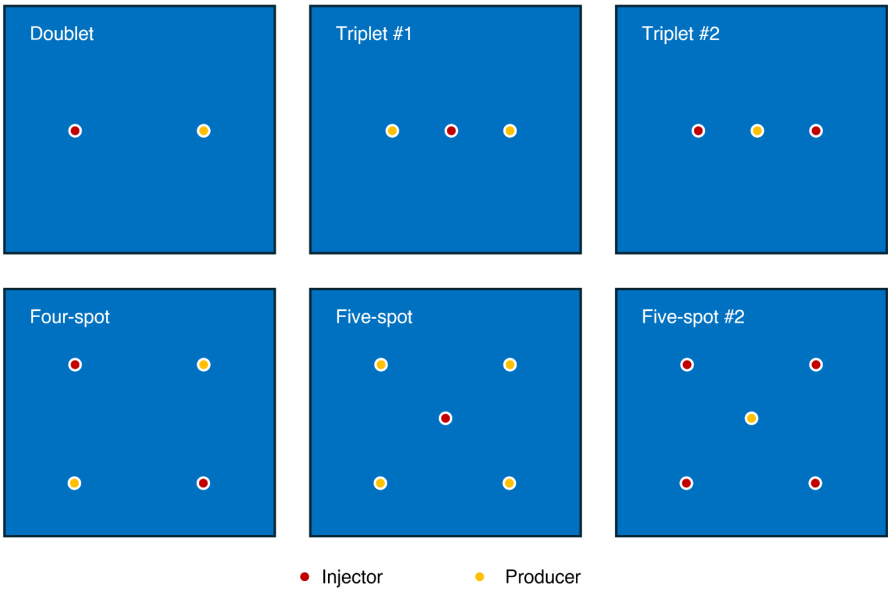

Supplementary Figure 3: PCNO model architecture.

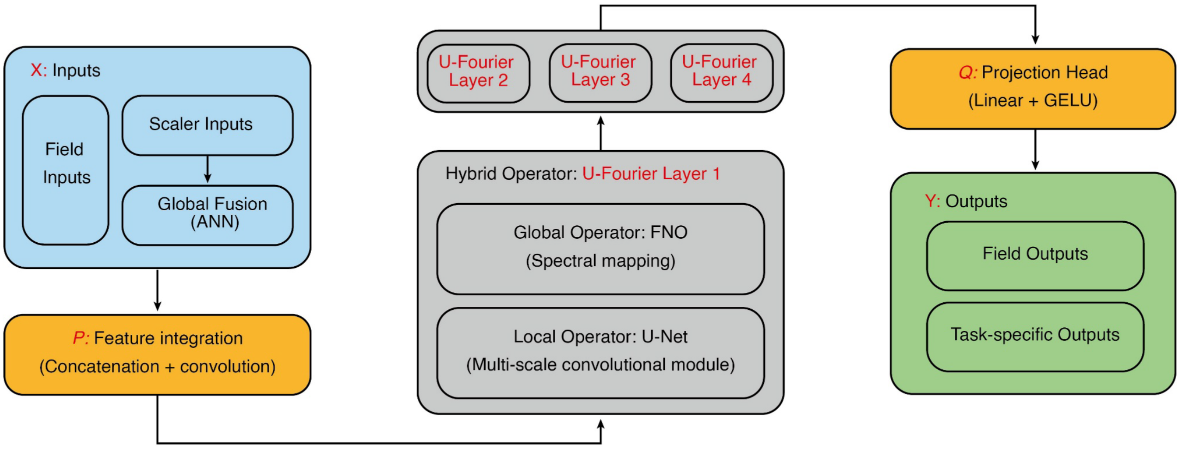

Note: X denotes the model inputs, including field variables and scaler variables. The scalar variables are first processed through an ANN-based global fusion module to extract high-level representations, which are then combined with field inputs. The fused features are further integrated through operator P, representing a fully connected neural networks used for lifting the input features into a higher-dimensional latent space.

The resulting latent features are propagated through four hybrid operator blocks (U-Fourier layers). Each hybrid operator consists of two components: a global operator based on the FNO and a local operator based on the U-Net. The global operator captures long-range dependencies by applying a Fourier transformation, followed by a learnable spectral transformation in Fourier space, and then mapping back to the physical domain using the inverse Fourier transformation. In parallel, the local operator employs a multi-scale encoder–decoder structure to extract localized spatial features through hierarchical convolution and feature aggregation. The outputs from the global and local operators are combined with a linear transformation and passed through a nonlinear activation function to produce refined feature representations.

After passing through multiple hybrid operator layers, the learned features are projected back to the output space using operator Q, which represents a fully connected projection head used for projecting the latent representation back to the target output space. The final output, denoted as Y, includes both field outputs and task-specific predictions.

Supplementary Figure 4: Model prediction performance in the 2D horizontal (XY) plane.

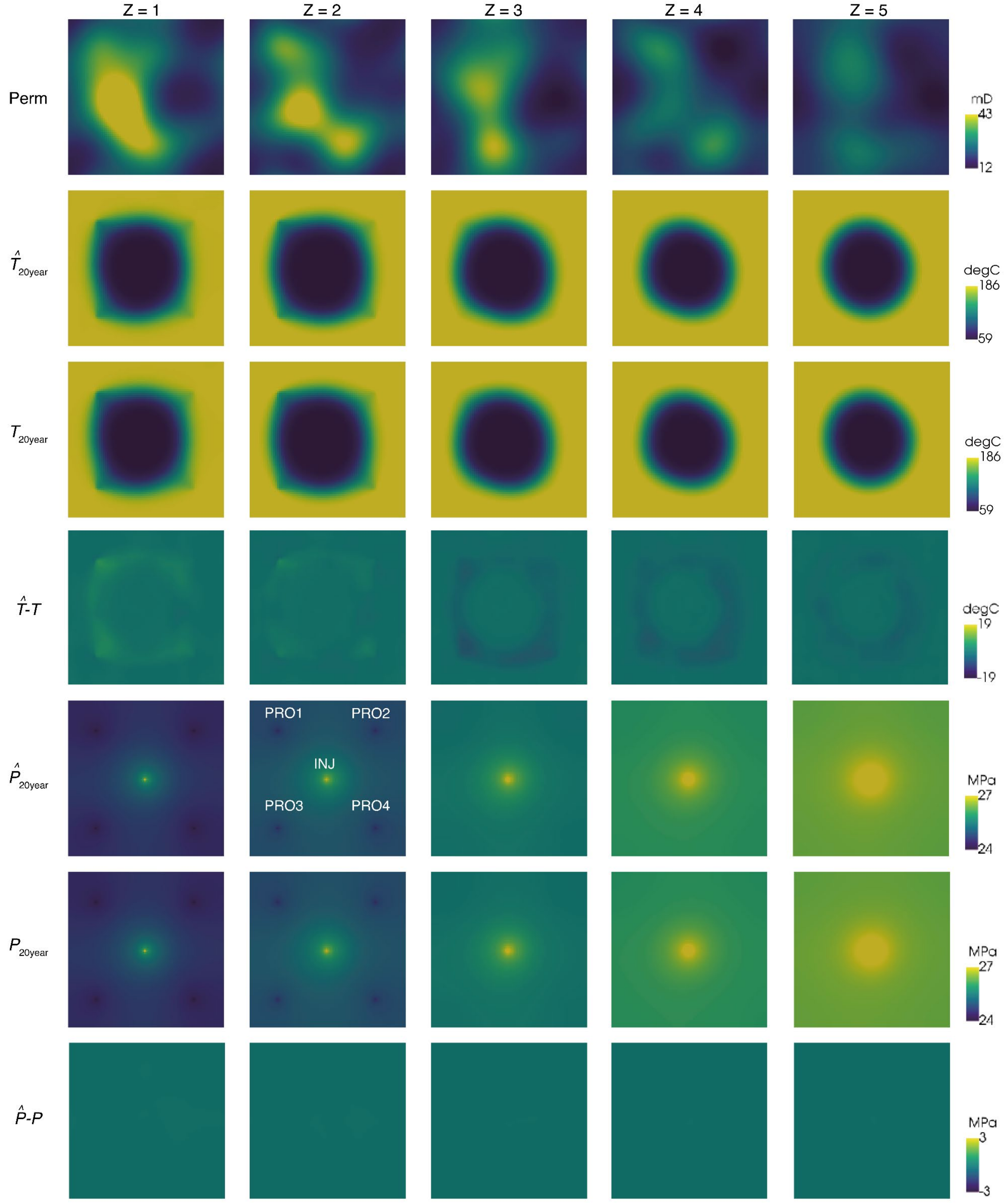

Note: Z = 1-5 denotes different reservoir layers in the vertical direction. Perm is the reservoir permeability. $\hat{T}_{20\text{year}}$ and $T_{20\text{year}}$ are the predicted and reference temperature fields at year 20, and $\hat{T} - T$ is their spatial difference. $\hat{P}_{20\text{year}}$ and $P_{20\text{year}}$ are the predicted and reference pressure fields at year 20, and $\hat{P} - P$ is their spatial difference. PRO1 - PRO4 represents four different producers, and INJ denotes the injector.

This figure compares the predicted and reference pressure and temperature fields after 20 years of operation, together with their spatial differences, across the five reservoir layers in the 2D (XY) plane. The example demonstrated corresponds to Case #3 in Figure 2 of the manuscript. In this scenario, one injection well and four production wells are drilled for heat extraction. The injector is perforated across all layers, whereas the producers are perforated only in the upper two layers. The first row displays the heterogeneous permeability distributions in each layer.

The results show that the PCNO model reproduces the long-term evolution of reservoir temperature and pressure under coupled injection and production in the horizontal plane, with only small deviations from the reference solutions. The model captures permeability-controlled flow and heat transport behaviors. For example, in layers with strong permeability contrasts (Z = 1-3 in the second row), the thermal front propagates preferentially toward the production wells through high-permeability flow paths, whereas heat transport remains spatially smoother in more laterally uniform layers (Z = 4-5).

The pressure response is also well represented, where higher pressures are correctly predicted near the injection well due to sustained fluid injection, and lower pressures are observed around production wells operating at lower bottomhole pressures (Z = 1-2 in the fifth row). In contrast, layers without producer perforations show noticeable pressure disturbance only near the injector (Z = 3-5), indicating that the model appropriately represents the spatial extent of pressure communication controlled by the well configuration.

Supplementary Figure 5: Model prediction performance in the 2D vertical (XZ) plane.

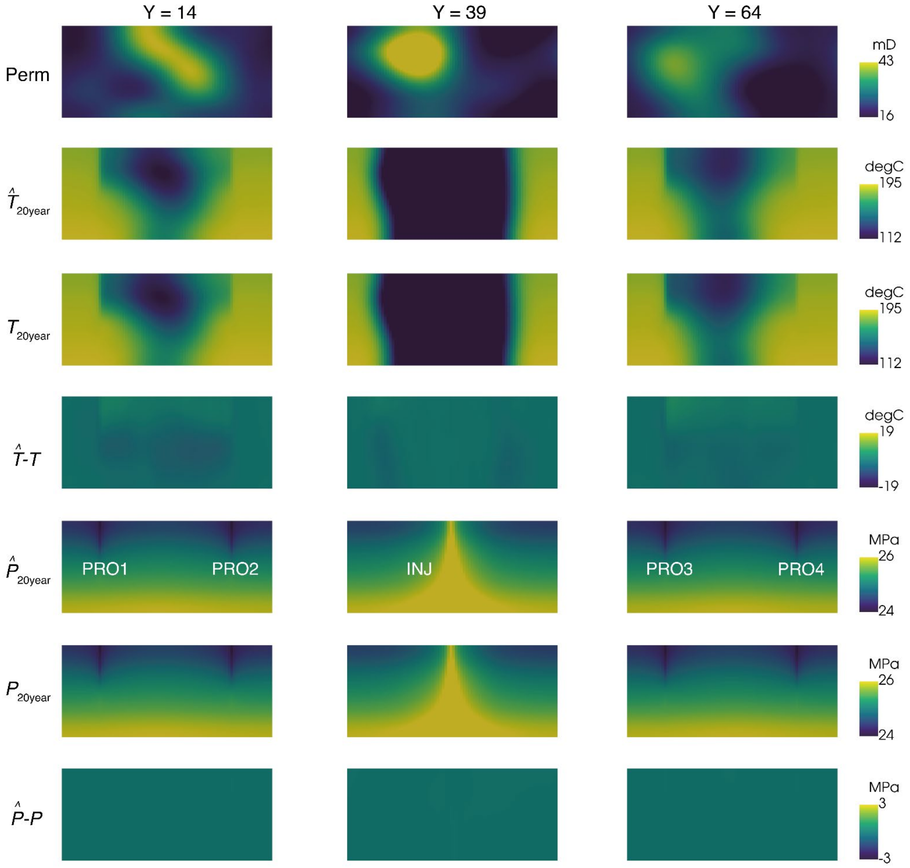

Note: Y = 14, 39, and 64 denote three vertical planes that intersect the well trajectories. Perm is the reservoir permeability. $\hat{T}_{20\text{year}}$ and $T_{20\text{year}}$ the predicted and reference temperature fields at year 20, and $\hat{T} - T$ is their spatial difference. $\hat{P}_{20\text{year}}$ and $P_{20\text{year}}$ are the predicted and reference pressure fields at year 20, and $\hat{P} - P$ is their spatial difference. PRO1 - PRO4 represents four different producers, and INJ denotes the injector.

This figure compares the predicted and reference pressure and temperature fields after 20 years of operation, together with their spatial differences, on representative 2D (XZ) planes at Y = 14, 39, and 64. The example again corresponds to Case #3 in Figure 2 of the manuscript, where one injection well is perforated across all layers and four production wells are perforated only in the upper two layers. The first row shows the heterogeneous permeability distributions in the corresponding vertical planes.

The results show that the PCNO also captures pressure and temperature evolution accurately in the vertical direction. This demonstrates that the model can represent coupled fluid flow and heat transfer under heterogeneous reservoir conditions and specific well controls. As shown in the second row, the injected cold water moves mainly through high permeability zones in the upper layers. In particular, in the plane at Y=14, the predicted cold plume remains largely within the upper high permeability layers rather than spreading into deeper high permeability zones, because there are no producer perforations in the deeper layers. This shows that the model correctly represents well-controlled flow pathways.

For pressure predictions, both the spatial distribution and magnitude of the predicted fields are physically consistent with the imposed operating conditions. In specific, higher pressures are predicted near the injector (INJ), especially in deeper layers and in zones without producer perforations, while clear pressure drawdown develops around the production wells (PRO1 and PRO2), which are operated at lower bottomhole pressures.

Supplementary Figure 6: Model prediction performance in changeable injection rate schedule.

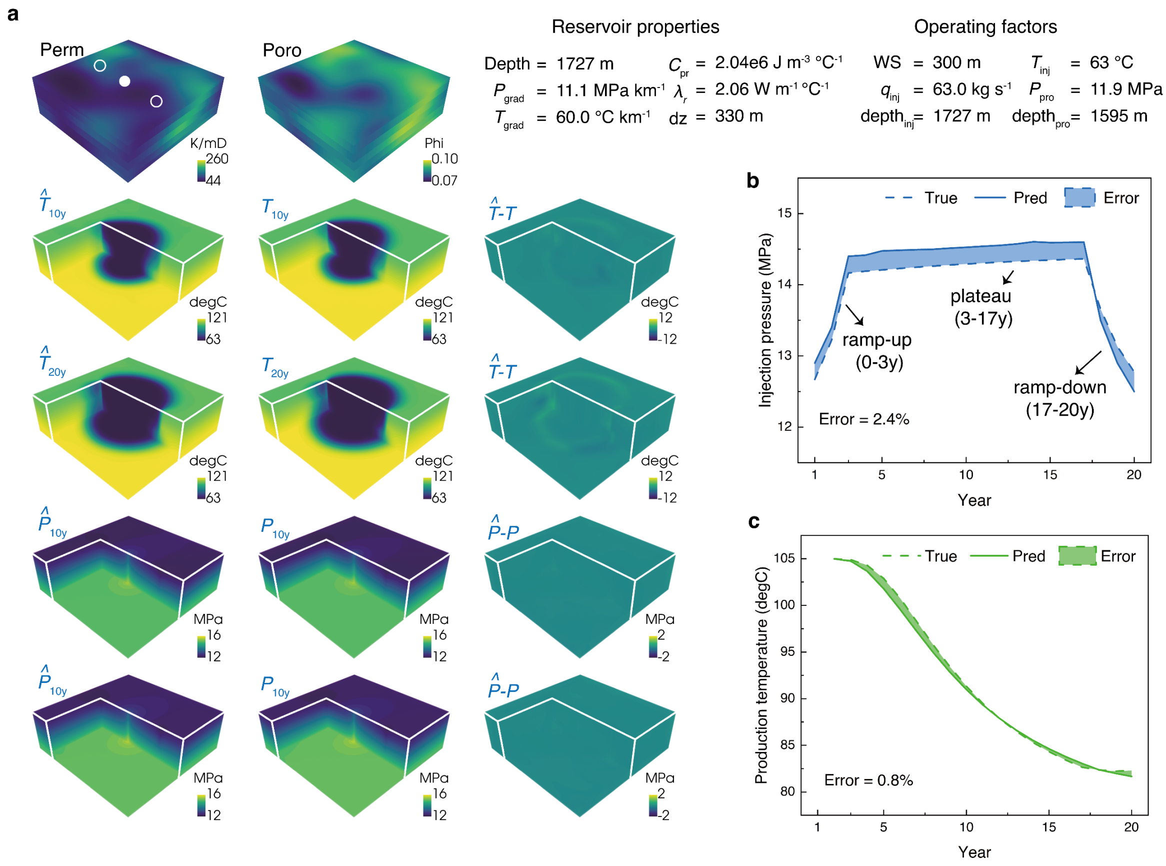

Note: **a** The predicted and reference reservoir pressure and temperature distributions at years 10 and 20, together with their differences. **b** The comparison between predicted and reference injector BHP. **c** The comparison between predicted and reference surface production temperature. Perm and Poro are reservoir permeability and porosity. $\hat{T}_{10y}$ and T_{10y} the predicted and reference temperature fields at year 10, $\hat{T}_{20y}$ and T_{20y} the predicted and reference temperature fields at year 20, and $\hat{T} - T$ is their spatial difference. $\hat{P}_{10y}$ and P_{10y} are the predicted and reference pressure fields at year 10, $\hat{P}_{20y}$ and P_{20y} are the predicted and reference pressure fields at year 20, and $\hat{P} - P$ is their spatial difference.

This figure illustrates the prediction performance of the PCNO under a time-varying injection schedule that is not included in the training dataset. For comparison, we also run a CMG-STARS simulation with the same reservoir and operating conditions. In this scenario, one injector and two producers are drilled and completed at different depths, where the injector perforated across all layers while the producers are perforated in the upper three layers. The injection rate follows a three-stage schedule. The initial injection rate is 25 kg s^{-1}, the ramp up, plateau and ramp down stages last 3, 14 and 3 years, and the rate change factors for ramp-up and ramp-down stages are 0.5 and -0.3, respectively. As a result, the injection rate gradually increases from 25 to 73.5 kg s^{-1} in the first 3 years, remains at 73.5 kg s^{-1} for the next 14 years, and then decreases to 25.2 kg s^{-1} over the final 3 years. The average injection rate over the 20-year operation is 62.8 kg s^{-1}.

The results show that the PCNO accurately predicts the evolution of reservoir pressure and temperature at different times, as demonstrated in subplot **a**. Importantly, the model also captures the impact of injection-rate changes on injector BHP (subplot **b**), where the injector BHP rises rapidly during the ramp-up stage, then increases slowly during the constant-rate plateau stage, and finally decreases during the ramp-down stage. After reproducing the reservoir evolution and well-reservoir interactions, the predicted surface production temperature shows high accuracy, which achieves an average relative error of 0.8% over the 20-year period (subplot **c**).

Supplementary Figure 7: Model prediction performance in a non-uniform reservoir structure.

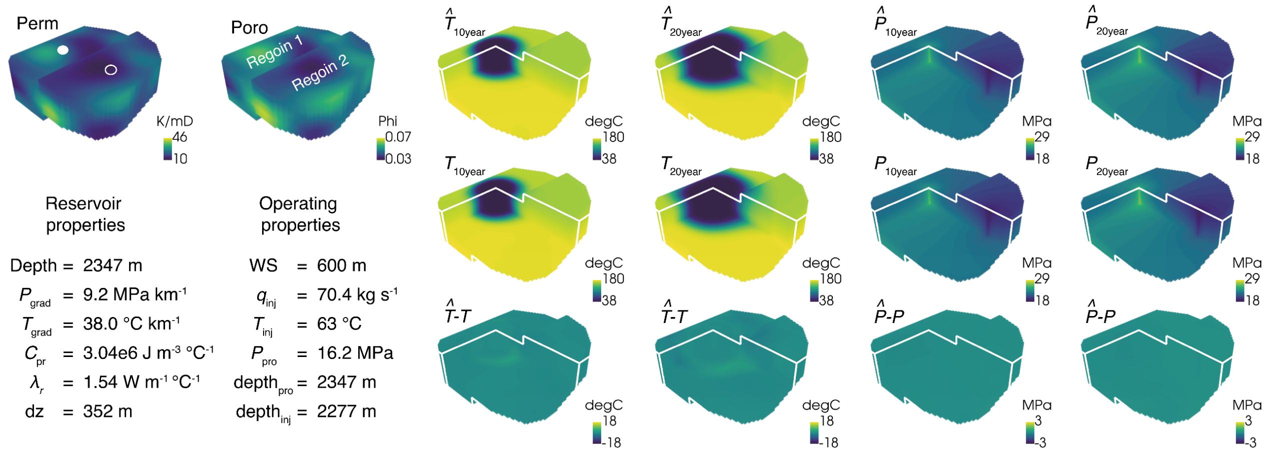

Note: Perm and Poro represent the permeability and porosity fields, respectively. $\hat{T}$ and T are the PCNO predictions and simulation results on reservoir temperature, respectively. $\hat{P}$ and P are the PCNO predictions and simulation results for reservoir pressure, respectively. The subscripts 10year and 20year mean the results at years 10 and 20. $\hat{T} - T$ and $\hat{P} - P$ are the spatial differences between the predicted and simulated temperature and pressure fields.

The PCNO model is applicable not only to geometrically uniform reservoirs but also to the reservoirs with non-uniform structural configurations. This is achieved by assigning zero values to grid cells located outside the reservoir structure in the input field variables, which effectively restricts the solution domain to the true reservoir volume and allows the model to simulate fluid flow and heat transfer within the actual reservoir boundaries.

To demonstrate this capability, we construct a case with non-uniform reservoir geometry and also contains two vertically offset regions, where Region 1 represents a deeper reservoir section and Region 2 represents a shallower section. The case is first built in CMG-STARS, which generates an irregular structural reservoir with up to 80 grid cells in the horizontal directions and up to 5 grid cells in the vertical direction, as illustrated in the Perm and Poro. For the PCNO inputs, grid cells outside the simulated reservoir structure are set to zero for all field variables listed in Table 1 of the main text.

The comparison between the PCNO predictions and CMG-STARS results shows that the PCNO accurately reproduces fluid flow and heat transfer in this non-uniform structural reservoir, capturing the evolution of pressure and temperature in both horizontal and vertical directions. Note that, subject to the constraint that the study area can be represented on an 80 × 80 × 5 grid and that reservoir and operating conditions remain within the ranges covered by the training dataset, the same input treatment can be extended to other irregular reservoir geometries, including cases with vertically offset layers.

Supplementary Figure 8: Model prediction performance in the Qiabuqia geothermal field.

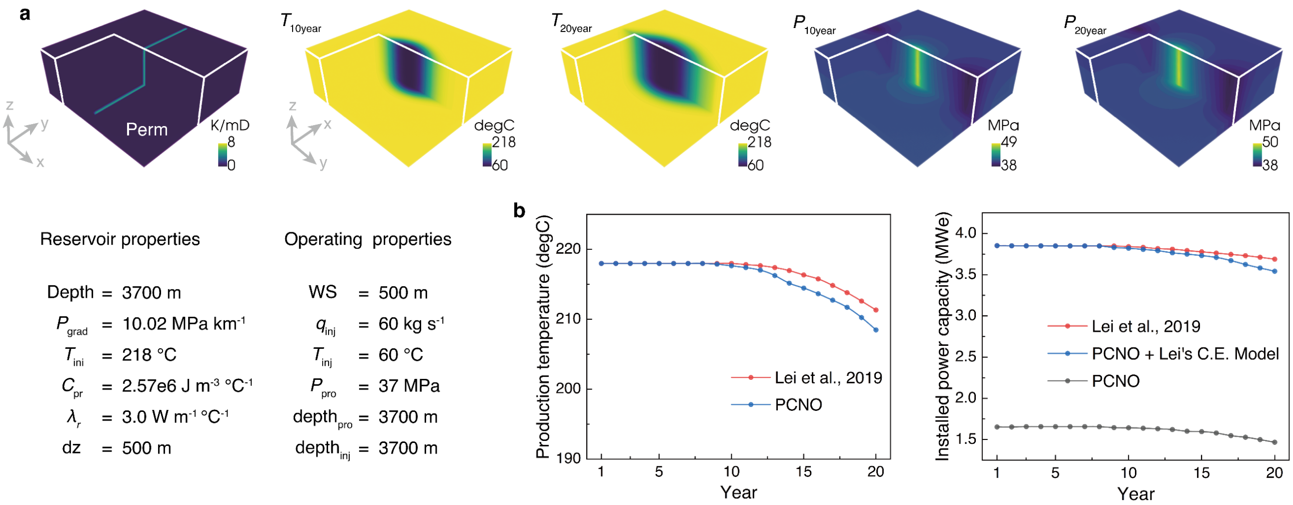

Note: **a** Permeability fields and the PCNO predicted reservoir temperature and pressure fields at years 10 and 20. **b** The comparisons of downhole production temperature and corresponding installed power capacity between the PCNO and the results of Lei et al. Note that permeability map is rotated by 90 degrees in the horizontal direction relative to the predicted reservoir fields to make the permeability pattern easier to be demonstrated.

The PCNO is designed to generalize beyond the specific realizations used for training and to be applicable to reservoirs and operating conditions that fall within the training ranges. To provide an example of this capability under realistic conditions, we construct a test case based on the Qiabuqia enhanced geothermal system (EGS) and use the PCNO to predict its heat extraction performance. Reservoir and operating conditions for this case are adopted from Lei et al.[15], whose simulation results have been widely used as a reference for this field site. A detailed description of this field can be found in their work[15].

In Lei's study, one injector and two producers were considered. Their fracturing simulations suggested the potential creation of five vertical planner engineered fractures with a height of about 102 m, a fracture half-length of 587 m, a supporting fracture half-length of 483 m and a fracture conductivity of 108 mD·m. Lei et al. approximated the stimulated region by assigning a constant permeability of 100 mD in a target zone to represent the effect of these five fractures, while retaining a permeability of 0.26 mD outside this zone.

In our test case, we adopt a related but slightly different representation of the stimulated region that is more consistent with the PCNO grid and parameterization. The reservoir and operating conditions shown in this figure are consistent with their work. Differently, instead of assigning a uniform permeability of 100 mD across the entire target zone, we assign a permeability of 9 mD to the grid cells that connect the wells and retain the matrix permeability of 0.26 mD in the remaining cells. Under the default PCNO grid resolution and the assigned reservoir thickness, this configuration is selected to approximate the effective flow enhancement associated with five vertical planar fractures, characterized by a fracture height of about 100 m, a fracture half-length of 500 m, and a fracture conductivity of 108 mD·m. The effectiveness of this type of permeability-based fracture simplification has been validated in previous studies of the Utah FORGE geothermal project[42]. Similar permeability-multiplier treatments to represent simplified planar fractures along well-connecting grids have also been used in previous EGS or oil and gas studies when fracture properties are uncertain[42-44].

Under this representation, the PCNO predictions show concentration of flow and heat transfer within the high-permeability zone and reproduce the main features of the pressure and temperature evolution in both the horizontal and vertical directions. For this field case, the operating conditions follow one of the scenarios in Lei et al., but detailed reference fields for pressure and temperature under this schedule are not available. We therefore focus on two key outputs for comparison, including downhole production temperature and installed power capacity. Note that Lei et al. did not include wellbore dynamics and evaluated heat extraction

performance using downhole production temperature. For consistency, we adopt the same metric and compute the corresponding installed power capacity.

The results (subplot **b**) show that the PCNO reproduces the downhole production temperatures with small differences and a similar trend to the results reported by Lei's result, with an initial stable period followed by a decline. The remaining differences are mainly attributable to the different permeability representations of the stimulated region. For installed power capacity, Lei et al. used an empirical conversion-efficiency correlation, $\eta = 0.45 \times (1 - T_{\text{inj}}/T_{\text{pro}})$, whereas the PCNO adopts an enthalpy-based efficiency. When Lei's efficiency formula is applied to the PCNO predictions, the estimated power capacities are close to those reported by Lei et al., with small variances that are mainly driven by the differences in predicted production temperature. When the enthalpy-based efficiency is used, the PCNO still shows a similar time trend but with lower power values. This is because the efficiency given by the enthalpy-based formulation is lower than the empirical correlation used by Lei et al.

Supplementary Figure 9: Computational speed-up.

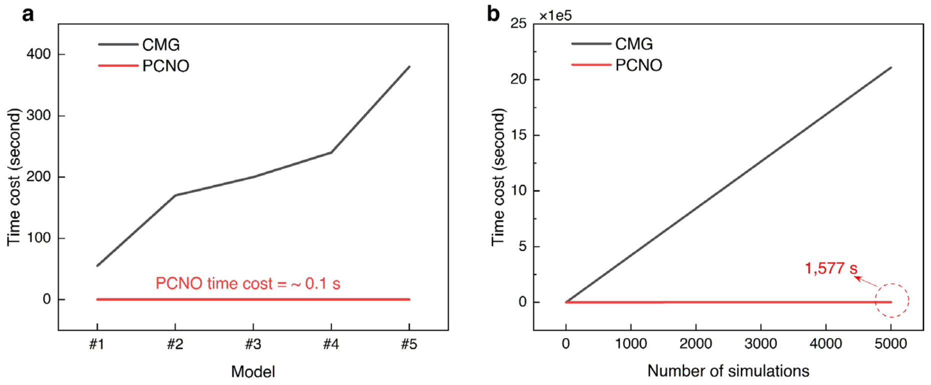

Note: **a** The prediction time of CMG-STARS and PCNO for models with different levels of complexity. **b** The prediction time costs of CMG-STARS and PCNO for different numbers of simulation cases.

The predictive efficiency of PCNO can be attributed primarily to its independence from case complexity and its suitability for large-scale ensemble evaluations. To demonstrate this, we compare the prediction time of CMG STARS and the PCNO across models of increasing complexity and for different ensemble sizes. This benchmark reports prediction time only and does not include data preparation for both tools. The PCNO is evaluated on an NVIDIA A100 PCLe GPU, whereas CMG STARS is evaluated on an AMD Ryzen 9 5950X CPU.

To assess the impact of model complexity, five cases are constructed. Model #1 is the simplest configuration, with homogeneous permeability and porosity, a grid of 40 × 40 × 5, one injector and one producer, and no wellbore dynamics. Model #2 increases the grid resolution to 80 × 80 × 5. Model #3 uses the same grid as Model #2 but includes heterogeneous permeability and porosity. Model #4 uses the same heterogeneous reservoir as Model #3 but increases the well number to four, including two injectors and two producers. Model #5 is based on Model #4 and further incorporates two-phase wellbore dynamics.

The results show that CMG-STARS runtime increases with model complexity, mainly because more time steps and solver iterations are required as the grid is refined, heterogeneity is introduced, and additional wells and wellbore physics are included. In contrast, the PCNO runtime remains nearly unchanged across all five models at approximately 0.1 s per case, consistent with the fact that inference cost depends primarily on the network architecture rather than on case settings such as grid resolution or the number of wells. This difference becomes increasingly important for large-scale tasks that require hundreds or thousands of evaluations. As shown in subplot **b**, for an ensemble of 5,000 cases, the PCNO requires about 1,577 seconds, while CMG STARS requires more than 2,000,000 seconds. Note that the average PCNO time per case for the 1,050 test cases lies between the values of single-case and 5,000-case. This is primarily due to data-loading overhead. A single case has a much smaller data size than an ensemble of 1,050 cases, so reading data into memory is faster, while the larger dataset for 5,000 cases leads to higher I/O and memory-transfer costs and slightly increases the average time per case.

We also measure the PCNO prediction time on the AMD Ryzen 9 5950X CPU. In this setting, the average runtime is approximately 63 s per case, which is still much faster than CMG STARS, especially for complex models and large ensembles. An additional advantage of the PCNO is that it is naturally suited to modern GPU hardware. By contrast, most widely used reservoir simulators are mainly designed for CPU based computation and not optimized for GPU execution. Although some commercial tools (e.g., Intersect) provide GPU support, reported runtime reductions relative to CPU-based runs are often limited[46]. Overall, once trained and validated, the PCNO provides substantial computational acceleration, especially for large ensemble evaluations on modern GPUs.

Supplementary Figure 10: Sensitivity analysis of technical and economic parameters.

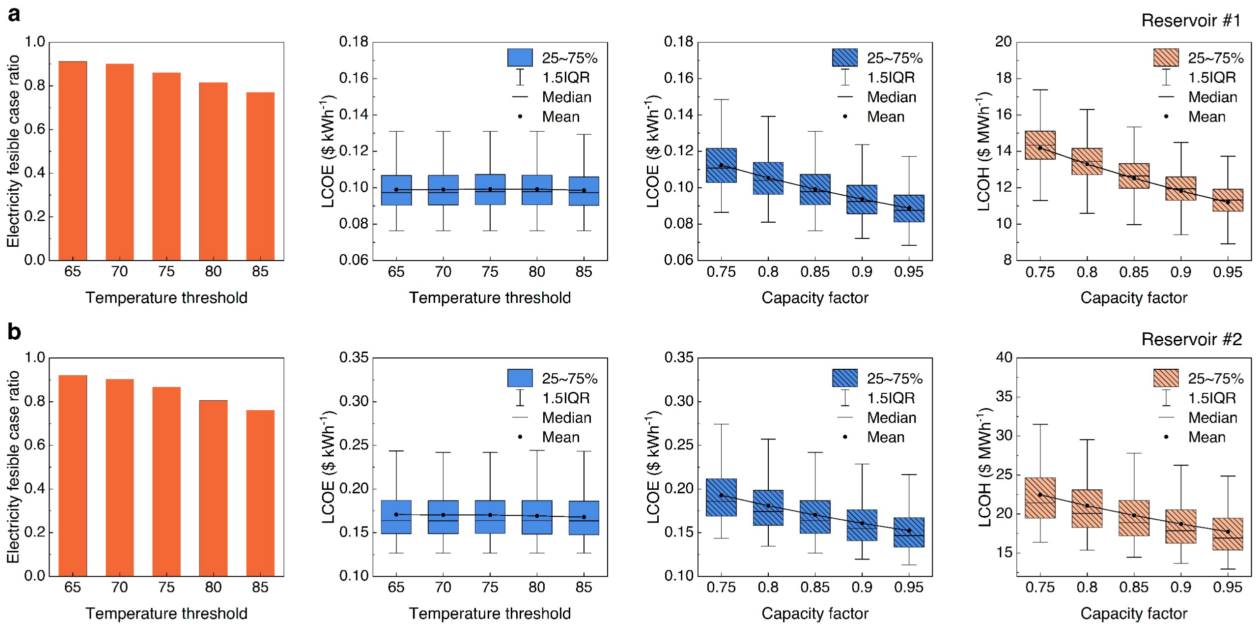

Note: **a** The sensitivity analysis results of temperature threshold and capacity factor for Reservoir #1. **b** The sensitivity analysis results of temperature threshold and capacity factor for Reservoir #2.

Because the economic module is externally incorporated into the PCNO framework, it can be customized by adjusting key uncertain inputs to reflect project- or region-specific requirements. As an illustration, we perform a sensitivity analysis on two external parameters that commonly vary across projects, including the minimum temperature required for electricity generation and the capacity factor. Specifically, we evaluate the impact of these two parameters on the technical and economic performance of the tested scenarios for Reservoirs #1 and #2 (Figure 4 in the manuscript). The temperature threshold is varied from 65 to 85 °C, and the capacity factor is varied from 0.75 to 0.95.

The results show that increasing the temperature threshold reduces the fraction of cases that remain feasible for electricity generation but has limited impact on the LCOE among the feasible cases under the tested operating schemes. For example, in Reservoir #1, the fraction of electricity-feasible cases decreases from about 90% to 78% as the threshold increases from 65 to 85 °C, whereas the average LCOE of feasible cases remains close to 0.1 $ kWh^{-1} across all thresholds. Reservoir #2 exhibits the same overall trend. These results indicate that the temperature threshold primarily acts as a feasibility filter, affecting how many scenarios qualify for power generation, rather than materially changing the cost performance of those that remain feasible. In contrast, the capacity factor has a direct effect on both LCOE and LCOH, with higher capacity factors reducing levelized costs because more useful energy is generated over the project lifetime, which reduces the unit costs.

This flexibility enables the PCNO to quantify the impact of uncertain external assumptions and to support more robust screening of reservoir potential under alternative settings, such as temperature-threshold and capacity-factor. More broadly, because key inputs in the economic module can be adjusted or replaced to reflect project- and region-specific requirements, the same PCNO workflow can be adapted to different economic and operational contexts without retraining the surrogate model.

Supplementary Tables

Supplementary Table 1: PCNO model parameters.

Layer	Operation	Pressure module	Temperature module
		Output shape	
Field variables input	-	(1, 80, 80, 5, 21, 14)	(1, 80, 80, 5, 21, 14)
Lifting layer	Linear / Permute	(1, 64, 80, 80, 5, 21)	(1, 56, 80, 80, 5, 21)
Scaler variables input	-	(1, 4)	(1, 4)
Global fusion	ANN	(1, 64, 80, 80, 5, 21)	(1, 56, 80, 80, 5, 21)
Final input	Concat / Conv1D	(1, 64, 80, 80, 5, 21)	(1, 56, 80, 80, 5, 21)
U-Net	U-Net3D	(1, 64, 80, 80, 5, 21)	(1, 56, 80, 80, 5, 21)
FNO Layers 1-4	Fourier4D / Conv1D / GELU	(1, 64, 80, 80, 5, 21)	(1, 56, 80, 80, 5, 21)
Projection 1	Permute / Linear / GELU	(1, 80, 80, 5, 21, 256)	(1, 80, 80, 5, 21, 224)
Projection 2	Linear	(1, 80, 80, 5, 21, 1)	(1, 80, 80, 5, 21, 1)
Output	Squeeze	(1, 80, 80, 5, 21)	(1, 80, 80, 5, 21)

Note: Due to computational resource limitations, a batch size of 1 is adopted during training. At each iteration, a single sample is randomly drawn from the dataset to form a mini-batch. The Linear refers to a fully connected layer that lifts low-dimensional input features into a higher-dimensional latent space for better feature representation, or projects them back to scalar outputs. The Permute operation reorders tensor dimensions to comply with convolutional layer requirements. Scalar input variables are processed through an ANN and broadcast to the full spatial-temporal domain via Global fusion.

The Concat operation combines spatial field features and broadcasted scalar features along the channel dimension, after which a Conv1D layer is applied over the flattened spatial-temporal domain to perform learned channel fusion. A lightweight 3D U-Net is subsequently employed to enhance local and multi-scale spatial feature extraction, operating on the spatial dimensions while preserving the temporal resolution.

The core representation learning is performed by four stacked Fourier4D layers, each coupled with a Conv1D layer and nonlinear activation (GELU), enabling efficient modeling of long-range spatial-temporal dependencies. Finally, two projection layers map the latent features back to scalar physical quantities, and the Squeeze operation removes singleton dimensions to produce outputs consistent with the pressure and temperature field formats.

Supplementary Table 2: Detailed settings of the wights in loss functions during training.

Training phase	Epoch	$\mathrm{MSE^s}$	$\mathrm{MSE^w}$	$\mathrm{MRE^g}$	$\mathrm{MSE_{Physics}}$	$\mathrm{RE_{task}}$	LR stage
		w_1	w_2	w_3	w_4	w_5	
Phase 1	[0, 5)	1.0	0	0	0	0	Stages 1-4
	[5, 20)	1.0	$0.9\times S_w$	0	0	0	
	[20, 35)	0.9	$0.9\times S_w$	$0.3\times S_g$	0	0	
	[35, 50)	0.9	$1.0\times S_w$	$0.5\times S_g$	0	0	
Phase 2	[50, 65)	0.8	$1.0\times S_w$	$0.7\times S_g$	0.1	0	
	[65, 80)	0.8	$1.1\times S_w$	$0.9\times S_g$	0.2	0	
	[80, 95)	0.7	$1.1\times S_w$	$1.1\times S_g$	0.4	0.1	
	[95, 110)	0.7	$1.3\times S_w$	$1.3\times S_g$	0.6	0.2	
	[110, 250)	0.6	$1.5\times S_w$	$1.5\times S_g$	0.8	0.3	
Phase 3	[250, 300]	0.8	0.6	0.4	0.5	0.5	Stage 5

Note: During training Phases 1 and 2, the learning rates follow the first four stages of the learning rate schedule, whereas Phase 3 uses the last-stage fine-tuning schedule. A detailed description of the learning rate stages is provided in Supplementary Method 4. The quantities $S_w = \mathrm{MSE^s}/\mathrm{MSE^w}$ and $S_g = \mathrm{MSE^s}/\mathrm{RE^g}$ are scaling factors applied to the weighted MSE term ($\mathrm{MSE^w}$) and the mean gradient relative error term (MRE^g), respectively. These scalers are used to keep the standard MSE term ($\mathrm{MSE^s}$), $\mathrm{MSE^w}$, and $\mathrm{MRE^g}$ at comparable magnitudes in the total loss and help prevent any single component from dominating the optimization and biasing the training. This is particularly important because $\mathrm{MRE^g}$ often exhibits a larger magnitude than the other terms. The weighting strategy in Phases 1 and 2 is determined empirically by examining loss trajectories from several trial runs over the first 50 epochs and is finalized once a stable, rapidly decreasing loss curve is obtained. The Phase 3 strategy is then designed based on the behavior observed in the first two phases and the resulting model performance.

Under this weighting strategy, Phase 1 is designed to allow the PCNO to first learn the dominant patterns in the data. At the beginning of Phases 1, the loss is dominated by $\mathrm{MSE^s}$, which encourages the model to capture the overall spatial distributions and temporal evolution of reservoir pressure and temperature. As the contributions of $\mathrm{MSE^w}$ and $\mathrm{MRE^g}$ gradually increase, the training progressively emphasizes near-well variations and local temporal dynamics. Phase 2 further constrains the training by incorporating $\mathrm{MSE_{Physics}}$ and $\mathrm{RE_{task}}$ into the loss function, guiding the model toward physically consistent solutions and improved task-variable prediction. Phase 3 focuses on targeted refinement by manually adjusting the penalties of individual terms based on performance after Phases 1 and 2. In our experiments, the model already achieves high accuracy near the wells but shows residual instability in global fields and in the temporal trends of pressure and temperature. To address this, we increase the contributions of $\mathrm{MSE^s}$ and $\mathrm{MRE^g}$ and reduce that of $\mathrm{MSE^w}$ during Phase 3, which improves global consistency while preserving local accuracy.

Supplementary Table 3: Model performance comparison in training and testing.

Model	Model structure	Training (s/epoch)	Total epoch	Prediction relative error (%)			Prediction time (s)
				Pressure	Temperature	Task variables	
CMG-STARS	-			-	-	-	442,800
FNO	FNO	594	68	15.68	14.90	20.29	80
P-FNO	FNO + PDEs	720	112	8.34	8.21	10.32	120
A-FNO	FNO + GPF	340	67	15.57	14.92	20.42	60
U-FNO	FNO + U-Net	1,040	135	10.33	9.87	12.28	170
PCNO	FNO + U-Net + GPF + PDEs	1,470	300	1.93	1.58	1.70	310

Note: To demonstrate the advantages of this architecture, we compare the PCNO against a commercial simulator (CMG-STARS) and four baseline surrogate models. FNO denotes the model constructed using only the FNO architecture. P-FNO represents the model combining the FNO architecture with governing PDE constraints. A-FNO is the model that integrates a GPF module with FNO. U-FNO is the model that combining the U-Net and FNO modules. P-U-FNO is the model combining U-Net and FNO modules with PDE constraints. PCNO is the proposed model that combines GPF, U-Net, and FNO modules, together with physics-based constraints.

All surrogate models are trained using the same data split, optimizer settings, and early-stopping criterion. Training is terminated when the validation loss fails to improve by at least 1×10^{-4} for 10 consecutive epochs, and the checkpoint with the lowest validation loss is used to report all test results. All FNO-based models are trained on seven NVIDIA A100 PCIe GPUs and tested on a single NVIDIA A100 PCIe GPU. The prediction time of CMG-STARS is measured on an AMD Ryzen 9 5950X CPU. For the FNO-based models, the reported training and prediction times include loading the dataset into memory, reflecting the runtime from memory loading to completion of model inference. The preprocessing step required to convert raw data into the format used for PCNO training and testing is not included, since it is performed once prior to training and typically takes from seconds to minutes depending on the size of the data samples. The post-processing time required to convert outputs into task-specific formats is also excluded, as it is application-dependent and follows the same convention used when reporting runtimes for traditional numerical simulations. For CMG-STARS, the reported time cost includes the simulation period and model initialization between runs, but excludes data preprocessing, post-processing, and the effort to construct the numerical model, which also follows standard practice for reporting simulator runtimes.

The baseline comparisons clarify the effects of individual modules (U-Net, PDE, and GPF) on prediction accuracy and computational cost. From the results, the base FNO is the most efficient in training and inference but produces the largest test errors. Its validation loss stops improving after 68 training epochs, indicating that the model cannot further reduce the loss with continued training. This suggests that the base FNO has limited capacity to learn the high-dimensional mapping from inputs to multiple outputs and to represent the coupled dynamics of complex geothermal systems.

By adding physics-based PDE constraints, P-FNO improves prediction accuracy by about 1.9-fold relative to FNO, while requiring longer training and inference time. This is because the PDE terms provide an additional training signal that guides the model toward physically consistent solutions, supporting more stable optimization and better generalization. As a result, P-FNO continues to benefit from training over more epochs and reaches its best performance at 112 epochs. The additional cost arises mainly from computing PDE residuals and spatial-temporal derivatives at each iteration, which increases the cost of loss evaluation and backpropagation.

By integrating the U-Net, U-FNO improves prediction accuracy by about 1.5-fold compared to FNO. This gain arises primarily because the U-Net enhances the ability to capture fine-scale variations, particularly near wells and other localized features, allowing the model to benefit from a longer optimization trajectory and reach

its best performance after 135 epochs. However, this integration also substantially increases training time due to the more complex network architecture, resulting in roughly a 2.1-fold longer training time than FNO.

When comparing the effects of the physical constraints and the U-Net, the former has a stronger positive impact on both prediction accuracy and efficiency. The U-FNO remains purely data-driven and therefore still struggles to fully infer and represent the governing physics of multi-physics geothermal systems, requiring substantial training effort and potentially failing to capture all relevant mechanisms. In contrast, P-FNO uses the same network architecture as FNO and only augments the loss function with PDE residual terms. Therefore, the additional computational cost is mainly associated with evaluating the physics-based loss rather than propagating through a deeper network, leading to a more favorable trade-off between accuracy and efficiency.

The GPF module improves prediction efficiency, showing about a 1.3-fold increase in training speed while maintaining comparable prediction accuracy (FNO vs. A-FNO). This benefit is mainly attributed to faster data loading and initialization during both training and testing. In testing, the inclusion of the GPF module reduces the data volume of 1,050 samples from around 59 GB to 42 GB, resulting in lowers memory usage and input costs. Although some differences in relative errors are observed between FNO and A-FNO, they are mainly attributed to the stochastic nature of neural network training. Based on this finding, we do not separately compare three-module variants such as FNO + U-Net + GPF, FNO + PDEs + GPF, and FNO + U-Net + PDEs, since the GPF module primarily affects efficiency rather than predictive performance.

By jointly integrating physics-based PDE constraints, the U-Net module, and the GPF module into the FNO architecture, the resulting PCNO achieves an average 7.0-fold improvement in prediction accuracy relative to these baseline models, despite the increased training time associated with the more complex architecture and loss function. For machine learning models, although the training stage may require more time, it is performed offline and only once, and this one-time cost is quickly offset because the trained surrogate can replace thousands of simulations under the tested settings and can be reused for rapid predictions. For example, compared with CMG-STARS, the PCNO maintains strong predictive capability while delivering more than a 1,400-fold improvement in computational efficiency for predicting 1,050 cases.

To provide an approximate comparison of energy use, we estimate power consumption using the official thermal design power (TDP) of these two devices, with 300 W for the NVIDIA A100 PCIe GPU and 105 W for the AMD Ryzen 9 5950X CPU. Under these assumptions, predicting 1,050 cases with the PCNO consumes around 0.022 kWh, whereas running the corresponding CMG-STARS simulations consumes about 12.9 kWh. Even after accounting for the one-time energy cost of training the PCNO, this additional consumption is rapidly offset once the surrogate is applied to large ensembles, because each subsequent evaluation requires only a small fraction of the energy needed for a full numerical simulation and remains valid for the reservoirs whose conditions fall within the ranges of the training data. Moreover, based on available data, previously published surrogate models for subsurface carbon storage report training times of roughly 1,800-8,000 s $epoch^{-1}$ [46,47], so the total training-phase energy consumption of the PCNO is expected to be comparable to or lower than that of existing deep-learning surrogates, while delivering greater savings in inference-phase energy use relative to traditional reservoir simulators.

Supplementary Table 4: Parameter settings in recoverable geothermal energy potential assessments.

Parameter	Reservoir #1	Reservoir #2	Unit
Number of injectors	[1, 4]	[1, 4]	-
Number of producers	[1, 4]	[1, 4]	-
Well location	Randomly placed	Randomly placed	-
Well spacing	[200, 600]	[200, 600]	m
Average injection rate	[10, 100]	[10, 100]	Kg m^{-3}
Injection temperature	[20, 70]	[20, 70]	℃
Production pressure	[7, 15]	[27, 33]	MPa

Supplementary Reference

1. Xue, Z. *et al.* Integrated technological and economic feasibility comparisons of enhanced geothermal systems associated with carbon storage. *Appl. Energy* **359**, 122757 (2024).
2. Xue, Z., Ma, H., Sun, Z., Lu, C. & Chen, Z. Technical analysis of a novel economically mixed CO2-Water enhanced geothermal system. *J. Cleaner Prod.* **448**, 141749 (2024).
3. Xue, Z. *et al.* Thermo-economic optimization of an enhanced geothermal system (EGS) based on machine learning and differential evolution algorithms. *Fuel* **340**, 127569 (2023).
4. Barbier, E. Geothermal energy technology and current status: an overview. *Renewable and Sustainable Energy Reviews* **6**, 3–65 (2002).
5. Toth, A. & Bobok, E. What Is Geothermal Energy? in *Flow and Heat Transfer in Geothermal Systems* 1–19 (Elsevier, 2017). doi:10.1016/B978-0-12-800277-3.00001-3.
6. Donaldson, I. G., Grant, M. A. & Bixley, P. F. Nonstatic Reservoirs: The Natural State of the Geothermal Reservoir. *Journal of Petroleum Technology* **35**, 189–194 (1983).
7. Duggal, R. *et al.* A comprehensive review of energy extraction from low-temperature geothermal resources in hydrocarbon fields. *Renewable Sustainable Energy Rev.* **154**, 111865 (2022).
8. Heße, F., Prykhodko, V., Schlüter, S. & Attinger, S. Generating random fields with a truncated power-law variogram: A comparison of several numerical methods. *Environmental Modelling & Software* **55**, 32–48 (2014).
9. Pape, H., Clauser, C. & Iffland, J. Variation of Permeability with Porosity in Sandstone Diagenesis Interpreted with a Fractal Pore Space Model.
10. Wu, H., Liu, Y., Yang, M., Zhang, J. & Zhang, B. Effect of temperature-dependent rock thermal conductivity and specific heat capacity on heat recovery in an enhanced geothermal system. *Rock Mechanics Bulletin* **2**, 100045 (2023).
11. Xue, Z., Zhang, K., Zhang, C., Ma, H. & Chen, Z. Comparative data-driven enhanced geothermal systems forecasting models: A case study of Qiabuqia field in China. *Energy* **280**, 128255 (2023).
12. Xue, Z. Machine learning based techno-economic assessment and optimization of an enhanced geothermal system. *Machine Learning* **2024**, 06–21 (2024).
13. Bedre, M. G. & Anderson, B. J. Sensitivity Analysis of Low-Temperature Geothermal Reservoirs: Effect of Reservoir Parameters on the Direct Use of Geothermal Energy.
14. Snyder, D. M., Beckers, K. F., Young, K. R. & Johnston, B. Analysis of Geothermal Reservoir and Well Operational Conditions using Monthly Production Reports from Nevada and California.
15. Lei, Z. *et al.* Exploratory research into the enhanced geothermal system power generation project: The Qiabuqia geothermal field, Northwest China. *Renewable Energy* **139**, 52–70 (2019).
16. Farzanehkhameneh, P., Soltani, M., Moradi Kashkooli, F. & Ziabasharhagh, M. Optimization and energy-economic assessment of a geothermal heat pump system. *Renewable Sustainable Energy Rev.* **133**, 110282 (2020).
17. Zarrouk, S. J. & Moon, H. Efficiency of geothermal power plants: A worldwide review. *Geothermics* **51**, 142–153 (2014).
18. Xue, Z. *et al.* Exploring the role of fracture networks in enhanced geothermal systems: Insights from integrated thermal-hydraulic-mechanical-chemical and wellbore dynamics simulations. *Renewable and Sustainable Energy Reviews* **215**, 115636 (2025).
19. Phase Enthalpies. *CMG Manual.*
20. Anand, J., Somerton, W. H. & Gomaa, E. Predicting Thermal Conductivities of Formations From Other Known Properties. *Society of Petroleum Engineers Journal* **13**, 267–273 (1973).
21. Radial Inflow Well Model. *CMG Manual.*
22. Hasan, A. R. & Kabir, C. S. Modeling two-phase fluid and heat flows in geothermal wells. *Journal of Petroleum Science and Engineering* **71**, 77–86 (2010).

23. Chen, N. H. An Explicit Equation for Friction Factor in Pipe. *Ind. Eng. Chem. Fund.* **18**, 296–297 (1979).
24. Hasan, A. R. A Basic Approach to Wellbore Two-Phase Flow Modeling.
25. Sandler, S. I. *Chemical, Biochemical, and Engineering Thermodynamics*. (John Wiley & Sons, 2017).
26. Holzbecher, E. O. *Modeling Density-Driven Flow in Porous Media: Principles, Numerics, Software*. (Springer Science & Business Media, 2012).
27. Chase, M. W. NIST–JANAF Thermochemical Tables. *J. Phys. Chem. Ref. Data* 1–1951 (1998).
28. Li, Z. *et al.* Fourier Neural Operator for Parametric Partial Differential Equations. Preprint at https://doi.org/10.48550/arXiv.2010.08895 (2021).
29. Kalimuthu, M., Holzmüller, D. & Niepert, M. LOGLO-FNO: Efficient Learning of Local and Global Features in Fourier Neural Operators. Preprint at https://doi.org/10.48550/arXiv.2504.04260 (2025).
30. Ahn, D. *et al.* Lightweight Fourier Neural Operator for Time-Dependent Partial Differential Equations.
31. Khodakarami, S., Oommen, V., Bora, A. & Karniadakis, G. E. Mitigating spectral bias in neural operators via high-frequency scaling for physical systems. *Neural Networks* **193**, 108027 (2026).
32. Wen, G. *et al.* Real-time high-resolution CO2 geological storage prediction using nested fourier neural operators. *Energy Environ. Sci.* **16**, 1732–1741 (2023).
33. Zhou, P., Xie, X., Lin, Z. & Yan, S. Towards Understanding Convergence and Generalization of AdamW. *IEEE Transactions on Pattern Analysis and Machine Intelligence* **46**, 6486–6493 (2024).
34. Lukawski, M. Z. *et al.* Cost analysis of oil, gas, and geothermal well drilling. *Journal of Petroleum Science and Engineering* **118**, 1–14 (2014).
35. Beckers, K. J. H. F. Low-temperature geothermal energy: Systems modeling, reservoir simulation, and economic analysis. (Cornell University, United States -- New York, 2016).
36. *The Future of Geothermal Energy*. (Idaho National Laboratory, Idaho Falls, 2006).
37. Hanson, S. C. *Geothermal Electricity Technology Evaluation Model (GETEM) Individual Case Files and Summary Spreadsheet (GETEM Version Spring 2013)*. https://www.osti.gov/biblio/1148822 (2013) doi:10.15121/1148822.
38. Beckers, K. F., Kolker, A., Pauling, H., McTigue, J. D. & Kesseli, D. Evaluating the feasibility of geothermal deep direct-use in the United States. *Energy Convers. Manage.* **243**, 114335 (2021).
39. Sanyal, S. K. Cost of Geothermal Power and Factors that Affect It. in (Proceedings World Geothermal Congress, 2005).
40. Xie, J. & Wang, J. Compatibility investigation and techno-economic performance optimization of whole geothermal power generation system. *Appl. Energy* **328**, 120165 (2022).
41. Beckers, K. F., Lukawski, M. Z., Anderson, B. J., Moore, M. C. & Tester, J. W. Levelized costs of electricity and direct-use heat from Enhanced Geothermal Systems. *J. Renewable Sustainable Energy* **6**, 013141 (2014).
42. Piyush Kumar Kumawat *et al.* Simplified Fracture Model for Numerical Simulation of EGS: A Utah FORGE Case Study. in *50th Workshop on Geothermal Reservoir Engineering* (California, United States, 2024).
43. Llanos, E. M., Zarrouk, S. J. & Hogarth, R. A. Numerical model of the Habanero geothermal reservoir, Australia. *Geothermics* **53**, 308–319 (2015).
44. Cherry, J. Optimization Strategies for Shale Gas Asset Development.
45. Park, J. & Janova, C. Stimulated Reservoir Volume Characterization and Optimum Lateral Well Spacing Study of Two-Well Pad: Midland Basin Case Study. *Geofluids* **2020**, 1–18 (2020).
46. Wen, G., Li, Z., Azizzadenesheli, K., Anandkumar, A. & Benson, S. M. U-FNO—An enhanced Fourier neural operator-based deep-learning model for multiphase flow. *Advances in Water Resources* **163**, 104180 (2022).
47. Wen, G. *et al.* Real-time high-resolution CO_2 geological storage prediction using nested Fourier neural operators. *Energy Environ. Sci.* **16**, 1732–1741 (2023).
