# Supplementary material for: A robust physics-constrained neural operator framework for efficient geothermal resource development
Source: Nat Commun. 2026 May 15;17:6929. doi: 10.1038/s41467-026-73183-0 (PMC13389401; doi:10.1038/s41467-026-73183-0)
Supplement: Supplementary file 2 — Transparent Peer Review file [file 41467_2026_73183_MOESM2_ESM.pdf]

nature portfolio

Peer Review File

A Robust Physics-Constrained Neural Operator Framework for Efficient Geothermal Resource Development

Corresponding Author: Professor zhangxing chen

This file contains all reviewer reports in order by version, followed by all author rebuttals in order by version.

Version 0:

Reviewer comments:

Reviewer #1

(Remarks to the Author)
General comments

The authors should be congratulated for an interesting, well-written paper.

However, in my opinion they overstate the paper's impact and their work does not demonstrate the broad generalizability they claim. Their work focuses on a geothermal system that has the following characteristics:
- No natural convection or fluid movement. Allowing assumed initial temperature and pressure distributions.
- No phases changes (no boiling). Allowing single phase treatment of the fluid.
- Horizontal flow only. Therefore, no buoyancy effects or vertical flows.
- Limited types of geological heterogeneity with no faults and generally medium to high permeability (50 – 300 mD).
- Simple wellbore dynamics without multiple feedzones, or phase changes.
- Simple production and reinjection strategies.

These assumptions may be valid for low-temperature geothermal systems in certain cases, but they significantly restrict the applicability of the approach to a broad range of geothermal systems. Low-temperature, single-phase geothermal systems that are dominated by horizontal flows are important to consider in geothermal energy, but they are not representative of the broad range of systems.

It is possible that the author's new framework could be applied more broadly but they have not demonstrated that in this work.

I recommend that the authors carefully rewrite the article to accurately reflect the limited types of geothermal systems that they are studying and discuss the impact of their work considering the contribution of those types of systems to the broader field of geothermal energy.

Specific comments
Line 31 & 37 – I could not find the information:
"Global geothermal 30 resources are estimated to be 100 to 1,000 times greater than those of fossil fuels."
in the Barbier reference given. Regardless these types of estimates usually include the energy potential of EGS and AGS. The comment about barriers on line 36 is really referring to conventional geothermal. The barriers for EGS and AGS are associated with drilling and fracturing technology rather than resource uncertainty. It's misleading to indicate that by providing better tools for assessing reservoir potential and performance the potential from EGS and AGS would be unlocked. I agree that better tools for assessing reservoir potential and performance are very important. But the authors should be more careful about linking the potential.

Line 117 - "common simplification in geothermal reservoir modeling" – These types of simplifications are only usually made during initial stored heat calculations. Generally, it is the non-uniformity, heterogeneity, faulting, geological offsets etc which necessitate numerical modelling. By simplifying these away the need for numerical modelling in the first place is reduced.

Line 211 - "In summary, the PCNO model exhibits strong predictive accuracy and generalization capability. Its ability to

deliver real-time forecasts for both subsurface reservoir and well behavior and surface-level production metrics enables a comprehensive understanding of geothermal system dynamics." This is a misleading statement. The authors have tested a very limited range of geothermal conditions and therefore the method has only been shown to enable a comprehensive understanding of that very limited range. Also, the range of conditions considered are amongst the simplest geothermal conditions.

Line 236 - "enable large-scale ensemble modeling and probabilistic assessments that were previously impractical". This is not accurate any longer. Large-scale ensemble modeling and probabilistic assessments are being carried out in geothermal. eg:
Wang, Y., Voskov, D., Daniilidis, A., Khait, M., Saeid, S., & Bruhn, D. (2023). Uncertainty quantification in a heterogeneous fluvial sandstone reservoir using GPU-based Monte Carlo simulation. Geothermics, 114, 102773.

Line 303 - "However, existing workflows typically rely on separate tools for economic evaluation and 303 field optimization, resulting in significant time demands and manual effort." This is broadly true however some authors have already made progress on coupling the physical simulations with techno-economic assessments. Again see:
Wang, Y., Voskov, D., Daniilidis, A., Khait, M., Saeid, S., & Bruhn, D. (2023). Uncertainty quantification in a heterogeneous fluvial sandstone reservoir using GPU-based Monte Carlo simulation. Geothermics, 114, 102773.

Line 324 - "By jointly considering technical performance, economic cost, and operational constraints, it strengthens decision-making and accelerates the planning and deployment of geothermal energy projects." This is true but the details of the techno-economic modelling are important. The description in the Integrated economic evaluation module for financial assessment section is fairly brief. Are drilling depths always constant? What wellbore sizes are considered? How are rig mobilisation costs calculated? Are all of these costs considered to be constant over time? Therefore, assuming all the wells are drilled at the start of the project. What about the off-taker agreements e.g. Power purchase agreement?

Line 351- "Once trained and validated, the model can handle a wide range of input conditions that are critical for geothermal systems and eliminates the need for labor-intensive model construction typically required in traditional workflows." In this paper the authors have trained the model using a very simple type of geothermal system with very simple strategies for utilisation. How do the authors propose to achieve this for realisitic complex systems? Running thousands of realistically complex reservoir models to train the model prior to use will be extremely computationally expensive.

Line 358 - "In contrast, traditional simulation workflows often require dozens to hundreds of hours due to complex model configurations and iterative solution steps." This is true in general but for the simplified reservoir and production strategies investigated the computation and setup costs could be much lower.

Line 366 - "It is also important to note that the computational cost of conventional simulations increases with model complexity and solver settings, whereas the prediction time of the PCNO remains effectively independent of case complexity." How has this been demonstrated? This is a fundamental issue with the work.

Line 381 - "A key highlight of this framework is its broad generalizability." How has this been demonstrated? Having random permeability and porosity distributions within a two-dimensional domain does not demonstrate broad generalizability regardless of how "realistic" the Spectral Random Field generation method is.

Line 390 - "Our model addresses this limitation by using spatial and temporal encodings that define well settings in a generalizable format." This is also overstated. While the authors may have advanced this approach from simple well configurations, the configurations considered cannot be called complex.

Line 585 - "Power plants such as dry steam, flash steam, and binary cycle systems exhibit different requirements for the temperature of the produced fluid in order to generate electricity effectively." This comment seems to indicate the authors considered various types of geothermal systems. However, all the work presented only describes single-phase, non-convecting geothermal systems.

Supplementary Information
Line 23 - "Given that geothermal energy extraction predominantly involves horizontal fluid flow and heat transfer, the model applies 80 grids in each horizontal direction and a single grid in the vertical direction, resulting in a total of 80 × 80 × 1 cells" - This is not true in general. Vertical fluid flow is very important in convecting geothermal system (which account for the majority of systems used for electricity generation).

Line 39 - "which means abnormal geological settings such as faults, overpressured zones, or vapor-dominated reservoirs are not considered." Faults and overpressured zone are not abnormal geological settings but are in fact encountered in many if not most convecting geothermal systems. Vapor-dominated systems are rare, but I would not consider them abnormal. Complex 2-phase flow found in vapor-dominated systems is also encountered in liquid-dominated systems during production.

Line 52 - "permeability ranges from 50 to 300 mD" - This would be considered medium to very high permeability for convecting geothermal systems.

Line 69 - "As most geothermal reservoirs remain in the early stages of development, initial operation typically involves a limited number of wells. Accordingly, the number of injection and production wells is assumed to range from 1 to 4 each". It

is true that geothermal developments start with a limited number of wells. However, production scale systems often have tens or hundreds of wells. Carrying out a resource assessment assuming only a few wells will not accurately stress systems.

Line 90 - "The production pressure is primarily determined by the initial reservoir pressure and is typically maintained at a lower value to promote fluid flow toward the production wells." Again, this is only true in the case of low temperature geothermal projects. Production pressure in higher temperature projects is determined by many factors including separation pressure, temperature for scale formation, and electricity generation technology.

Line 97 - The governing equations that have been used are for single phase fluid (or an equivalent mixture version) that cannot capture the correct two-phase fluid dynamics. Similarly, the wellbore dynamics equation is a very simplified representation of the complex governing equations for wellbore flow.

John O'Sullivan

(Remarks on code availability)

Reviewer #2

(Remarks to the Author)
I co-reviewed this manuscript with one of the reviewers who provided the listed reports. This is part of the Nature Communications initiative to facilitate training in peer review and to provide appropriate recognition for Early Career Researchers who co-review manuscripts.

(Remarks on code availability)
The authors have not provided a dedicated README file with installation or execution instructions; however, the code structure is intuitive and well-organized, which made it straightforward to run. The scripts executed successfully with only a minor warning related to NumPy version compatibility:

/code/PCNO_Ele_Eco_Module.py:212: DeprecationWarning: Conversion of an array with ndim > 0 to a scalar is deprecated, and will error in future. Ensure you extract a single element from your array before performing this operation. (Deprecated NumPy 1.25.)

This warning does not affect the current functionality or reproducibility of results. Overall, the code appears to be a usable and reproducible resource for the community, though adding a concise README file with environment setup and execution steps would further improve accessibility.

Reviewer #3

(Remarks to the Author)
This study demonstrated the effectiveness of physics-constrained neural operator (PCNO) in simulating geothermal reservoirs. It presents novel and intriguing results for geothermal development. The authors demonstrated high computational accuracy and significant computational efficiency using the proposed PCNO method. A function to perform economic evaluations after PCNO-based emulators has also been implemented. While the research is considered to have high impact, concerns described below remain.

- It seems that this paper lacks any description of how the physical laws were learned, leaving doubts about whether predictions that satisfy the physical laws were made.
- This study described that they used a previously validated geothermal reservoir model as data, but I could not find details of this model in the cited researches. Based on the descriptions in the paper, the numerical model used is not a real-world field model but a simplified virtual model. This permeable structure does not model volcanic geothermal regions, which are typical targets in commercial operations, but rather appears to be a simplification of sedimentary layers.
- In geothermal development, production wells and reinjection wells are typically placed at different depths and designed to prevent reinjected water from overcooling the reservoir. However, in this numerical model, these wells are placed at the same depth. Given these points, it remains questionable whether the numerical model used in this study can be recognized as a realistic geothermal system.

(Remarks on code availability)

Reviewer #4

(Remarks to the Author)
This was a very thoroughly prepared manuscript. The motivation, methodology and results are very relevant and timely. However, there are a few comments raised that need to be addressed to improve the manuscript thus requiring a major revision.

Major comments:

1. Computational Speedup
• Hardware details missing: CPU/GPU specs, memory, and environment should be stated to validate the speedup ratio.
2. Data Preprocessing:
• Sampling method unspecified: How the 6,000 cases were sampled (uniform, Latin Hypercube, Monte Carlo) is not stated.
3. Model training:

• For model training hardware and runtime: Training time and resource consumption are not discussed.

4. Supplementary method 1- Numerical simulation settings
• No boundary-condition details: lateral/vertical BCs (constant T, no-flow, etc.) should be specified.
5. Model
• Validation only on synthetic CMG data, no field case or empirical benchmark - Include one field-scale validation (e.g., Dixie Valley, Soultz) or cross-compare with published field data.
6. Discussion
• The authors mention that the framework can be applied to simple EGS systems. Please provide addition context to what simple EGS systems mean, as the porosity and permeability fields of EGS differ from conventional hydrothermal geothermal systems.
• The thermal stresses associated with geothermal systems are not negligible. Kindly provide some comments on why geomechanics is not considered in the model, and what is the implication for real geothermal systems when using the PCNO framework.

Section wise minor comments:
1. Introduction:

• Line 89 – Typo – 'Neural' instead of 'Nueral'
• Line 90 – Typo – "Conservation" instead of "Conversation"

2. Results- Dataset Overview:

• Range justification: Some ranges (e.g., 1.8×10^6 J/(m·day·°C) for thermal conductivity) may not be physically consistent with typical geothermal rock values — clarification needed on units or scaling.
• Sampling strategy missing: No mention of sampling method (e.g., Latin Hypercube, uniform random, Sobol sequence).
• Training–validation split rationale: A 500-sample validation set (8%) may be small for hyperparameter optimization; mention if cross-validation was used.
• Simulator assumptions omitted: CMG-STARS setup (boundary conditions, and equation of state) should be summarized or referenced to ensure reproducibility.
• Discuss whether CMG-STARS runs include transient wellbore coupling or only reservoir-scale heat transport, if so, this avoids the need to constrain using equations 9 and 10 for wellbore modeling.

3. PCNO model architecture – None

4. Model prediction performance
• Spatial averaging: Unclear whether reported errors are grid-averaged, volume-weighted, or well-centered.
• Temporal generalization: Model performance beyond 20 years or unseen injection schedules not tested.
5. Recoverable geothermal energy potential assessments:
• Computational feasibility: Simulating 2,000 ensembles rapidly is mentioned but not quantified (runtime or computational resource).
• Unit consistency: Some plots (MWth/MWe) and energy totals (GWh) could use unified scaling for clarity
6. Economic evaluation and development plan optimization:
• Composite score definition unclear: Weighting between technical and economic terms should be detailed.
• Scaling realism: Thousands of cases evaluated, but computational time or hardware requirements are not quantified.
• Currency year: Costs lack reference year or regional adjustment (e.g., USD 2024).
• Optimization Transparency - Composite score weighting (technical vs economic) undefined. Readers cannot reproduce optimization outcomes. Present weighting formula or Pareto front of LCOE vs thermal output.
7. Computational speed-up:
• Dataset loading time: It's unclear if preprocessing and post-processing times are included in the 50 s measurement.
• No discussion of training time: Readers may question total cost (training + inference) versus repeated simulations.
• Energy efficiency: Power consumption savings (CPU-hours, CO_2 footprint) could further strengthen the argument.
8. Discussion:

• Line 386 – 'Spatial heterogeneity' instead of 'spatial heterogenous'.
• Line 435 – Typo - "FCNO" instead of "PCNO"

9. Methods: Datasets development and preprocessing
• Validation link to field data: The dataset is simulation-based only—no mention of comparison or calibration to real geothermal data.
• Temporal resolution: The frequency of recorded outputs over 20 years is not indicated (annual, quarterly?).
• Fixed pressure gradient assumption: Simplifies depth-pressure relation—should mention if sensitivity analysis confirms minimal bias.

10. Evaluation metrics for model training:

• Weight selection unexplained: The rationale or tuning process for w_1–w_{11} is not discussed.

11. PCNO model training procedure:

• Optimization details are missing: No mention of optimizer type (Adam, RMSprop), learning rate, or batch size.

12. Embedded power estimation module for geothermal applications:
• Empirical origin unclear: The source of Eq. (13) (reference 36) should be described—whether derived from regression on global plant data or literature fits.
• Assumed constants: Capacity factor (0.85) and zero efficiency below 75 °C could vary regionally or by plant type; sensitivity analysis is missing.
• Equation Validity - $\eta = 0.1268 \ln(T\_pro) - 0.4915$ can yield $\eta > 1$ for high T. Cap $\eta \leq 0.25 - 0.30$ and cite the empirical source range.
13. Integrated economic module for financial assessment:

• Currency and base year unspecified: All cost functions need normalization (e.g., USD 2024).
• O&M labor split: "75 % labor" could be clarified—does this apply annually or as average over lifetime?
• Line 614 - Typographical issues: "include expenditures" → "includes expenditures".

14. Supplementary Methods - Supplementary Method 1: Numerical modeling settings:
• Grid simplification: Single-layer (1 z-cell) may under-represent vertical gradients or convection effects.
• Thermal equilibrium assumptions: initial conditions (T–P distribution) not explicitly stated—hydrostatic? steady-state?
• Injection rate units: "kg/m^3" likely meant "kg/s" or "kg/s per well"; should be checked.
• No timestep description: temporal resolution and solver settings (implicit/explicit) omitted.
• Line 80 – Reference missing for injection rate.
15. Supplementary Method 2: Governing Equations Integrated into the PDCO Framework:

• Line 97 and 98 - Typographical inconsistency: Framework name alternates between PDCO and PCNO should be consistent.
• Radial inflow model (Eq. 6): The λ term is ambiguous used for relative mobility, but notation overlaps with thermal conductivity.
• Equation dimensionality: Eq. (6) uses "q (kg/m^3)"—should likely be mass flow rate (kg/s) or volumetric flow rate (m^3/s).
• Fluid property models: Piecewise viscosity functon(Eq. 12) may cause discontinuities near thresholds (40 °C, 100 °C); smoothing suggested.
• Line 172 - Typo – Supplementary table 1 should be stated instead of 2.

16. Supplementary Method 3: Physics-Constrained Neural Operator Structure.

• Table 4 comparison: ANN-FNO faster than FNO + GPF but less accurate—brief rationale would help (overhead vs complexity).

17. Supplementary tables

• Typo – At supplementary table 1 change the reference equation 11 to equation 13 for the co-efficients A to H.

(Remarks on code availability)
The code ran successfully with just a minor warning "+ python -u PCNO_Ele_Eco_Module.py | /code/PCNO_Ele_Eco_Module.py:212: DeprecationWarning: Conversion of an array with ndim > 0 to a scalar is deprecated, and will error in future. Ensure you extract a single element from your array before performing this operation. (Deprecated NumPy 1.25.)". Guidance on how to extract the single array.

Version 1:

Reviewer comments:

Reviewer #1

(Remarks to the Author)
The authors are to be congratulated for their thorough and considered responses to the reviews. The revised article is considerably improved for their diligent hard work. Well done!

I have one comment on their responses and one small correct listed below.

Response number (1)

The authors still do not have this quite right. It is true that "the standard workflow in many geothermal reservoir studies, where a separate natural-state model is used to establish stable pressure and temperature fields, which are then taken as

initial conditions for dynamic production simulations." But in large-scale, hot, convecting geothermal systems it is not possible to "apply initial temperature and pressure distributions that represent the natural state" and you must "re-simulate long-term natural convection and background flow" to ensure it is consistent with each sample model's permeability distribution and boundary conditions.

It is also true that often (but not always) "During active production, the pressure drawdown and overpressure around wells generate pressure-driven flow that is much stronger than the slow background flow caused by natural convection." However, production temperature decline can be small and the effect an assumed initial temperature distribution being inconsistent with the model's permeability distribution and boundary conditions may be non-negligible in comparison.

However, given the content of the article, the careful wording the authors have used and the example models presented, I am happy enough with the authors' explanation.

For future reference though, in cases where the initial temperature distribution is complex with strong convection and vertical flows, you must always rerun natural state simulations to determine the correct initial conditions.

I spotted one small error also:
line 226: "simplify" not "simplified"

(Remarks on code availability)

Reviewer #2

(Remarks to the Author)
I co-reviewed this manuscript with one of the reviewers who provided the listed reports. This is part of the Nature Communications initiative to facilitate training in peer review and to provide appropriate recognition for Early Career Researchers who co-review manuscripts.

(Remarks on code availability)
yes the code is running correctly

Reviewer #4

(Remarks to the Author)
The authors have thoroughly addressed the comments previously raised. I recommend the manuscript for publication.

(Remarks on code availability)

Open Access This Peer Review File is licensed under a Creative Commons Attribution 4.0 International License, which permits use, sharing, adaptation, distribution and reproduction in any medium or format, as long as you give appropriate

credit to the original author(s) and the source, provide a link to the Creative Commons license, and indicate if changes were made.
In cases where reviewers are anonymous, credit should be given to 'Anonymous Referee' and the source.
The images or other third party material in this Peer Review File are included in the article’s Creative Commons license, unless indicated otherwise in a credit line to the material. If material is not included in the article’s Creative Commons license and your intended use is not permitted by statutory regulation or exceeds the permitted use, you will need to obtain permission directly from the copyright holder.
To view a copy of this license, visit https://creativecommons.org/licenses/by/4.0/

Responses to Editor and Reviewers

Dear Editor and Reviewers:

We sincerely appreciate your comments and suggestions on our manuscript. They are constructive, comprehensive, and highly valuable for improving our manuscript. We have carefully revised the manuscript to address each of them and hope that the revised version would be acceptable for publication in ***Nature Communications***.

Sincerely yours,

Zhenqian Xue, Jianfei Bi, Haoming Ma, Zhe Sun, Zhangxin Chen

Response #0 - Overall improvements in the revised version:

Based on the suggestions from all the reviewers, we have updated the PCNO framework by rebuilding the dataset and retraining the model. The key improvements are given as follows:

(1) The model is extended from 3D (2D space + time) to 4D (3D space + time), enabling it to capture coupled fluid flow and heat transport in both the horizontal and vertical directions.
(2) The pressure gradient is added as a new input feature so that the model can account for different initial reservoir pressure conditions.
(3) The permeability range is expanded from 50-300 mD to 0.1-300 mD, and the number of realizations is increased from 1,000 to 5,000 to represent a broader diversity of permeability fields. The porosity realizations are correspondingly expanded.
(4) The depths of injectors and producers are added as new input features so that the PCNO can capture cases where the injectors and producers are perforated at different depths.
(5) The time-varying injection-rate schedule is incorporated into the input variable "injection rate," allowing both constant and variable injection schemes to be considered.
(6) The wellbore dynamics module is updated to a two-phase flow wellbore model that can evaluate phase changes, pressure losses, and temperature evolution.

Based on these updates, we have also revised the corresponding sections in the manuscript and the Supplementary Information.

Note: Table 1-2 and Figures 1-6 are provided in the manuscript. Methods S1-6, Figures S1-10, and Tables S1-4 are provided in the Supplementary Information.

Reviewer #1 (Remarks to the Author):

Thank you for your valuable suggestions, which have significantly enhanced the quality of our manuscript. We have provided point-by-point responses below, detailing how each comment has been addressed.

General comments

1. The authors should be congratulated for an interesting, well-written paper.

   However, in my opinion they overstate the paper's impact and their work does not demonstrate the broad generalizability they claim. Their work focuses on a geothermal system that has the following characteristics:

   - No natural convection or fluid movement. Allowing assumed initial temperature and pressure distributions.

   - No phases changes (no boiling). Allowing single phase treatment of the fluid.

   - Horizontal flow only. Therefore, no buoyancy effects or vertical flows.

   - Limited types of geological heterogeneity with no faults and generally medium to high permeability (50 – 300 mD).

   - Simple wellbore dynamics without multiple feedzones, or phase changes.

   - Simple production and reinjection strategies.

   These assumptions may be valid for low-temperature geothermal systems in certain cases, but they significantly restrict the applicability of the approach to a broad range of geothermal systems. Low-temperature, single-phase geothermal systems that are dominated by horizontal flows are important to consider in geothermal energy, but they are not representative of the broad range of systems.

   It is possible that the author's new framework could be applied more broadly but they have not demonstrated that in this work.

   I recommend that the authors carefully rewrite the article to accurately reflect the limited types of geothermal systems that they are studying and discuss the impact of their work considering the contribution of those types of systems to the broader field of geothermal energy.

   Response: Thank you for these comments.

   We agree that the original submission did not describe clearly enough the scope and limitations of the PCNO, which may have led to an overstatement of generalizability. Following your suggestions, we rebuilt the dataset, retrained the model and updated the framework, as summarized in our response to Comment 0. In the revised manuscript, we also provide a clearer statement of both the strengths and the limitations of the work in the Discussion section.

   The followings are the responses to your General Comments.

(1) **Regarding natural convection or fluid movement:** We agree that our simulations do not resolve natural convection or background fluid flow and instead use assumed initial temperature and pressure distributions.

This follows the standard workflow in many geothermal reservoir studies, where a separate natural-state model is used to establish stable pressure and temperature fields, which are then taken as initial conditions for dynamic production simulations. Consistent with this, we apply initial temperature and pressure distributions that represent the natural state and do not re-simulate long-term natural convection and background flow. This initial state can be viewed as the result of geological time-scale heat and mass transport that has produced a stable geothermal gradient and a near hydrostatic pressure profile.

During active production, the pressure drawdown and overpressure around wells generate pressure-driven flow that is much stronger than the slow background flow caused by natural convection. On time scales of years to a few decades, these well-induced pressure gradients are typically much larger than those associated with slow natural convection, so production-driven flow paths control the main patterns of mass and heat extraction. Under these conditions, treating natural convection as a process that establishes the initial state and neglecting explicit natural convection during the production phase is a commonly adopted assumption in dynamic geothermal models.

For these reasons, our reservoir simulations and the surrogate models trained on them do not include natural convection or background flow during production. These processes are represented through the assumed initial pressure and temperature fields, and the dynamic model focuses on production-induced changes that dominate system behaviour over project-relevant time scales.

(2) **Regarding phase change:** We agree that our model treats the working fluid as single-phase water. This assumption is consistent with the pressure and temperature conditions considered in this work. In the reservoir, the initial temperature and pressure ranges are 90-250 °C and 12-48 MPa. During operation, after applying injection and production constraints, these conditions remain within 20-250 °C and 5-47 MPa. According to the standard water phase diagram, these conditions stay in the liquid region, so a single-phase description in the reservoir is appropriate throughout our simulations.

We acknowledge that the present work does not cover two-phase geothermal reservoirs. Our investigation suggests that a unified surrogate that simultaneously covers both single- and two-phase geothermal systems, while allowing for high-dimensional inputs and outputs, and full 4D state prediction, would require much larger training ensembles, multi-resolution grids, and more tightly coupled physics. This would substantially increase the computational cost of data generation and training and could make optimization less tractable. Therefore, we suggest that a more practical path is to develop a dedicated

surrogate for two-phase geothermal systems, with parameter ranges tailored to such systems, and then use these together with the present PCNO to cover a wider range of geothermal resource types. We have clarified this limitation, its rationale, and potential directions for extension in the revised Discussion section.

At the same time, we also recognize that even in single-phase geothermal systems, phase change can occur in the wellbore during upward flow. To account for this, in the revised version, we have updated the wellbore dynamics to a two-phase flow model that captures possible flashing, pressure drop and heat loss along the wellbore, supporting more accurate prediction of production temperature and enthalpy at the surface. (Method S2)

(3) **Regarding horizontal flow only:** In the revised version, the computational domain of the PCNO model has been extended to a full 4D domain (3D space + time). With this update, the model now predicts pressure and temperature on a three-dimensional grid and therefore captures fluid flow and heat transport in both the horizontal and vertical directions. Its prediction performance in different spatial directions is shown in Figures 2, S4 and S5.

(4) **Regarding limited types of geological heterogeneity and permeability range:** we agree that the current PCNO model does not explicitly represent complex geological features such as faults, and this is a limitation of the present study. Incorporating fault networks into AI-based subsurface flow models remains challenging because available field data on faults are limited, which is hard to support a systematic and general parameterization of fault systems for large training ensembles. We also recognize that faults can be important for the performance of geothermal systems, so this will be a key direction for our future work. We have clarified this limitation in the revised manuscript in the ‘Limitations and opportunities’ part of the Discussion section.

For permeability, we have extended the range used to generate the datasets from 50-300 mD to 0.1-300 mD and increased the number of realizations, so that the model is trained over a broader set of reservoir conditions. This wider range allows the model to be more applicable to reservoirs with lower permeability. The surrogate is not limited to individual realizations in the training set but learns to interpolate across heterogeneous permeability fields whose values fall within this range.

(5) **Regarding simple wellbore dynamics:** In the revised version, the wellbore dynamics model has been updated to a two-phase flow formulation. This model captures possible phase change, pressure drop, and heat loss along the wellbore and supports more accurate prediction of production temperature and enthalpy at the surface. (Method S2)

(6) **Regarding simple production and reinjection strategies:** We note that realistic well controls in geothermal fields are influenced by many factors and are often strongly site specific. However, detailed field data on such controls are limited, which makes it difficult

to construct large training ensembles and to build AI-based subsurface models that systematically represent a wide range of realistic well-control schemes in a consistent and general way. As a result, most existing AI-based subsurface flow models (e.g., geothermal, oil and gas) assume fixed or simplified well controls, and even many numerical simulation studies adopt fixed injection and production settings for system performance evaluation.

In line with this practice, the original version of our model used fixed injection rates and fixed production pressures. To improve flexibility, the revised version now allows time-varying injection rates, so that different injection profiles over the project lifetime can be represented within the same framework. The detailed description and model prediction results on time-varying injection schedule can be found in Method S1 and Figure S6.

We recognize that the present setup still does not cover very complex or site-specific control schemes. However, the improved treatment of well controls already supports systematic evaluation of long-term production performance and provides guidance for development planning. We agree that more complex well control strategies are an important direction for future work and we have stated this in the Discussion section as one of the main limitations and planned extensions.

Specific comments

2. Line 31 & 37 – I could not find the information:

“Global geothermal 30 resources are estimated to be 100 to 1,000 times greater than those of fossil fuels.” in the Barbier reference given. Regardless these types of estimates usually include the energy potential of EGS and AGS. The comment about barriers on line 36 is really referring to conventional geothermal. The barriers for EGS and AGS are associated with drilling and fracturing technology rather than resource uncertainty. It’s misleading to indicate that by providing better tools for assessing reservoir potential and performance the potential from EGS and AGS would be unlocked. I agree that better tools for assessing reservoir potential and performance are very important. But the authors should be more careful about linking the potential.

Response: Thank you for this comment.

We agree that the global resource estimates already include the potential of both conventional geothermal systems and engineered concepts such as EGS and AGS and that the main barriers are different between these system types. To avoid overstating the role of reservoir assessment tools in “unlocking” the full potential of EGS and AGS, we have revised the Introduction to clearly separate these aspects and to emphasize that improved tools mainly help reduce project risk and support better design and decision-making, rather than directly unlocking the full potential of EGS and AGS. The revised text is provided in the Introduction section.

3. Line 117 - "common simplification in geothermal reservoir modeling" – These types of simplifications are only usually made during initial stored heat calculations. Generally, it is the non-uniformity, heterogeneity, faulting, geological offsets etc. which necessitate numerical modelling. By simplifying these away the need for numerical modelling in the first place is reduced.

   Response: Thank you for this comment.

   We agree and have reduced the level of simplification in the revised PCNO model. In the revised version, only four parameters are treated as scalars, including reservoir depth, target reservoir thickness, rock thermal conductivity, and rock heat capacity. Relative to the previous version, initial reservoir pressure and temperature have been removed from the scalar list and are now represented as 3D fields on the grids. These fields are computed from the reservoir depth together with pressure and temperature gradients, so spatially varying initial conditions can be captured. The target reservoir thickness remains a scalar because it is defined by the difference between the upper and lower boundaries of a target zone and typically has a single value for each case. The rock thermal conductivity and heat capacity are also kept as scalars, since they are usually close to uniform within the target zone of a reservoir and previous studies have shown that they have a relatively small impact on the uncertainty of geothermal production[1], which is also often adopted in many numerical reservoir modeling for geothermal systems. We have updated the corresponding text in the manuscript.

   With respect to non-uniformity and structural offsets, the revised PCNO can represent irregular reservoir geometries by assigning zero values to grid cells outside the reservoir for all field variables. This masking allows variations in reservoir extent to be included directly in the input fields. A detailed description and examples of model performance for irregular geometries are provided in Figure S7.

   Regarding faults, we agree that the current model does not explicitly represent fault networks and that this is a limitation of the present work. This point is discussed in Response (4) to the General Comments and is also highlighted in the Discussion, where it is identified as an important direction for future work.

4. Line 211 - "In summary, the PCNO model exhibits strong predictive accuracy and generalization capability. Its ability to deliver real-time forecasts for both subsurface reservoir and well behavior and surface-level production metrics enables a comprehensive understanding of geothermal system dynamics." This is a misleading statement. The authors have tested a very limited range of geothermal conditions and therefore the method has only been shown to enable a comprehensive understanding of that very limited range. Also, the range of conditions considered are amongst the simplest geothermal conditions.

   Response: Thank you for this comment.

We agree that the original wording was too strong and could be interpreted as claiming validity beyond the limited set of geothermal conditions considered. In response, we have both updated the PCNO framework and revised the corresponding statements to more accurately reflect its capabilities and scope.

In the revised manuscript, we now describe the PCNO as providing accurate, time-resolved predictions of subsurface states and surface production metrics within the range of reservoir and operating conditions considered, and as supporting screening-level assessment for considered types of geothermal systems, rather than all geothermal systems.

We have also expanded the PCNO framework relative to the original version. The updated model (1) resolves the full 4D domain and considers fluid and heat flow in both the horizontal and vertical directions; (2) captures phase, pressure, and thermal changes in the wellbore; (3) is trained on a broader parameter space that includes lower permeability and spatially varying initial pressure and temperature; (4) can represent non-uniform reservoir geometries, variable well depths, and both constant and time-varying injection schedules. Within these ranges, the PCNO reproduces reservoir fields and task-specific outputs with low errors across different reservoir conditions and operating strategies.

At the same time, we fully acknowledge that the present work still focuses on a restricted class of systems. The current PCNO is not intended to provide accurate predictions for strongly two-phase reservoirs, explicit fault networks, fine scale engineered fractures, or highly site-specific well control strategies. These limitations and the reasons for them are now stated in the revised Discussion section.

To place our contribution in context, we also compare the revised PCNO with existing AI-based geothermal surrogate models. To the best of our knowledge, earlier studies typically: (1) focused on more simplified reservoir conditions and often in 2D domains, without explicitly accounting for reservoir depths, rock properties, target reservoir thickness, temperature and pressure gradient; (2) used restricted operating settings, typically with fixed well numbers, locations, depths, and well controls; (3) concentrated on reservoir temperature and/or pressure prediction without coupling to wellbore dynamics, and thus could not predict surface-level heat or power; (4) addressed only technical performance without integrated economic assessment.

Compared with these existing surrogates and tools, the revised PCNO offers improved generalization and broader functionality. Therefore, despite the current limitations, the PCNO framework provides a useful basis for extending surrogate methods to more complex and realistic settings and offers a transferable template for surrogate modelling and surrogate-based evaluation of geothermal and other subsurface energy systems. The revised Discussion section summarises these advances, clarifies the remaining limitations, and outlines directions for future development.

5. Line 236 - "enable large-scale ensemble modeling and probabilistic assessments that were previously impractical". This is not accurate any longer. Large-scale ensemble modeling and probabilistic assessments are being carried out in geothermal. eg:

   Wang, Y., Voskov, D., Daniilidis, A., Khait, M., Saeid, S., & Bruhn, D. (2023). Uncertainty quantification in a heterogeneous fluvial sandstone reservoir using GPU-based Monte Carlo simulation. Geothermics, 114, 102773.

   Line 303 - "However, existing workflows typically rely on separate tools for economic evaluation and field optimization, resulting in significant time demands and manual effort." This is broadly true however some authors have already made progress on coupling the physical simulations with techno-economic assessments. Again see:

   Wang, Y., Voskov, D., Daniilidis, A., Khait, M., Saeid, S., & Bruhn, D. (2023). Uncertainty quantification in a heterogeneous fluvial sandstone reservoir using GPU-based Monte Carlo simulation. Geothermics, 114, 102773.

   Response: Thank you for these two comments.

   We have revised these sentences to more accurately reflect the current status of ensemble modeling and probabilistic assessments. We have also discussed this point in the Introduction.

6. Line 324 - "By jointly considering technical performance, economic cost, and operational constraints, it strengthens decision-making and accelerates the planning and deployment of geothermal energy projects." This is true but the details of the techno-economic modelling are important. The description in the Integrated economic evaluation module for financial assessment section is fairly brief. Are drilling depths always constant? What wellbore sizes are considered? How are rig mobilisation costs calculated? Are all of these costs considered to be constant over time? Therefore, assuming all the wells are drilled at the start of the project. What about the off-taker agreements e.g. Power purchase agreement?

   Response: Thank you for this comment.

   In the revised version, we have provided a more detailed description of the economic module in Method S6. Here we briefly summarize the main modeling choices related to your questions.

   The drilling depth is not assumed to be constant. In each case, the well depth used in the economic model is set equal to the corresponding injector or producer depth used in the cases feed PCNO, so well costs are evaluated as a function of the well depth. As described in Method S6, well costs are calculated using a published depth-cost correlation that was fitted to a compiled database of hydrothermal and EGS wells. This relationship was calibrated against reported total well costs, which include the major cost components typically recorded in project budgets such as drilling services, casing, cementing and rig mobilisation and demobilisation. Because the underlying database contains wells with different diameter designs, the average effect of wellbore size is implicitly embedded in the correlation. For this reason, in the

economic assessment, we use the cost-depth correlation directly for the screening-level assessment and do not add a separate dependence on the wellbore size. In the two-phase wellbore model, we assume a constant wellbore diameter of 0.2 m across all the scenarios.

With respect to cost timing, capital expenditures are assumed to occur at the start of a project, and drilling costs are, therefore, treated as fixed in time once incurred. O&M costs are modeled as recurring annual costs over the project lifetime and are discounted using a discount rate. We recognize that, in some real projects, wells may be drilled in stages rather than all at the beginning of operation. However, this timing is highly project-specific, and the economic module in this work is designed as a general screening-level techno-economic tool for comparative evaluation across a wide range of geothermal scenarios. Therefore, we assume that all wells are drilled at the start of a project for this screening focus.

For off-taker agreements, the present framework focuses on the levelized cost of heat (LCOH) and the levelized cost of electricity (LCOE) as the main economic indicators. These metrics emphasize the cost of produced energy and do not explicitly represent revenue streams or contract structures. Therefore, power purchase agreements and other off-take arrangements are therefore not modelled within the current economic module.

7. Line 351- "Once trained and validated, the model can handle a wide range of input conditions that are critical for geothermal systems and eliminates the need for labor-intensive model construction typically required in traditional workflows." In this paper the authors have trained the model using a very simple type of geothermal system with very simple strategies for utilisation. How do the authors propose to achieve this for realistic complex systems? Running thousands of realistically complex reservoir models to train the model prior to use will be extremely computationally expensive.

   Response: Thank you for this comment.

   To more precisely describe this statement, we have revised this sentence to ‘Once trained and validated, it can handle diverse input conditions for geothermal evaluation within the considered ranges and eliminates the need for labor-intensive model setup required in conventional workflows.’

   At the same time, the capability of PCNO has been improved relative to the original version, as detailed in our Response to Comment 4. Specifically, the revised PCNO now uses a full 4D formulation, is coupled to a two-phase wellbore model, includes a broader set of inputs (e.g., an extended permeability range, spatially varying initial pressure and temperature, different well depths, and time-varying injection rates), and can be used for non-uniform reservoir geometries. These updates allow a more precise description of the tested geothermal systems and cover a wider class of reservoir conditions and operating schemes than in the original version. We also acknowledge that the updated PCNO remains limited to certain types of geothermal systems, such as strongly two-phase systems with extensive fault networks, fine-

scale fracture systems, or project-specific well control strategies. These limitations and their rationale are discussed in Response to Comment 4 and in the "Limitations and opportunities" section of the revised Discussion section.

Within the current setup, the computationally efficient PCNO can be used to evaluate different geothermal systems whose reservoir conditions fall within the ranges of the training data. It is designed to assist operators and planners by enabling fast ensemble modeling that improves the understanding of a target reservoir. In specific, it can assess recoverable geothermal energy potential, evaluate combined technical and economic assessments, screen different utilization options, and identify promising ranges of operating conditions (e.g., an injection rate, production pressure, well counts, well spacing and well numbers). Although the resulting ranges of operating conditions may not be directly used as final design values for field operation, they can narrow the search space and provide useful guidance on potentially optimal operational schemes. Based on this, the PCNO can save much time during the evaluation period by reducing the need for repeated, labor-intensive model construction. In addition, modules such as power estimation and economic modules are implemented as configurable external components and can be adjusted to match project-specific assumptions without retraining the surrogate.

For more realistic and highly complex systems, our goal is not to claim a single surrogate that covers all possible configurations. As discussed in the Response to Comment 4 and in the revised Discussion, the improved generalizability and broader applicability of the PCNO, relative to existing geothermal surrogate models and evaluation tools, make it a transferable template that can be adapted or retrained for specific system classes. For example, a dedicated two-phase geothermal surrogate can be created, with parameter ranges tailored to such systems, and then use this together with the present PCNO to cover a wider range of geothermal resource types. Similarly, once sufficient high-fidelity data are available in a form that supports systematic parameterisation of faults or more complex well controls, these features can be incorporated into extended surrogate models. Generating surrogates for such complex systems is indeed computationally demanding, but this cost is incurred once and can then be spread over many subsequent evaluations. In contrast, performing large ensemble-based assessments directly with full-physics models would require repeating the full simulation cost for every scenario and project.

8. Line 358 - "In contrast, traditional simulation workflows often require dozens to hundreds of hours due to complex model configurations and iterative solution steps." This is true in general but for the simplified reservoir and production strategies investigated the computation and setup costs could be much lower.

Response: Thank you for this comment.

In the revised version, we have rewritten the entire 'Computational speed-up' section. Based on our updated tests, because of the coupling with a two-phase wellbore model and the inclusion of more complex reservoir and operational conditions, CMG-STARS requires about 7,380 minutes to simulate 1,050 cases. In particular, numerical simulators typically need from tens of seconds to several minutes for initialization between runs. This overhead is minor for a single case or a small number of runs, but for large-scale ensemble modeling it accumulates to several hours and cannot be neglected.

In contrast, the PCNO can evaluate the same 1,050 cases in approximately 310 s, which corresponds to a speedup of more than 1,400-fold. In the revised text, we show the comparison in terms of these timings for the 1,050-case benchmark, rather than making a general statement about all traditional workflows. We have also provided a more detailed description of computational cost for different models, and comparisons across model complexity and ensemble size, is provided in Table S3 and Figure S9.

9. Line 366 - "It is also important to note that the computational cost of conventional simulations increases with model complexity and solver settings, whereas the prediction time of the PCNO remains effectively independent of case complexity." How has this been demonstrated? This is a fundamental issue with the work.

   Response: Thank you for this comment.

   The prediction time of the PCNO is mainly controlled by the hardware and is largely insensitive to the physical complexity of an individual case, whereas the runtime of conventional simulations increases with model complexity and solver effort.

   To demonstrate this point, we compare the prediction times of the PCNO and CMG STARS for cases with different levels of model complexity. In this test, we construct a series of five models with progressively increasing case complexity by increasing the reservoir area (active grids), increasing the heterogeneity of permeability and porosity, increasing the number of wells, and including a coupled wellbore model. For CMG-STARS, each of these changes leads to a clear increase in simulation time. In contrast, for the PCNO, the prediction time remains essentially constant across these cases, because the forward evaluation of the trained network is not sensitive to the internal physical complexity encoded in the input fields. This behaviour reflects the fact that, once the network is trained, the cost of a forward pass does not depend on the internal physical complexity encoded in the input fields. In other words, for a fixed model configuration, changing the degree of heterogeneity, adding wellbore physics or modifying the number and type of wells does not change the number of floating-point operations in the PCNO evaluation, whereas it does affect the number of time steps and nonlinear iterations required by CMG-STARS.

We also compare the time costs of PCNO and CMG-STARS for different numbers of test cases. The results show that the computational speedup achieved by the PCNO is more obvious as the number of the evaluated cases grows.

The detailed description of these results is provided in Figure S9.

10. Line 381 - "A key highlight of this framework is its broad generalizability." How has this been demonstrated? Having random permeability and porosity distributions within a two-dimensional domain does not demonstrate broad generalizability regardless of how "realistic" the Spectral Random Field generation method is.

Response: Thank you for this comment.

We agree that the original phrase “broad generalizability” could be misleading. Our intention was not to claim applicability to all geothermal settings, but to indicate that, within the considered ranges, the proposed framework generalizes well over the set of inputs and outputs used in this study and shows improved generalization compared with previously published AI-based geothermal surrogate models (as illustrated in our Response to Comment 4).

As illustrated in previous responses, in the revised version, we have also updated the PCNO and improved its generalizability compared with our previous version. We have also removed wording such as “broad generalizability” and replaced it with a more careful description. We have also revised the Discussion section to provide a clearer summary of the improvements achieved by the current framework and the main limitations that remain.

11. Line 390 - "Our model addresses this limitation by using spatial and temporal encodings that define well settings in a generalizable format." This is also overstated. While the authors may have advanced this approach from simple well configurations, the configurations considered cannot be called complex.

Response: Thank you for this comment.

We agree that the original statement was overstated. Our intention was not to suggest that the framework can represent all complex well configurations, but rather to indicate that it moves beyond the very simple, fixed well settings that are commonly used in previous AI-based geothermal surrogate models.

In the revised version, the PCNO can handle more flexible well configurations than our earlier version, including different well counts, placements, depths, and both time-varying and constant injection rate schedules. Compared with existing studies that typically assume unchanged well number, location, depth and controls, this represents a clear step forward in how well configurations are handled in a surrogate framework. With this setting, the PCNO can be used at a screening level to evaluate geothermal systems under multiple alternative well layouts and operating strategies, which provides more informative guidance than surrogate models that rely on fixed and highly simplified well settings.

We also fully acknowledge that the current PCNO does not yet capture very complex and highly site-specific well architectures. This limitation is discussed in earlier responses and in the revised Discussion section. However, the introduced treatment of well configurations can provide a useful basis for future work on representing variable well patterns within a tractable surrogate model and can be further developed as more detailed field data on realistic well patterns and control schemes become available.

In line with this, we have revised the corresponding statements in the manuscript to remove the overstated claim and to present more clearly both the advantages and the remaining limitations of the present framework, and we highlight further extension to more complex well configurations as an important direction for future work.

12. Line 585 - "Power plants such as dry steam, flash steam, and binary cycle systems exhibit different requirements for the temperature of the produced fluid in order to generate electricity effectively." This comment seems to indicate the authors considered various types of geothermal systems. However, all the work presented only describes single-phase, non-convecting geothermal systems.

Response: Thank you for this comment.

In this work, the reservoir fluid is restricted to the single-phase liquid region within the tested pressure and temperature ranges, and two-phase reservoir behavior is not included in the current PCNO. We have emphasized this limitation and its reasons in the revised Discussion and identified an extension to two-phase reservoir conditions as an important direction for future work.

At the same time, phase change can still occur in the wellbore even when the reservoir remains single phase. For this reason, in the revised version we have updated the wellbore model to a two-phase flow formulation. This allows the PCNO to account for possible phase change along the wellbore and to predict the fluid temperature and enthalpy at the surface based on its phase state when it reaches the surface (details are shown in Method S1). These surface conditions are then used as inputs to the power-estimation module.

The sentence referring to dry steam, flash steam and binary-cycle plants was not intended to suggest that we explicitly simulate these different geothermal system types in the reservoir model. Rather, it was meant to explain why we adopt an enthalpy-dependent empirical efficiency correlation derived from a compilation of worldwide geothermal power plant data, instead of using a single fixed efficiency or simplified correlations. We have revised this sentence accordingly to avoid misunderstanding.

Finally, although the initial state of the reservoir is assumed to be in equilibrium with no flow and no temporal changes, both conductive and convective heat transport are included during the production phase, as shown in Eq. 2 in Method S1.

Supplementary Information

13. Line 23 - "Given that geothermal energy extraction predominantly involves horizontal fluid flow and heat transfer, the model applies 80 grids in each horizontal direction and a single grid in the vertical direction, resulting in a total of 80 × 80 × 1 cells" - This is not true in general. Vertical fluid flow is very important in convecting geothermal system (which account for the majority of systems used for electricity generation).

    Response: Thank you for this comment.

    We agree that vertical fluid flow can be very important. Therefore, in the revised version, we have regenerated the training dataset and rebuilt the PCNO using a fully 3D reservoir grid. The CMG-based numerical model now uses 80 cells in each horizontal direction and 5 cells in the vertical direction, resulting in 80 × 80 × 5 cells instead of 80 × 80 × 1. The revised PCNO is trained on this 3D configuration and therefore captures fluid and heat transport, as well as associated pressure and temperature variations, in both the horizontal and vertical directions.

    We have corrected the corresponding description in the manuscript to reflect this change, and the prediction performance in different spatial directions is presented in Figures 2, S4 and S5.

14. Line 39 - "which means abnormal geological settings such as faults, overpressured zones, or vapor-dominated reservoirs are not considered." Faults and overpressured zone are not abnormal geological settings but are in fact encountered in many if not most convecting geothermal systems. Vapor-dominated systems are rare, but I would not consider them abnormal. Complex 2-phase flow found in vapor-dominated systems is also encountered in liquid-dominated systems during production.

    Response: Thank you for this comment.

    In the revised version, we no longer refer to faults, overpressured zones, or vapor dominated reservoirs as “abnormal”. To allow for different pressure regimes, we now treat the reservoir pressure gradient as an input feature and sample it within a range of 7-15 MPa/km, so that elevated pressure gradients can be represented.

    As discussed in the above responses, faults and fully two-phase flow systems are still not explicitly represented in the current implementation. We have identified these points as limitations, explained the reasons in the Discussion, and indicated them as important directions for future work. The corresponding sentence in the Supplementary Methods has also been revised to remove the misleading wording and to more accurately describe the modeled system.

15. Line 52 - "permeability ranges from 50 to 300 mD" - This would be considered medium to very high permeability for convecting geothermal systems.

    Response: Thank you for this comment.

In the revised version, we have regenerated the training dataset and rebuilt the PCNO so that reservoir permeability now ranges from 0.1 to 300 mD, and we have also increased the number of realizations. This extended range and larger ensemble allow the dataset to include lower-permeability conditions and provide a more representative sampling of geothermal reservoir settings, improving the ability of the PCNO to generalize across different permeability conditions within this value range. We have updated the relevant description in the manuscript, as shown in Method S1.

16. Line 69 - "As most geothermal reservoirs remain in the early stages of development, initial operation typically involves a limited number of wells. Accordingly, the number of injection and production wells is assumed to range from 1 to 4 each". It is true that geothermal developments start with a limited number of wells. However, production scale systems often have tens or hundreds of wells. Carrying out a resource assessment assuming only a few wells will not accurately stress systems.

Response: Thank you for this comment.

We agree that mature geothermal developments can involve many tens or even hundreds of wells. In the present work, however, our focus is on screening-level project evaluation, rather than on fully built-out field developments. The choice to consider 1-4 injectors and 1-4 producers in each scenario reflects this focus and also the practical need to keep the control space manageable for training and uncertainty analysis.

In practice, many geothermal projects begin with a limited number of wells that are used for pilot production, stepwise expansion and concept testing. Even in large developments, field layouts may often be organized as similar well patterns or modules. In this context, a surrogate that can capture the response of a reservoir to a small set of injectors and producers can still provide information for screening operating strategies, understanding key sensitivities and deriving pattern-scale performance metrics that can inform the design of larger developments.

From a modeling perspective, increasing the number of wells directly enlarges the control space (well counts, placements and control schedules) and introduces higher-order well-well interactions. Capturing the associated behavior in a surrogate model would require a much larger ensemble of high-fidelity simulations. Under current data and computational constraints, this would limit our ability to explore other important sources of uncertainty, such as geology and operating conditions, within a single surrogate framework. We, therefore, restrict the number of injectors and producers to 1-4 in each case in order to maintain reasonable coverage of the parameter space while keeping the training problem manageable.

Even with this limitation, the PCNO already shows clear improvements over existing geothermal surrogates, which typically assume very few wells and fixed, simplified well configurations. Its ability to handle variable well numbers, locations, depths and injection schedules within the 1-4 well regime represents a step forward for screening-level assessment.

In the revised version, we have clarified this scope in Method S1 and have noted that extending the PCNO framework to larger well counts, more representative of full production scale fields, is an important direction for future work.

17. Line 90 - "The production pressure is primarily determined by the initial reservoir pressure and is typically maintained at a lower value to promote fluid flow toward the production wells." Again, this is only true in the case of low temperature geothermal projects. Production pressure in higher temperature projects is determined by many factors including separation pressure, temperature for scale formation, and electricity generation technology.

    Response: Thank you for this comment.

    We agree that, in higher temperature geothermal projects, the production pressure is influenced by many factors, rather than only by the initial reservoir pressure.

    In our framework, production pressure is treated as an operational control variable within a reasonable range rather than being derived from a detailed model of surface facilities and scaling constraints. In practice, bottom-hole pressures would be selected after integrated design of wells and surface facilities, which is highly site- and project-specific. To provide a general and transferable representation of production control at the reservoir scale, we therefore impose a pressure drawdown by setting production pressure below the initial reservoir pressure, which is a common assumption in reservoir simulation to maintain flow toward production wells.

    In the revised manuscript, we have removed the statement that production pressure is “primarily determined by the initial reservoir pressure” and replaced it with a more careful description to avoid confusion for readers, as illustrated in Method S1.

18. Line 97 - The governing equations that have been used are for single phase fluid (or an equivalent mixture version) that cannot capture the correct two-phase fluid dynamics. Similarly, the wellbore dynamics equation is a very simplified representation of the complex governing equations for wellbore flow.

    Response: Thank you for this comment.

    As discussed above, the current framework is developed for single-phase, liquid-dominated geothermal conditions and, therefore, does not model two-phase flow within the reservoir. The training and test cases were screened to remain in the liquid region based on water phase behavior, and its thermophysical properties (e.g., density, viscosity, and enthalpy) are updated as functions of pressure and temperature during operation.

    For two-phase reservoir flow, our investigation suggests that developing a single surrogate that simultaneously covers both single- and two-phase geothermal systems under high-dimensional inputs and outputs would require more complex physics (e.g., phase appearance/disappearance, relative permeability and capillarity) and a much broader simulation dataset to represent the enlarged parameter space and strongly coupled dynamics. This expansion would markedly

increase model complexity and training costs. Therefore, it is more practical to develop a dedicated surrogate for two-phase geothermal systems, which provide a better balance between computational costs and predictive capability. The present PCNO framework also provides a foundation for this extension. Specifically, as a future step, a two-phase reservoir surrogate can be developed with the same interface and integrated into the PCNO-based evaluation workflow to enable phase-regime-specific deployment across a broader range of geothermal systems. We have clarified this limitation and identified an extension to full multiphase reservoir formulations as an important direction for future work in the revised Discussion section.

Regarding the wellbore dynamics, we agree that the original formulation was simplified. In the revised version, we have upgraded the wellbore module to a two-phase flow formulation that accounts for phase change, pressure losses, and thermal losses along a wellbore, improving the fidelity of predicted wellhead conditions.

John O'Sullivan

Reviewer #2 (Remarks to the Author):

I co-reviewed this manuscript with one of the reviewers who provided the listed reports. This is part of the Nature Communications initiative to facilitate training in peer review and to provide appropriate recognition for Early Career Researchers who co-review manuscripts.

Thank you for noting this. We are grateful for your careful evaluation and constructive feedback provided in the review reports.

Reviewer #2 (Remarks on code availability):

19. The authors have not provided a dedicated README file with installation or execution instructions; however, the code structure is intuitive and well-organized, which made it straightforward to run. The scripts executed successfully with only a minor warning related to NumPy version compatibility:

    /code/PCNO_Ele_Eco_Module.py:212: DeprecationWarning: Conversion of an array with ndim > 0 to a scalar is deprecated, and will error in future. Ensure you extract a single element from your array before performing this operation. (Deprecated NumPy 1.25.)

    This warning does not affect the current functionality or reproducibility of results. Overall, the code appears to be a usable and reproducible resource for the community, though adding a concise README file with environment setup and execution steps would further improve accessibility.

    Response: Thank you for this comment.

We have uploaded the updated code package and added a README file. In addition, We have addressed the reported NumPy deprecation warning by explicitly extracting scalar values where appropriate.

Reviewer #3 (Remarks to the Author):

This study demonstrated the effectiveness of physics-constrained neural operator (PCNO) in simulating geothermal reservoirs. It presents novel and intriguing results for geothermal development. The authors demonstrated high computational accuracy and significant computational efficiency using the proposed PCNO method. A function to perform economic evaluations after PCNO-based emulators has also been implemented. While the research is considered to have high impact, concerns described below remain.

Thank you for the positive comments and valuable suggestions. These comments are very helpful for revising and improving our paper, as well as in guiding our research. The detailed point-by-point responses according to your comments are shown below.

20. It seems that this paper lacks any description of how the physical laws were learned, leaving doubts about whether predictions that satisfy the physical laws were made.

Response: Thank you for this comment.

In the revised Method S3, “Physics informed loss”, we have described how the PCNO learns and enforces physical laws. In brief, the PCNO embeds the governing physics through a physics-informed loss that incorporates (i) mass and energy conservation, (ii) thermophysical property relations, (iii) radial inflow (well-reservoir coupling) equations, and (iv) wellbore dynamics.

Specifically, the network first predicts reservoir pressure and temperature fields from the input parameters. These predicted fields are then substituted into the governing conservation equations, and the corresponding mass- and energy-balance residuals are evaluated (Eqs. 8-9 in the main text). These residuals are added to the total loss so that violations of conservation are explicitly penalized during training. During this process, fluid properties (e.g., density, viscosity, and enthalpy) are computed as functions of local pressure and temperature and are used consistently in the evaluation of these residuals, ensuring that the PDE terms are coupled to realistic thermophysical behaviour.

In addition, the predicted reservoir pressures are coupled to the wells through radial inflow relations to obtain downhole quantities such as injector BHP. These downhole conditions are then propagated to the surface with a two-phase wellbore model that accounts for phase change, pressure losses, and heat losses along the wellbore, which gives surface production temperature and thermal output. The discrepancies between these task variables and the corresponding CMG-STARS outputs (Eq. 10) are also included in the loss, which constrains the model to be consistent with well-reservoir coupling and wellbore physics, not only with reservoir states.

By minimizing a combined objective that contains both data-driven terms and physics-based residuals (Eq. 11), the PCNO is trained to produce predictions that are accurate with respect to the reference simulations and consistent with the embedded physical laws. This design ensures that the learned operator is guided toward solutions that are not only accurate in a statistical sense but also consistent with the underlying physical laws included in the framework.

21. This study described that they used a previously validated geothermal reservoir model as data, but I could not find details of this model in the cited research. Based on the descriptions in the paper, the numerical model used is not a real-world field model but a simplified virtual model. This permeable structure does not model volcanic geothermal regions, which are typical targets in commercial operations, but rather appears to be a simplification of sedimentary layers.

    Response: Thank you for this comment**.**

    In this work, the training data are generated with CMG-STARS using a base reservoir model whose settings are derived from our previously validated CMG-based geothermal models. This base model is used to ensure that the underlying model structure and physics options are consistent with established geothermal modelling practice. It then serves as a foundation from which a large ensemble of synthetic cases is created by systematically varying key geological and operational parameters. A detailed description of this base model is now provided in Methods S1.

    We agree that the numerical model used here is not a real-world volcanic field model but a “virtual” reservoir. This is intentional. The aim of this work is to develop a fast, generalizable, and physics-informed surrogate that can support systematic performance evaluation across different geothermal reservoirs. Because high-quality, long-term field datasets with well-constrained geology, controls, and monitoring are still limited, especially for large ensembles, we follow a common practice in surrogate modelling and generate training data from controlled numerical experiments. Specifically, we first identify key sensitive reservoir and operational parameters from our previous work and the published geothermal literature, then sample these within representative ranges using the base model to build a training ensemble. This approach ensures physical consistency of the dataset while enabling sufficient coverage of the parameter space required for model training.

    To further improve generalization within this conceptual class, the revised version adopts an expanded input space and updated physics. As described in Response 0, the computational domain is extended to a fully 4D dimensions (3D spatial and temporal); the permeability range is extended from 50-300 mD to 1-300 mD; the inputs now include pressure and temperature gradients, well depths, and time-varying injection schedules; and the wellbore model is upgraded to a two-phase flow formulation. These updates broaden the range of reservoir conditions and operating strategies represented in the training set while keeping a coherent physical framework.

Within these training ranges and conceptual assumptions, the PCNO is not limited to the specific realizations used during training, but can predict the behaviour of systems whose reservoir and operating parameters fall within the trained domain. To demonstrate this, we apply the PCNO to a real EGS reservoir case that was not included in the training dataset, and the results show high accuracy with a reference result in previous study (Figure S8).

We also acknowledge that the current PCNO has limitations. These limitations, along with the advantages and implications, have been clarified in the revised Discussion section.

22. In geothermal development, production wells and reinjection wells are typically placed at different depths and designed to prevent reinjected water from overcooling the reservoir. However, in this numerical model, these wells are placed at the same depth. Given these points, it remains questionable whether the numerical model used in this study can be recognized as a realistic geothermal system.

Response: Thank you for this comment.

To address this concern and improve the realism of the model, we have updated the framework from a 3D setting with two spatial dimensions and time to a full 4D setting with three spatial dimensions and time, and we have introduced the well depths of both injectors and producers as new input variables. Therefore, injection and production wells are no longer constrained to the same depth. Their completion depths are now sampled within a target reservoir interval, with injectors placed shallower than producers. By including these new inputs, the PCNO can now learn and predict geothermal development scenarios with vertically separated injection and production intervals. We have updated the manuscript and Method S1 to describe this revised well configuration, and have also reported production performance for cases with different injector-producer depths in Figures 2, S4, and S5.

Reviewer #4 (Remarks to the Author):

This was a very thoroughly prepared manuscript. The motivation, methodology and results are very relevant and timely. However, there are a few comments raised that need to be addressed to improve the manuscript thus requiring a major revision.

Thank you for your careful review and valuable recommendations on this manuscript, which significantly enhance the quality of this piece of work. According to your subsequent remarks, we have furnished detailed responses, illustrating the resolution of each comment below.

Major comments:

Computational Speedup

23. Hardware details missing: CPU/GPU specs, memory, and environment should be stated to validate the speedup ratio.

Response: Thank you for this comment.

We have added the hardware details for both training and testing, which are now described in the "Computational speed-up" section of the manuscript and in Table S3 of the Supplementary Information.

Data Preprocessing:

24. Sampling method unspecified: How the 6,000 cases were sampled (uniform, Latin Hypercube, Monte Carlo) is not stated.

Response: Thank you for this comment.

In the revised version, we have regenerated the dataset and retrained the model, where the dataset now contains 9,450 samples. The input space is sampled using a Monte Carlo strategy. Specifically, for each case, all reservoir and operational parameters are drawn independently from uniform distributions over their prescribed ranges. The resulting parameter sets are then used as inputs to CMG-STARS to generate the ensemble for training and testing. This sampling procedure has been added to the "Dataset overview" and "Recoverable geothermal energy potential assessments" sections.

Model training:

25. For model training hardware and runtime: Training time and resource consumption are not discussed.

Response: Thank you for this comment.

We have added the hardware specifications and the corresponding training runtimes, together with a summary of the computational costs, which are now described in Table S3.

Supplementary method 1- Numerical simulation settings

26. No boundary-condition details: lateral/vertical BCs (constant T, no-flow, etc.) should be specified.

Response: Thank you for this comment.

In the revised manuscript, we provide a more detailed description of the boundary conditions used in the numerical model, as illustrated in Method S1.

Model

27. Validation only on synthetic CMG data, no field case or empirical benchmark - Include one field-scale validation (e.g., Dixie Valley, Soultz) or cross-compare with published field data.

Response: Thank you for this comment.

This comment highly improves the quality of our work. To address this concern, we have added a field-scale validation using the Qiabuqia geothermal field. In the revised version, we compare

PCNO predictions with previously published and widely cited results for this field, as shown in Figure S8.

Discussion

28. The authors mention that the framework can be applied to simple EGS systems. Please provide addition context to what simple EGS systems mean, as the porosity and permeability fields of EGS differ from conventional hydrothermal geothermal systems.

Response: Thank you for this comment.

We agree that EGS reservoirs often exhibit porosity-permeability structures that differ from conventional hydrothermal systems. In the revised version, we have extended the permeability range from 50-300 mD to 0.1-300 mD and expanded the corresponding porosity range. Within these ranges, the PCNO is not tied to the specific training realizations and can be applied to reservoirs whose effective porosity and permeability values lie inside the trained domain. This update allows the dataset to better cover the lower-permeability conditions and effective properties that are relevant for some EGS settings.

When we refer to "simple EGS systems", we mean EGS representations in which the complex fracture network is not resolved explicitly but is captured through effective-medium treatments that are widely used in numerical EGS modeling when fracture geometry and properties are poorly constrained. In such cases, stimulation effects or engineered fracture zones are often represented by assigning enhanced effective permeability to a stimulated region or to grid cells that connect wells. For example, in the widely cited study of the Qiabuqia EGS reservoir by Lei et al.[2], the stimulated zone was represented as a region with elevated permeability relative to the surrounding formation. Similarly, Kumawat and McLennan et al.[3] used a simplified planar approach to represent fractures for the Utah FROGE project, where the fracture aperture was approximated by the grid-block size and the effective permeability was tuned to match target fracture conductivities. Comparable permeability-multiplier or equivalent-permeability approaches have also been widely adopted in other EGS and oil and gas studies[4–6].

Consistent with this definition, we use the PCNO to evaluate the Qiabuqia EGS in which the stimulated region is represented through an effective permeability enhancement, and we compare the PCNO predictions against the published results for this field. The description of this setup and the comparison are provided in Figure S8. We also note that the current PCNO cannot yet accurately represent more complex, spatially non-uniform engineered fracture networks. This limitation, together with its reasons, has been discussed in detail in the revised Discussion section.

29. The thermal stresses associated with geothermal systems are not negligible. Kindly provide some comments on why geomechanics is not considered in the model, and what is the implication for real geothermal systems when using the PCNO framework.

Response: Thank you for this comment.

We agree that thermal stresses can be important in geothermal systems. In this work, we focus on developing an efficient and reliable framework for coupled thermal-hydraulic processes, which represent the dominant controls on heat extraction and energy production in many system-level applications. Incorporating geomechanics would require solving additional stress-strain equations and introducing complex thermoelastic and rock-mechanical parameters. This would substantially increase input dimensionality, the size of the required training ensemble, and the computational cost of data generation and training.

A similar trade-off is often made in geothermal simulation studies, where TH-only mechanisms are used when the primary objective is to characterize heat extraction and pressure evolution at the reservoir scale and to maintain computational feasibility. From a surrogate-modelling perspective, the current TH-focused formulation is intended to strike a balance physical fidelity, input-output complexity, and computational efficiency, so that scalable learning remains practical while key system-level responses can still be captured. Therefore, geomechanical effects are not included in the present PCNO implementation.

Regarding its implications, the PCNO is intended for geothermal systems whose reservoir conditions fall within the ranges covered by the training data. Within this scope, it can serve as a fast and robust screening and decision-support tool for tasks such as scenario exploration, probabilistic assessments, uncertainty analyses, and preliminary design. By predicting system behaviour at multiple levels, from reservoir fields to task-specific metrics, it can provide a multi-dimensional view of geothermal performance (e.g., production volume, system sustainability, operational feasibility, cost indicators, and suitability for different end-use applications). For a given target reservoir, the PCNO can quickly evaluate subsurface and surface performance across many operating strategies and identify plausible ranges of key sensitive parameters, which helps narrow the design space and supports decisions related to development prioritization, end-use selection and surface facility design.

We also acknowledge that the current PCNO is not directly applicable to more complex and strongly coupled systems, such as strongly two-phase reservoirs, fault-dominated structures and highly non-uniform engineered fracture networks. In these settings, the present framework should not replace detailed full-physics models and is suitable for coarse screening. However, the PCNO framework provides a basis that can be adapted or retrained for these classes of systems. For example, a dedicated two-phase geothermal surrogate could be developed with parameter ranges tailored to such conditions and then used together with the present PCNO to cover a wider spectrum of resource types. Similarly, once sufficient high-fidelity data are available in a form that supports systematic parameterisation of faults or complex fractures, these features can be incorporated into extended surrogate models that build on the current framework.

We have clarified the advantages, implications, and limitations of the PCNO, as well as the future directions in the revised Discussion section.

Section wise minor comments:

Introduction:

30. Line 89 – Typo – ‘Neural’ instead of ‘Nueral’

Line 90 – Typo – “Conservation” instead of “Conversation”

Response: Thank you for this comment.

We have corrected both errors in the revised manuscript.

Results- Dataset Overview:

31. Range justification: Some ranges (e.g., 1.8×10^6 J/(m·day·°C) for thermal conductivity) may not be physically consistent with typical geothermal rock values — clarification needed on units or scaling.

Response: Thank you for this comment.

We found that the values for rock thermal conductivity and volumetric heat capacity were swapped by mistake in the previous manuscript. Specifically, the value 1.8×10^6 J/(m^3·°C) corresponds to the rock heat capacity rather than the thermal conductivity. We have corrected this error and updated Table 1 accordingly to ensure that the reported units and parameter ranges are physically consistent.

32. Sampling strategy missing: No mention of sampling method (e.g., Latin Hypercube, uniform random, Sobol sequence).

Response: Thank you for this comment.

We have added the sampling strategy in the revised manuscript. Specifically, we use a Monte Carlo sampling approach to generate realizations of reservoir and operational parameters, where each parameter is independently drawn from a uniform distribution over its specified range. This is now clarified in the “Dataset overview” and “Recoverable geothermal energy potential assessments” sections.

33. Training–validation split rationale: A 500-sample validation set (8%) may be small for hyperparameter optimization; mention if cross-validation was used.

Response: Thank you for this comment.

In the revised version, we re-generated the dataset and re-trained the model using a total of 9,450 samples, including 7,350 for training, 1,050 for validation, and 1,050 for testing. We also adopted a k-fold cross-validation strategy during training and validation to improve robustness. Specifically, we pooled 8,400 samples and split them into six folds. In each epoch,

five folds were randomly selected for training, and the remaining fold was used for validation. Validation started from epoch 30, and during epochs 1 to 29 only training was performed. These details have been clarified in Method S4.

34. Simulator assumptions omitted: CMG-STARS setup (boundary conditions, and equation of state) should be summarized or referenced to ensure reproducibility.

    Response: Thank you for this comment.

    In the revised version, we have added a more detailed description of the CMG-STARS model setup, including the initial and boundary conditions and the treatments of water properties under different pressure and temperature conditions. These details are now provided in Method S1.

35. Discuss whether CMG-STARS runs include transient wellbore coupling or only reservoir-scale heat transport, if so, this avoids the need to constrain using equations 9 and 10 for wellbore modeling.

    Response: Thank you for this comment.

    In our CMG-STARS runs, transient wellbore coupling is indeed included. However, once CMG-STARS is replaced by the surrogate, it only predicts reservoir temperature and pressure fields. if no additional wellbore equations are incorporated, the surrogate can reproduce reservoir evolution but cannot resolve wellbore dynamics or surface-level performance from its own outputs. This limitation is common to conventional FNO-based surrogates and many existing geothermal surrogate models, which focus solely on reservoir states.

    For this reason, we include wellbore dynamic equations within the framework. In the revised version, these equations form a two-phase wellbore model that accounts for phase change, pressure drop, and thermal loss along the wellbore and computes surface fluid temperature and thermal energy output. In each training step, the reservoir pressure and temperature is predicted firstly and then passed to the wellbore equations for computing surface production temperature and thermal output. These predicted metrics are then compared with the corresponding CMG-STARS results, and the mismatch is included as one component of the physics-informed loss.

    By minimizing a combined loss that contains both data-driven terms and physics-based residuals, the PCNO is trained not only to match CMG-STARS outputs but also to remain consistent with the embedded wellbore and reservoir physics. We have described this training procedure and the role of the wellbore equations in more details in Method S3, “Physics informed loss”.

36. PCNO model architecture – None

    Response: Thank you for this comment.

In the revised version, we have added a detailed description of the PCNO model architecture and provided a schematic diagram, as shown in the Figure S3.

Model prediction performance

37. Spatial averaging: Unclear whether reported errors are grid-averaged, volume-weighted, or well-centered.

    Response: Thank you for this comment.

    For the reservoir pressure and temperature fields, errors are first computed at each cell on the full 80 × 80 × 5 grid. The reported scalar metrics (such as a mean squared error and a mean relative error) are then obtained by taking simple averages over all grid cells. In this sense, the reported errors are grid-averaged and are not volume-weighted or well-centered. We have clarified this in the manuscript.

38. Temporal generalization: Model performance beyond 20 years or unseen injection schedules not tested.

    Response: Thank you for this comment.

    In the current study, the PCNO is trained and validated for a 20-year operation period to reflect typical project planning horizons in geothermal development. All results reported in the manuscript are confined to this 20-year window. Accordingly, we do not claim predictive capability beyond 20 years, and the model has not been tested for temporal extrapolation outside the training range.

    This limitation is not unique to our framework but reflects a general constraint of data-driven models trained on fixed time horizons. The current PCNO is designed to capture the complex spatial-temporal interactions within this 20-year window with high fidelity, which is critical for early-stage screening, system design, and control optimization. Extending the prediction capability to longer periods would require additional simulation data and possibly different neural architectures such as recurrent or attention-based models. These models are more suitable for long-horizon time-series forecasting but rely on accurate short-horizon predictions as input. The current PCNO can serve as a strong foundation for such future extensions.

    For injection schedules, we have expanded the training data to include both constant-rate and time-varying injection schedules. In the revised version, we present a specific case study using a time-varying injection profile that was not part of the training set. As shown in Figure S6, the PCNO accurately reproduces the resulting pressure, temperature, and well response under this unseen schedule, indicating that the model can handle specific variations in injection control within the defined input domain.

    We have clarified these points in the revised manuscript and identified long-horizon prediction as a valuable direction for future development.

Recoverable geothermal energy potential assessments:

39. Computational feasibility: Simulating 2,000 ensembles rapidly is mentioned but not quantified (runtime or computational resource).

    Response: Thank you for this comment.

    In the revised 'Recoverable geothermal energy potential assessments' section, we now quantify the runtime and computational resources required for the ensemble predictions. Specifically, 2,000 cases are evaluated on a single NVIDIA A100 PCIe GPU, with total inference completed in approximately 650 s. Based on the tests shown in Figure S9, running 2,000 cases on an AMD Ryzen 9 5950X CPU by using CMG-STARS would require around 800,000 s. This indicates that the PCNO provides substantial computational speed-up for assessing these 2,000 cases.

40. Unit consistency: Some plots (MWth/MWe) and energy totals (GWh) could use unified scaling for clarity.

    Response: Thank you for this comment.

    In the revised version, we have re-plotted the relevant figures using a consistent unit scaling for installed capacities (MWth and MWe) and total energy outputs (GWh). These updated results are presented in Figure 5 of the manuscript.

Economic evaluation and development plan optimization:

41. Composite score definition unclear: Weighting between technical and economic terms should be detailed.

    Optimization Transparency: Composite score weighting (technical vs economic) undefined. Readers cannot reproduce optimization outcomes. Present weighting formula or Pareto front of LCOE vs thermal output.

    Response: Thank you for these comments.

    In the revised version, we have added a detailed description of the composite score function, which includes the weighting scheme used to balance technical and economic metrics, as well as the normalization procedure applied to ensure comparability across scenarios. These details are presented in Method S5.

42. Scaling realism: Thousands of cases evaluated, but computational time or hardware requirements are not quantified.

    Response: Thank you for this comment.

    In the revised version, we have included a detailed description of computational time and hardware requirements in the 'Computational speed-up' section, Figure S9, and Table S3.

43. Currency year: Costs lack reference year or regional adjustment (e.g., USD 2024).

Response: Thank you for this comment.

In the revised manuscript, we have stated the currency year and adjustment method. In specific, all costs are converted to 2025 USD using a conversion index based on the U.S. Bureau of Labor Statistics Consumer Price Index Inflation Calculator, as described in the 'Integrated economic module for financial assessment' section.

Computational speed-up:

44. Dataset loading time: It's unclear if preprocessing and post-processing times are included in the 50 s measurement.

Response: Thank you for this comment.

In the revised "Computational speed-up" section, we now state that the reported prediction times include loading the preprocessed dataset into memory and the forward inference, so the numbers reflect the actual runtime from data-in-memory to prediction.

We also provided a detailed description of computational time in Table S3. Specifically, we clarify that both the reported training and prediction times include dataset loading but exclude the one-time preprocessing step that converts raw simulation outputs into the tensor format used for PCNO training and testing. In our workflow, this preprocessing is performed once before training and typically takes from seconds to a few minutes, depending on dataset size. The post-processing time needed to convert PCNO outputs into task-specific formats is also excluded, as it depends on an application and follows the same convention used when reporting the runtime of traditional numerical simulations. For CMG-STARS, the reported time cost includes the simulation period and model initialization between runs, but excludes data preprocessing, post-processing, and model construction effort. This follows the same convention commonly used when reporting runtimes for traditional numerical simulators and ensures a consistent basis for comparing CMG-STARS and PCNO.

45. No discussion of training time: Readers may question total cost (training + inference) versus repeated simulations.

Response: Thank you for this comment.

In the revised version, we have included a detailed description of computational time including both training and inference times, as illustrated in Table S3.

46. Energy efficiency: Power consumption savings (CPU-hours, CO_2 footprint) could further strengthen the argument.

Response: Thank you for this comment.

In the revised Table S3, we now report both training and inference times for surrogate models, together with the corresponding CMG-STARS runtimes. Specifically, predicting 1,050 cases using the PCNO requires approximately 0.022 kWh using the NVIDIA A100-PCIe GPU, while the equivalent simulations using CMG-STARS on the AMD Ryzen 9 5950X CPU require over 12.9 kWh. We also clarify that the PCNO training is a one-time offline cost, whereas ensemble predictions are called repeatedly. Even after accounting for the one-time energy cost of training the PCNO, the energy required to train PCNO is rapidly offset once the surrogate is applied to large ensembles, because each subsequent evaluation requires only a small fraction of the energy needed for a full numerical simulation and remains valid for the geothermal reservoirs whose conditions fall within the ranges of the training data. The details of these analyses are provided in the Table S3.

Discussion:

47. Line 386 – 'Spatial heterogeneity' instead of 'spatial heterogenous'.

    Line 435 – Typo - "FCNO" instead of "PCNO"

    Response: Thank you for this comment.

    We have corrected these two typos in the revised manuscript.

Methods: Datasets development and preprocessing

48. Validation link to field data: The dataset is simulation-based only—no mention of comparison or calibration to real geothermal data.

    Response: Thank you for this comment.

    In the revised Supplementary Information, we have added a field-scale cross-comparison using the Qiabuqia geothermal field. Specifically, we compare PCNO-based predictions with previously published and widely cited results for this site. The comparison is presented in Figure S8.

49. Temporal resolution: The frequency of recorded outputs over 20 years is not indicated (annual, quarterly?).

    Response: Thank you for this comment.

    The frequency of recorded outputs over 20 years is annual. We have clarified this in the "Datasets development and preprocessing" section of the manuscript.

50. Fixed pressure gradient assumption: Simplifies depth-pressure relation—should mention if sensitivity analysis confirms minimal bias.

    Response: Thank you for this comment.

In the revised version, we no longer assume a fixed pressure gradient. Instead, the pressure gradient is treated as an input feature and is allowed to vary between 7 and 15 MPa/km, as summarized in Table 1. This approach enables the PCNO to capture different depth–pressure relationships across geothermal settings and reduces potential bias associated with a fixed-gradient assumption.

Evaluation metrics for model training:

51. Weight selection unexplained: The rationale or tuning process for w_1–w_{11} is not discussed.

    Response: Thank you for this comment.

    In the revised Supplementary Information, the weights used during training have been updated in Table S1. Given the complexity and strong coupling of physical processes in geothermal systems, conventional hyperparameter tuning methods or direct transfer from previous studies are difficult to apply when selecting individual loss weights. Instead, the weighting strategy for Phases 1 and 2 was determined empirically by monitoring the loss trajectories over the first 50 epochs. The weights were adjusted to ensure stable convergence and a consistent decrease in the total loss. The Phase 3 strategy was then designed based on the behavior observed in the earlier phases and the resulting model performance. As a result, this approach offers a practical guideline for constructing surrogate models in other complex subsurface energy systems. We have also included a brief discussion of this in the revised Discussion section.

PCNO model training procedure:

52. Optimization details are missing: No mention of optimizer type (Adam, RMSprop), learning rate, or batch size.

    Response: Thank you for this comment.

    In the revised Supplementary Information, we have added a detailed description of the model training configuration in Method S4. This includes an optimizer type, a learning rate, and other key training parameters. In addition, Table S2 has been updated to summarize the network architecture and outputs across different training phases, and reports the batch size used.

Embedded power estimation module for geothermal applications:

53. Empirical origin unclear: The source of Eq. (13) (reference 36) should be described—whether derived from regression on global plant data or literature fits.

    Equation Validity - $\eta = 0.1268 \ln(T\_pro) - 0.4915$ can yield $\eta > 1$ for high T. Cap $\eta \leq 0.25 - 0.30$ and cite the empirical source range.

    Response: Thank you for these two comments.

    In the revised manuscript, we have updated previous Eq. (13) to a published empirical correlation and clarified its origin in the text, which is changed to Eq. (12) in the revised version.

Specifically, this equation is adopted from Zarrouk et al.[7], where the authors established an enthalpy-efficiency relationship by performing regression analysis on a large dataset of reported performance metrics from geothermal power plants worldwide. This formulation captures general trends observed across different plant types and geographic regions, and provides a practical basis for estimating conversion efficiency in techno-economic assessments.

54. Assumed constants: Capacity factor (0.85) and zero efficiency below 75 °C could vary regionally or by plant type; sensitivity analysis is missing.

Response: Thank you for this comment.

In the revised Supplementary Information, we have conducted a sensitivity analysis on the capacity factor and the temperature threshold for power generation, as illustrated in Figure S10.

Integrated economic module for financial assessment:

55. Currency and base year unspecified: All cost functions need normalization (e.g., USD 2024).

Response: Thank you for this comment.

In the revised manuscript, we have stated the currency year and adjustment method. In specific, all costs are converted to 2025 USD using a conversion index based on the U.S. Bureau of Labor Statistics Consumer Price Index Inflation Calculator, as described in the 'Integrated economic module for financial assessment' section.

56. O&M labor split: "75 % labor" could be clarified—does this apply annually or as average over lifetime?

Response: Thank you for this comment.

In our formulation, the 75% and 25% factors in Eqs. (22) and (23) refer to the *annual* split of O&M labor costs and are applied consistently in each year of the project lifetime. These values are not lifetime averages, but fixed fractions used to allocate the yearly O&M labor cost between surface facilities and the wellfield, following previous geothermal techno-economic studies. The annual labor costs are calculated using the empirical formulas provided in Eqs. (24) and (25), which express O&M labor as a function of installed capacity.

57. Line 614 - Typographical issues: "include expenditures" → "includes expenditures".

Response: Thank you for this comment.

We have corrected this typographical issue in the revised manuscript

Supplementary Methods - Supplementary Method 1: Numerical modeling settings:

58. Grid simplification: Single-layer (1 z-cell) may under-represent vertical gradients or convection effects.

Response: Thank you for this comment.

In the revised version, we have re-created the training dataset and re-trained the PCNO model using a fully 3D reservoir grid with 80 × 80 × 5 cells. This configuration allows the simulations to capture vertical pressure and temperature gradients, as well as fluid and heat transport in both the horizontal and vertical directions. The updated grid structure improves the physical realism of the training data and enhances the model ability to represent 3D subsurface dynamics. We have revised the corresponding sections in the manuscript to reflect this change.

59. Thermal equilibrium assumptions: initial conditions (T–P distribution) not explicitly stated—hydrostatic? steady-state?

Response: Thank you for this comment.

In the revised Supplementary Information, we have added a description of initial conditions in Method S1. The initial reservoir pressure is assumed to be hydrostatic and is computed from the reservoir depth together with a specified pressure gradient. The initial reservoir temperature is assumed to be in a steady state and is calculated using a prescribed geothermal gradient.

60. Injection rate units: "kg/m³" likely meant "kg/s" or "kg/s per well"; should be checked.

Response: Thank you for this comment.

You are correct; the injection rate unit should be kg/s. We have corrected this in the revised manuscript and Supplementary Methods, replacing the incorrect unit "kg/m³" with "kg/s" for all injection rate expressions.

61. No timestep description: temporal resolution and solver settings (implicit/explicit) omitted.

Response: Thank you for this comment.

In the revised Supplementary Information, we have added a detailed description of the timestep and solver settings used in the numerical simulations (Method S1). In specific, each simulation begins with an initial timestep of 0.1 day, with a maximum limit of 10 days to maintain numerical stability and capture production transients. An adaptive implicit method is employed, which dynamically switches between fully implicit and Implicit Pressure-Explicit Saturation (IMPES) schemes depending on local conditions. Grid blocks with porosity less than 0.01, aquifer connections, heaters, or active wells are always treated using a fully implicit formulation. This hybrid scheme improves numerical robustness while maintaining computational efficiency.

62. Line 80 – Reference missing for injection rate.

Response: Thank you for this comment.

In the revised Supplementary Information, we have added references to support the selected range of injection rates (Method S1).

Supplementary Method 2: Governing Equations Integrated into the PDCO Framework:

63. Line 97 and 98 - Typographical inconsistency: Framework name alternates between PDCO and PCNO should be consistent.

Response: Thank you for this comment.

We revised all these kinds of typos and set the name to be a uniformed name ‘PCNO’.

64. Radial inflow model (Eq. 6): The λ term is ambiguous used for relative mobility, but notation overlaps with thermal conductivity.

Equation dimensionality: Eq. (6) uses “q (kg/m³)”—should likely be mass flow rate (kg/s) or volumetric flow rate (m³/s).

Response: Thank you for these comments.

In the revised Supplementary Information, we have replaced the relative mobility symbol λ with k_{rf}/μ_f to avoid confusion with thermal conductivity. We have also corrected the unit of q in Method S1, Eq. (6) to kg/s to clearly indicate that it represents a mass flow rate.

65. Fluid property models: Piecewise viscosity function (Eq. 12) may cause discontinuities near thresholds (40 °C, 100 °C); smoothing suggested.

Response: Thank you for this comment.

In the revised Supplementary Information, we have updated the water viscosity correlation to avoid discontinuities at the previous threshold temperatures, as shown in Method S2, Eq. (29).

66. Line 172 - Typo – Supplementary table 1 should be stated instead of 2.

Typo – At supplementary table 1 change the reference equation 11 to equation 13 for the co-efficients A to H.

Response: Thank you for this comment.

In the revised version, we have removed the previous Supplementary Table 1 to improve clarity and conciseness. The values of the relevant parameters are now stated directly in the text following the corresponding equations.

Supplementary Method 3: Physics-Constrained Neural Operator Structure.

67. Table 4 comparison: ANN-FNO faster than FNO + GPF but less accurate—brief rationale would help (overhead vs complexity).

Response: Thank you for this comment.

In this revised Supplementary Information, we have conducted additional tests comparing the performance of different model configurations, and the results are summarized in Table S3.

Reviewer #4 (Remarks on code availability):

68. The code ran successfully with just a minor warning "+ python -u PCNO_Ele_Eco_Module.py | /code/PCNO_Ele_Eco_Module.py:212: DeprecationWarning: Conversion of an array with ndim > 0 to a scalar is deprecated, and will error in future. Ensure you extract a single element from your array before performing this operation. (Deprecated NumPy 1.25.)". Guidance on how to extract the single array.

Response: Thank you for this comment.

We have uploaded the updated code package and added a README file. In addition, We have addressed the reported NumPy deprecation warning by explicitly extracting scalar values where appropriate.

References

1. Wang, Y. *et al.* Uncertainty quantification in a heterogeneous fluvial sandstone reservoir using GPU-based Monte Carlo simulation. *Geothermics* **114**, 102773 (2023).
2. Lei, Z. *et al.* Exploratory research into the enhanced geothermal system power generation project: The Qiabuqia geothermal field, Northwest China. *Renew. Energy* **139**, 52–70 (2019).
3. Piyush Kumar Kumawat *et al.* Simplified Fracture Model for Numerical Simulation of EGS: A Utah FORGE Case Study. in *50th Workshop on Geothermal Reservoir Engineering* (California, United States, 2024).
4. Llanos, E. M., Zarrouk, S. J. & Hogarth, R. A. Numerical model of the Habanero geothermal reservoir, Australia. *Geothermics* **53**, 308–319 (2015).
5. Cherry, J. Optimization Strategies for Shale Gas Asset Development.
6. Park, J. & Janova, C. Stimulated Reservoir Volume Characterization and Optimum Lateral Well Spacing Study of Two-Well Pad: Midland Basin Case Study. *Geofluids* **2020**, 1–18 (2020).
7. Zarrouk, S. J. & Moon, H. Efficiency of geothermal power plants: A worldwide review. *Geothermics* **51**, 142–153 (2014).

Responses to Editor and Reviewers

Dear Editor and Reviewers:

We sincerely thank you for your constructive comments on our manuscript. We are pleased that the manuscript has been considered in principle for acceptance in ***Nature Communications***. We have carefully addressed all the minor comments and revised the manuscript accordingly. Detailed responses are provided below, and all corresponding changes have been incorporated into the revised manuscript.

Sincerely yours,

Zhenqian Xue, Jianfei Bi, Haoming Ma, Zhe Sun, Zhangxin Chen

Reviewer #1 (Remarks to the Author):

The authors are to be congratulated for their thorough and considered responses to the reviews. The revised article is considerably improved for their diligent hard work. Well done!

I have one comment on their responses and one small correct listed below.

Thanks for your positive and encouraging comments on our revised manuscript. We greatly appreciate your recognition of our efforts. We have carefully considered your additional comments, and have addressed them accordingly, as detailed below.

1. Response number (1)

   The authors still do not have this quite right. It is true that "the standard workflow in many geothermal reservoir studies, where a separate natural-state model is used to establish stable pressure and temperature fields, which are then taken as initial conditions for dynamic production simulations." But in large-scale, hot, convecting geothermal systems it is not possible to "apply initial temperature and pressure distributions that represent the natural state" and you must "re-simulate long-term natural convection and background flow" to ensure it is consistent with each sample model's permeability distribution and boundary conditions.

   It is also true that often (but not always) "During active production, the pressure drawdown and overpressure around wells generate pressure-driven flow that is much stronger than the slow background flow caused by natural convection." However, production temperature decline can be small and the effect an assumed initial temperature distribution being

inconsistent with the model's permeability distribution and boundary conditions may be non-negligible in comparison.

However, given the content of the article, the careful wording the authors have used and the example models presented, I am happy enough with the authors' explanation.

For future reference though, in cases where the initial temperature distribution is complex with strong convection and vertical flows, you must always rerun natural state simulations to determine the correct initial conditions.

Response: Thank you for this comment.

We fully agree that, in large-scale geothermal systems with strong natural convection and complex vertical flow structures, the natural state must be re-simulated to ensure consistency between temperature and pressure fields, permeability distribution, and boundary conditions.

In future studies involving complex initial temperature distributions with strong convection and vertical flows, we will carefully incorporate natural-state re-simulation to improve the robustness and physical consistency of the initial conditions.

We sincerely thank the reviewer for this valuable suggestion.

2. I spotted one small error also: line 226: "simplify" not "simplified"

   Response: Thank you for this comment.

   We have corrected this error in the revised manuscript.

Reviewer #2 (Remarks to the Author):

I co-reviewed this manuscript with one of the reviewers who provided the listed reports. This is part of the Nature Communications initiative to facilitate training in peer review and to provide appropriate recognition for Early Career Researchers who co-review manuscripts.

Reviewer #2 (Remarks on code availability):

Yes, the code is running correctly

Response: We sincerely thank you for your contribution to the co-review process and for confirming that the code is running correctly.

Reviewer #4 (Remarks to the Author):

The authors have thoroughly addressed the comments previously raised. I recommend the manuscript for publication.

Response: We sincerely thank the reviewer for the positive evaluation of our manuscript and for recommending it for publication.
